# Unlocking Cd(II) biosorption potential of *Candida tropicalis* XTA 1874 for sustainable wastewater treatment

**DOI:** 10.1038/s41598-024-66336-y

**Published:** 2024-07-08

**Authors:** Kaustav Bhattacharyya, Neelanjan Bhattacharjee, Debrup Sen, Tapan Kumar Lai, Ananyo K. Ghosh, Ritesh Ranjan Pal, Subhadeep Ganguly

**Affiliations:** 1 Department of Physiology, Vidyasagar College, 39, Sankar Ghosh Lane, Kolkata, West Bengal 700006 India; 2 Department of Mechanical Engineering, University of Alberta, Room 4-31F9211 116 Street NW, Edmonton, AB T6G 1H9 Canada; 3 Department of Zoology, Vidyasagar College, 39, Sankar Ghosh Lane, Kolkata, West Bengal 700006 India; 4 Department of Chemistry, Vidyasagar Metropolitan College, 39, Sankar Ghosh Lane, Kolkata, West Bengal 700006 India; 5 School of Biological Sciences, Indian Association for the Cultivation of Science, 2A&2B Raja Subodh Chandra Mallick Road, Jadavpur, Kolkata, West Bengal 700032 India

**Keywords:** Cd(II) bioremediation, *Candida tropicalis* XTA 1874, Biosorption, Adsorption isotherm, Desorption, Biochemistry, Biological techniques, Biotechnology, Chemical biology, Microbiology

## Abstract

Cd(II) is a potentially toxic heavy metal having carcinogenic activity. It is becoming widespread in the soil and groundwater by various natural and anthropological activities. This is inviting its immediate removal. The present study is aimed at developing a Cd(II) resistant strain isolated from contaminated water body and testing its potency in biological remediation of Cd(II) from aqueous environment. The developed resistant strain was characterized by SEM, FESEM, TEM, EDAX, FT-IR, Raman Spectral, XRD and XPS analysis. The results depict considerable morphological changes had taken place on the cell surface and interaction of Cd(II) with the surface exposed functional groups along with intracellular accumulation. Molecular contribution of critical cell wall component has been evaluated. The developed resistant strain had undergone Cd(II) biosorption study by employing adsorption isotherms and kinetic modeling. Langmuir model best fitted the Cd(II) biosorption data compared to the Freundlich one. Cd(II) biosorption by the strain followed a pseudo second order kinetics. The physical parameters affecting biosorption were also optimized by employing response surface methodology using central composite design. The results depict remarkable removal capacity 75.682 ± 0.002% of Cd(II) by the developed resistant strain from contaminated aqueous medium using 500 ppm of Cd(II). Quantitatively, biosorption for Cd(II) by the newly developed resistant strain has been increased significantly (*p* < 0.0001) from 4.36 ppm (non-resistant strain) to 378.41 ppm (resistant strain). It has also shown quite effective desorption capacity 87.527 ± 0.023% at the first desorption cycle and can be reused effectively as a successful Cd(II) desorbent up to five cycles. The results suggest that the strain has considerable withstanding capacity of Cd(II) stress and can be employed effectively in the Cd(II) bioremediation from wastewater.

## Introduction

Heavy metals have a profound distribution in the environment. They are being classed as both essential and non-essential elements^1^. The term ‘Heavy Metal’ defines the group of metals and metalloids having atomic density greater than $5\;{\text{g }}\;{\text{cm}}^{ - 3}$^2^. The transition metals Copper (Cu), Chromium (Cr), Cobalt (Co), iron (Fe), Mercury (Hg), Cadmium (Cd), Lead (Pb), Molybdenum (Mo), Nickel (Ni), Strontium (Sr), Zinc (Zn), Vanadium (Vi) and Titanium (Ti), the metalloids such as Boron (Bo) and Arsenic (As) have been included in this definition^3–5^. The heavy metals regarded as essential act as coenzymes in metabolic reactions whereas the nonessential ones are not as potentially toxic at lower concentrations. They invite various health hazards and spare none of the organs in our body inducing renal, hematopoietic, cardiovascular, neurological, respiratory, gastro-intestinal and reproductive anomalies^6^. Soil fertility gets shattered by disrupting the balance in soil microbial community along with imposing adverse impact on other animals owing to intervention of these noxious elements^7^. The worth mentioning of such metals are cadmium, chromium, zinc, mercury, copper, lead and arsenic. Industrialization and technological advancements has invited unscrupulous release of heavy metals in the environment^8^. The industrial effluents contain considerable amount of heavy metals and other pollutants^9^. After entering rivers and other water bodies these waste waters cause serious damage of water resources.

Among all the toxic metals discussed above, cadmium exhibits very high toxicity even in trace amounts^10^. It spares no organs of human body and especially affects the kidney, liver and lung and above all it is a potent carcinogen^5^. Cadmium was discovered as an impurity in calamine (ZnS) by two german scientists Friedrich Stromeyer (1776–1835) and Karl Samuel Leberecht Hermann (1765–1846)^11,12^. Cadmium is the 67th most abundant element in the earth’s crust and present in the range of 0.1–0.5 ${\mu g}/{\text{g}}$^13^. Cadmium gets emited in the atmosphere from natural activities such biomas burning and volcanic activities^14^. The most vital anthropogenic sources of Cd(II) release are mining, smelting, Ni–Cd(II) batteries, metal plating, steel anticorrosives, plastic stabilizers, solar cells, pigments, disposal of sewage sludges, cigerrette somking, phosphate fertilizers and manures^15,16^. Such varied usage make it almost ubiquitous pollutant in soil, air and groundwater. Accumulation of soluble cadmium (Cd^2+^) is quite prevalent in some plants such as rice, tobacco and mushrooms and thus efficiently disseminated throughout the food chain. In spite of its abundance no biological role has been documented for cadmium except some marine diatoms as a carbonic anhydrase Ca^2+^ replacement^17^. Being a soft acid in HSAB concept Cd(II) has a high affinity for sulphur containing soft base ligands such as metallothionein and cysteine enriched proteins^14^. These proteins neutralise Cd(II) toxicity primarily by complexation. Competition for binding sites in metalloproteins is quite evident for Cd(II) and other divalent cations [Zn(II), Ca(II), Fe(II), Mn(II), Ni(II) and Cu(II)]^18,19^. Cd(II) having reached the liver combines with metallothionein and being transported through the hepatic portal circulation to kidney. There in the glumeroulus the ionic cadmium [Cd^2+^ or Cd(II)] mediates its toxic action^5,20^. Thus kindey is major site of Cd(II) toxic action and the other organs also gets the toxic slashes of Cd(II) are the lung, reproductive and endocrine and to a lesser extent on the nervous system. The prime molecular events of this toxic action is generation of oxidative stress, DNA damage by dampening the repair machinery as well and ultimately apoptosis or otherwise to carcinogenesis^5^.

The permissible limit of Cd(II) in drinking water is 0.003 ppm^21^. Owing to illegal release of toxic sludges from industrial, municipal runoff accumulation of Cd(II) is increasing in waterbodies in India^22,23^. It is an alarming threat towards human civilization. Water bodies in various regions in India, such as Uttar Pradesh, Punjab and southern domain of India already bear Cd(II) concentration higher than the permissible limit^22,24,25^. Cadmium laden impurites from the zinc galvanized pipes are the main reason for contamination in drinking water with cadmium^26^. Distribution of cadmium in soil and groundwater and its worldwide status is quite well deciphered in the review article^27^.

The physico-chemical methods employed in the removal of Cd(II) and other contaminants suffer from various drawbacks. Costly process operation, incomplete metal ion removal, large amounts of reagent requirements and generation of toxic sludge requring additional removal costs are the prime shortcomings of the conventional physico-chemical remediation techniques^10,28–30^. Adsorptive removal by nanomaterials such as graphene, carbon nanotubes and other non-toxic nanoadsorbents are quite omnipresent in removing heavy metals from wate water with high efficiencies^31^. Bioflocculants, a complex mixture of high molecular weight polymers released by microbes, have garnered substantial attention from the scientific community and the global community in recent decades. Their biodegradable nature, absence of hazards, and ability to prevent secondary contamination have made them particularly useful in the treatment of wastewater^32^. The effectiveness of various assorbents over conventional physico-chemical methods of remediation methods have been explained in detail as well as the supiroorrity of microbial adosrbents over others have also been focussed^33^.

Biological remediation techniques employing microbial biomass and other living materials provide a cheap, eco-friendly and efficient manner of environmental remediation. Microorganisms owing to their high surface area to volume ratio show tremendous efficacy in the removal of Cd(II) by surface adsorption. Bacterial, algal and fungal biomass have shown tremendous potency in the removal of Cd(II) from wastewater^34–52^. It has been evidenced that metal tolerant strains show better biosorption efficiencies compared to the non resistant ones when living biomass are being used^53^. Other biomaterials of phytological and zoological origin such as whole plant biomass, grape pomace, stem powder, rose wastes, calcined oyster shells, peat and fish scales have also acted as quite successful biosorbents^54–60^. Living microbial cells remove contaminants by both metabolism dependent and independent manner^61^. Fungal biosorbents are quite supirior biosorbents over other biosorbents owing to be cultivated at large scale using cheap and easy fermentation techniques^62^. Additionally, fungi are capable of decomposing a wide range of hazardous materials, including manganese (Mn), lead (Pb), cadmium (Cd), chromium (Cr) and arsenic (As). Fungi can remove these metals from soil since they are in their most basic form already and cannot be broken down any more. In terms of anatomy, ecology, and metabolism, fungi are well adapted to their surroundings. In natural environments, they carry out processes including decomposition and nutrient cycling^63^. In addition to being resilient in nature, the fungal strains utilized in mycoremediation can tolerate extremes in pH, nutritional conditions, and moisture content^64^.

Monitoring the increment in biosorption capacity $(\%)$ by empirical optimization is time consuming, laborious and costly. These drawbacks can be overcome by using response surface methodology. It decreases the number of tests and aids in studying the impact of individual and reciprocal interactions between the factors and the response^51^.

The primary objective of the present study is to develop a Cd(II) resistant yeast strain isolated from wastewater body by gradually adapting to Cd(II) stress with subsequent evaluation of its Cd(II) biosorption efficiency. Effects of eight physical parameters such as pH, Temperature °C$$)$$, Age of inoculum $$\left( h \right)$$, Volume of medium $$\left( {{\text{mL}}} \right)$$, Volume of inoculation $$\left( {8 \times 10^{6} \;{\text{cells}}/{\text{mL}}} \right)$$, Intial Cd(II) concentration $$\left( {{\text{ppm}}} \right)$$, Contact time $$\left( {{\text{min}}} \right)$$ and Dry cell weight $$\left( {{\text{mg}}/{\text{mL}}} \right)$$ on the biosorption capacity by the developed resistant strain have also been optimized by response surface methodology using Central-Composite Design. Microscopic, spectroscopic and diffractometric studies have shown its interaction with Cd(II) and morphological variation in the due course of adaptation. Contribution of specific cell wall component in the interaction with Cd(II) has been evaluated. Equilibrium adsorption data were analyzed using isotherm and kinetic modelling. Thermodynamic and kinetic parameters such as $$\Delta {G}^{0}$$, $$\Delta {H}^{0}$$, $$\Delta {S}^{0}$$, $${E}_{a}$$,$$\Delta {G}^{\#}$$, $$\Delta {H}^{\#}$$ and $$\Delta {S}^{\#}$$ have been evaluated. Desorption and reusibility of the biomass has also been studied.

## Materials and methods

### Development of Cd(II) resistant strain

A yeast strain was isolated from wastewater of Tollygunge Canal and was identified phylogenetically as *Candida tropicalis* MZ798901^65^.The strain was proved to be non-pathogenic when treated on Swiss albino mice^65^. It was then further undergone a gradual adaptation process with increasing Cd(II) concentration in the YEPD (Yeast Extract Peptone Dextrose) medium. The process was continued until the minimal inhibitory concentration (MIC) was reached and the developed strain capable of coping with considerable Cd(II) stress was obtained^10,66^.

### Optimization of physical parameters influencing Cd(II) biosorption by *Candida* tropicalis XTA 1874 using response surface methodology (RSM)

#### Experimentation and optimization of Cd(II) biosorption

The Design Expert Software (DOE, version 13, Stat-Ease Inc, Minneapolis, MN, USA) has been used to fit quadratic model to the experimental data and in order to determine the best combination of parameters that gave in the optimum response value. Optimization of Cd(II) biosorption by *Candida tropicalis* XTA 1874 was determined by Central Composite Design (CCD) under Response Surface Methodology (RSM). RSM constitutes a group of empirical techniques evaluating the relationship between clusters of independent variables and the measured responses. Since empirically determining the effects of single factors at a time is time consuming, RSM boosts up the operational conditions as well as save the economy of the process by reducing experimental runs^67^. The latest study depicts the impact of physico-chemical parameters influencing the growth and metal bioremediation efficiency (%) of the resistant strain *Candida tropicalis* XTA 1874. Eight independent variables for the current study were: pH, temperature (°C), age of inoculum $(h)$, volume of medium $\left( {{\text{mL}}} \right)$, volume of inoculation ($8 \times 10^{6} \;{\text{cells}}/{\text{mL}}$), initial Cd(II) concentration $\left( {{\text{ppm}}} \right)$, contact time $\left( {{\text{min}}} \right)$ and dry cell weight $\left( {{\text{mg}}/{\text{mL}}} \right)$.The independent variables have been coded by following the equation:

1 $$ Z = \frac{{Z_{0}  - Z_{c} }}{{\Delta Z}} $$

where $Z$ and ${Z}_{0}$ denote the coded and real levels of independent variables. The step change is indicated by $\Delta Z$ and the real value at the center point is indicated by ${Z}_{c}$ .The interaction among the independent variables and the response was determined by the quadratic equation mentioned below:

2 $$Y = \upbeta _{0}  + \sum _{{i = 1}}^{k} \upbeta _{i} X_{i}  + \sum _{{i = 1}}^{k} \upbeta _{{{\text{ii}}}} X_{i}^{2}  + \sum _{{i = 1}}^{{k - 1}} \sum\limits_{{{\text{j}} = 1}}^{{\text{k}}} {\upbeta _{{{\text{ij}}}} }\, X_{{\text{i}}} X_{{\text{j}}}  + \epsilon$$

where ${X}_{i}$, ${\text{X}}_{\text{j}}$,…${\text{X}}_{\text{k}}$ denote the linear, ${{X}_{i}}^{2}$, ${{X}_{j}}^{2}$,…${{X}_{k}}^{2}$ quadratic and the ${X}_{i}{X}_{j}$, ${X}_{i}{X}_{k}$ and ${X}_{j}{X}_{k}$ are interaction effects of the variables on the predicted response $Y$ respectively. The terms ${\beta }_{0}$, ${\upbeta }_{\text{i}}$ $(i=\text{1,2}\dots k$), ${\upbeta }_{\text{ii}}$ $(i=\text{1,2}\dots k)$, and ${\upbeta }_{\text{ij}}$ $(i=\text{1,2}\dots k)$ are the intercept term, linear, quadratic and interaction terms respectively, the random error is $\upepsilon$ and the predicted response are indicated by $Y$^68–70^. The experiment was carried out in 282 runs having centre points 10 and non-centre points 272 with each factor being evaluated at five levels having a star low ($-\alpha$) low ($-1$), center ($0$), high ($+1$) and star high ($+\alpha$). The optimum values of the selected variables were obtained by solving the regression equation and analyzing the response surface plots as well.

### Morphological and elemental analyses

#### Scanning *electron* microscopic studies of enzyme untreated cells

Scanning electron microscopic studies were carried out in order to elucidate the changes in surface morphology of the developed strain compared with the control strain under Cd(II) untreated conditions. Software controlled scanning electron microscope (Quorum Q150T ES) was used for this purpose. Dried biomass of the strains were applied to graphite stickers and placed on aluminium table. The preparations were sputter coated with platimum under vacuum in order to enhance electron conductivity and to improve micrograph quality. Energy dispersive X-ray analyses were carried out for Cd(II) treated and untreated resistant strain compared with the control to elucidate the elemental composition of the samples. Elemental composition of both the live cells of Cd(II) treated and untreated resistant strain along with untreated control were carried out by EDAX microanalyzer (Zeiss SmartEDX) conjugated with the Scanning Electron Microscope (SEM) (Quorum Q150T ES) to find out whether Cd(II) adsorption occurs on the resistant strain surface after Cd(II) treatment.

#### Enzymatic treatment of the biomass

$0.5\;{\text{g}}$ Wet biomass of the developed resistant strain *Candida tropicalis* XTA 1874 was suspended at 30 °C by a pre-treatment agent for $30 \;{\text{min}}$^71^. It was then washed twice with hypertonic buffer $\left( {7000\; {\text{rpm}}, \;5\; {\text{min}}} \right)$. The biomass was then undergone enzymatic treatment with $2 \%$ β-mannanase, $2 \%$ proteinase K, dual treatment of both enzymes and $2 \%$ snailase. The reaction was allowed to carry out for $6 \;{\text{h}}$. The biomass was then separated by centrifugation at $7000 \;{\text{rpm}}$ for $10\; {\text{min}}$ with subsequent studies.

#### Scanning *electron* microscopic studies of enzyme treated cells

The enzyme treated cells along with untreated control were subjected for another scanning electron microscopic study (Quorum Q150T ES) after treating the resistant strain with Cd(II). The dried biomass of the strains were treated with gluteraldehyde (Sigma–Aldrich) and dehydrated by ethanol treatment $(30-100 \%)$^72^. The samples were then observed under the Scanning Electron Microscope (Quorum Q150T ES) after being sputter coated by platinum. Energy dispersive X-ray analyses were carried out again for Cd(II) treated biomass undergone enzymatic treatment by EDAX microanalyzer (Zeiss SmartEDX) conjugated with the Scanning Electron Microscope (SEM) (Quorum Q150T ES) to find out the critical cell wall component involved in cellular interaction with Cd(II).

### Spectroscopic and diffractometric analyses

#### FT-IR, Raman, EDAX, XPS and XRD analyses

FT-IR spectroscopy analyzes the electromagnetic radiation absorbed by the sample. The yeast biomass of both Cd(II) treated and untreated sample was centrifuged $\left( {10000 \;{\text{rpm}}, \;10{\text{ min}}, \; + 4 \;^{ \circ } {\text{C}}} \right)$ using Cooling Centrifuge, Remi c24BL and separated from the broth culture. The cell pellets of the biomass of both Cd (II) treated and untreated resistant strain were washed thrice with deionized water to remove the growth media residuals. After having lyophilized both the Cd(II) treated and untreated biomass of the resistant strain and the untreated control mother isolate were subjected to FT-IR spectroscopic analysis in the range of 400–4000 cm^−1^ using FT-IR spectrophotometer (Perkin Elmer, Spectrum 100) equipped with beam splitter (KBr) and DTGS (deuterated triglycine sulphate) detector. The results were confirmed by Raman Spectral Analysis $\left( {100 - 3000 \;{\text{cm}}^{ - 1} } \right)$ using Raman Spectrometer (Horiba Instruments, USA 1024X256-OE).

XPS (K-Alpha^+^, Thermo Fisher Scientific, USA) analysis was carried out before and after Cd(II) treatment of the resistant strain as well as with the untreated control mother strain. X-ray diffraction analysis was also carried out using Shimadzu X-ray diffractometer XRD 6000. It employed Cu-Kα radiation to generate diffraction patterns from the powdered samples of the Cd(II) treated and untreated developed resistant strain and untreated control strain.

### Mathematical models

#### Equilibrium biosorption studies for Cd(II)

Equilibrium biosorption experiments by the developed resistant strain were carried out using Cd(II) containing YEPD (Yeast Extract, $0.3\%$; Peptone, $0.3\%$; Glucose, $2\%$) broth solutions using Cd(II) chloride (CdCl_2_.H_2_O, NICE) prepared in distilled water^51,65^. The Cd(II) concentration in the solution ranged from $15 - 500 \;{\text{ppm}}$. Biosorption experiments were carried out by adding dry biomass $\left( {0.15\; {\text{g}}} \right)$ in $100 \;{\text{mL}}$ broth medium. The pH and temperature of the solution was maintained at $6.3$ and $27 \pm 2\;^{ \circ } {\text{C}}$ under shaking conditions at $180 \;{\text{rpm}}$ on a rotary shaker (REMI, RS-12R) respectively. After various time intervals the biomass was separated by centrifugation using cold centrifuge (Remi c24BL). Cd(II) content in the supernatant was detected by flame atomic absorption spectroscopy (Shimadzu AA-7000, Japan). Exponential phase cultures $\left( {455 \times 10^{4} \;{\text{cells}}/{\text{mL}} \;{\text{at}} OD_{600} 0.15} \right)$ were used in order to carry out the biosorption study at ambient temperatures $\left( {27 \pm 2 \;^{ \circ } {\text{C}}} \right)$^65^.

The amount of Cd(II) removal per unit mass of the biosorbent at equilibrium conditions was calculated by the following equation:

3 $${q}_{e} =\frac{\left({C}_{0}-{C}_{e}\right)V}{m}$$

${q}_{e}$ signifies the amount of Cd(II) uptake (${\text{mg}}$ of Cd(II) ${\text{removed}}/{\text{g}}$ dry weight) at equilibrium, $V$ signifies the sample volume $\left( {{\text{mL}}} \right)$, $C_{0}$ is the concentration $\left( {{\text{ppm}}} \right)$ of Cd(II) initially added to the medium and $C_{e}$ is the final Cd(II) concentration $\left( {{\text{ppm}}} \right)$ remained in the medium after equilibrium and $m$ is the weight in dry mass of the biosorbent $\left( g \right)$.

The percent Cd(II) removed at equilibrium by biosorption was calculated by the equation:

4 $$\% {\text{ Cd}}\left( {II} \right)removal = \frac{{C_{0} - C_{e} }}{{C_{0} }} \times 100$$

${C}_{0}$ signifies the initial Cd(II) concentration $(\text{ppm})$, ${C}_{e}$ signifies the Cd(II) concentration $(\text{ppm})$ in the supernatant after centrifugation at equilibrium. The percent removal can also be considered as biosorption efficiency^73^.

Both the amount of surface adsorbed and intracellularly mobilized Cd(II) concentration have been studied in the due course of the study. The biomass obtained from the study was washed thrice with deionized water and was subsequently undergone treatment with $0.1\;{\text{M}}$ EDTA solution for $10 \;{\text{min}}$. Surface adsorbed Cd(II) was determined at the EDTA washable fraction^51^. The intracellular fraction was determined by acid digestion ($0.2 \;{\text{N}}$ H_2_SO_4_ and HNO_3_) and Cd(II) content in the lysate was estimated by Flame Atomic Absorption Spectroscopy (Shimadzu AA-7000, Japan) at $228.8 \;{\text{nm}}$^51^.

The equilibrium adsorption capacity of Cd(II) was calculated by employing the Langmuir and Freundlich isotherms.

Langmuir model describes the formation of monolayer by the adsorbate molecules over the adsorbent surface and assumes continuous energy for adsorption regardless the degree of coverage. It is expressed as the equation as:

5 $${q}_{e}= {q}_{max}\frac{{K}_{l}{C}_{e}}{1+ {K}_{l}{C}_{e}}$$

The linearized version of the equation is represented by:

6 $$\frac{1}{{q}_{e}}=\frac{1}{{q}_{max}}+\frac{1}{{q}_{max}{\times K}_{l}}\times \frac{1}{{C}_{e}}$$

The values of ${q}_{max}$ and ${K}_{l}$ were determined based on the linear dependence of $\frac{1}{{q}_{e}}$ on $\frac{1}{{C}_{e}}$.The term ${q}_{max}$ is defined as maximum adsorption capacity (${\text{mgg}}_{d.w.}^{ - 1}$_)_ and ${K}_{l}$ is the Langmuir constant (${\text{Lmg}}^{ - 1}$). From Langmuir equation the value of a dimensionless constant called ${R}_{l}$ was calculated by the following formula^74^:

7 $${R}_{l} =\frac{1}{1+ {K}_{l}{C}_{0}}$$

The Freundlich isotherm implies multilayer adsorption on heterogeneous surfaces and takes into account the interactions between the molecules^74–76^. The equation is represented below:

8 $${q}_{e} = {K}_{f} {C}_{e}^\frac{1}{n}$$

The straight line fitting gives the equation

9 $$log {q}_{e} = log {K}_{f} +\frac{1}{n} log {C}_{e}$$

${K}_{f}$ is the Freundich constant characterizing maximum adsorption capacity (${\text{Lmg}}^{ - 1}$). The term $n$ determines the characteristics of adsorption. Both ${K}_{f}$ and $n$ were determined form the linear plot of $log {q}_{e}$ versus $log {C}_{e}$.

#### Biosorption kinetics

Among the kinetic models described in the literature those that express the order of chemical reactions are highly considered, especially the pseudo first order (Lagergren) and pseudo second order (Ho and McKay) models^77,78^. Pseudo first order kinetic model has been applied by using the linearized equation

10 $$\ln (q_{e} - q_{t} ) = \ln \;q_{e} - k_{1} t$$

The values of ${q}_{e}$ and ${k}_{1}$ parameters were obtained by linear regression method employing the plots of $ln ({q}_{e}-{q}_{t})$ versus $t$

Pseudo second order kinetic modelling has been applied by using the linearized form

11 $$\frac{t}{{q}_{t}}=\frac{t}{{q}_{e}}+\frac{1}{{k}_{2}{q}_{e}^{2}}$$

The pseudo second order parameters ${q}_{e}$ and ${k}_{2}$ were determined by plotting $\frac{t}{{q}_{t}}$ against $t$.

In the due course of the equilibrium biosorption studies by applying adsorption isotherms and kinetic analysis the amount of Cd(II) adsorbed on the surface was determined by using $0.1\;{\text{M}}$ EDTA for $10min$ after washing thrice with deionized water. The amount of Cd(II) was then measured in the supernatant by flame atomic absorption spectroscopy (Shimadzu AA-7000, Japan)^25^. Intracellular accumulation of Cd(II) was determined by acid digestion ($0.2 \;{\text{N}}$ H_2_SO_4_ and HNO_3_) of the biomass and measuring the Cd(II) in the lysate by Flame Atomic Absorption Spectroscopy (Shimadzu AA-7000, Japan) at $228.8 \;{\text{nm}}$^67^.

### Desorption experiment

In the due course of studying Cd(II) adsorption it was quintessential to investigate the desorption capacity and the reusability of the biosorbent. The biomass $\left( {0.15 \;{\text{g}}} \right)$ was sequestered from the adsorbing solution and washed thrice with deionized water. It was then re-suspended in the eluent solution $\left( {0.2 \;{\text{M}} \;{\text{HCl}}} \right)$ and agitated for $2.5\;{\text{ h}}$. Cd(II) concentration in the liquid phase was measured by Atomic Absorption Spectroscopy (Shimadzu AA-7000, Japan)^51^. The desorption efficiency ($\eta \%$) was calculated from the following equation:

12 $$\%\upeta =\frac{{M}_{desorbed}}{ {M}_{adsorbed}}\times 100\%$$

13 $$=\frac{{C}_{r}{V}_{r}}{\left({C}_{i}-{C}_{e}\right)V}\times 100\%$$

$M_{desorbed}$ represents amount of Cd(II) desorbed $\left( {{\text{mgg}}^{ - 1} } \right)$ and $M_{adsorbed}$ $\left( {{\text{mgg}}^{ - 1} } \right)$ as amount adsorbed with the biomass. The terms $V_{r}$ and $C_{r}$ represents desorption volume $\left( {\text{L}} \right)$ and concentration of Cd(II) in the desorption solution^51^.

Field emission scanning electron microscopic studies were carried out in order to elucidate the changes in surface morphology of the developed strain after desorption. Software controlled field emission scanning electron microscope (FE SEM) (QUANTA FEG 250) was used for this purpose. Dried biomass of the strains were applied to graphite stickers and placed on aluminium table. The preparations were sputter coated with gold under vacuum in order to enhance electron conductivity and to improve micrograph quality. Elemental analysis by EDAX (EDAX APEX) was also carried out to elucidate whether any trace amount of Cd(II) still retained on the biomass. FT-IR analysis $\left( {400 - 4000 \;{\text{cm}}^{ - 1} } \right)$ was also carried out again for each six cycles to investigate the interaction of the surface exposed functional groups with the residual amount of bound Cd(II) on the biomass after desorption. FT-IR spectrophotometer (Perkin Elmer, Spectrum 100) equipped with beam splitter (KBr) and DTGS (deuterated triglycine sulphate) detector was used for the purpose.

### Statistical analysis

Student’s t-test was performed to find out whether or not there is significant increase in biosorption capacity of Cd(II) by the resistant strain compared to the non-resistant strain.

## Results and discussion

### Emergence of Cd(II) resistant strain

*Candida tropicalis* MZ798901 strain isolated from wastewater (Tollygunge Canal) reached its minimal inhibitory concentration (MIC) at $3850 \;{\text{ppm}}$ of Cd(II). The developed strain was subsequently named as *Candida tropicalis* XTA 1874. So far as the trend in Cd(II) resistant strain development is concerned especially using *Candida tropicalis* strains, maximum tolerance level reported to be up to $2800\; {\text{ppm}}$ of Cd(II)^10,79^. The strain we have developed has shown more resistance to Cd(II) compared to the former ones.

### Response surface analysis

#### Central composite design (CCD) and statistical analysis

Response Surface Methodology (RSM) was successfully applied to identify the significant parameters influenced Cd(II) biosorption efficiency $(\%)$ and to demonstrate the optimum conditions favoring maximal biosorption capacity by the strain *Candida tropicalis* XTA1874. The experimental design with names, symbol codes and actual variable levels has been shown in (Table 1). The quadratic regression model as a function of pH (A), temperature (B) age of inoculum (C), volume of medium (D), volume of inoculation (E), initial Cd(II) concentration (F), contact time (G) and dry cell weight (H) have been shown in (Table 2). The $F$ and $p$ values are considered to be important in determining the significance of each of the variables. The Model $F-value$ of $49.92$ implies the model is significant. There is only a $0.01\%$ chance that a $F-value$ this large could occur owing to noise. It has been confirmed by the regression analysis that the linear model terms (A), (H) and the quadratic terms (A^2^), (B^2^), (C^2^), (D^2^) and (H^2^) were significant $(p<0.05)$. The estimation of the quadratic model design matrix was done by using $p-values$. The lack of fit value for Cd(II) biosorption efficiency $(\%)$ was found to be not significant $(p>0.05)$. The lack of fit $F-value 0.52$ implies the lack of fit was not significant relative to the pure error. The estimation of $F-value$ was carried out by dividing model mean square by residual mean square comparing the model variance and residual^80^. The $\text{coefficient of variance} (CV)$ of $4.74\%$ ascertained the reliability and precision of experimental data. Moreover, the insignificant lack of fit and high determination coefficient (${R}^{2}= 0.9026$) agreed well with the adjusted ${R}^{2}$ ($Adj{R}^{2}= 0.8845$) implying the validity and fitness of the model. The adequate precision of $42.4513 (>4)$ showed the signal to noise ratio comparing the predicted values at the design points to the average prediction error^66,80^.

**Table 1:** Independent variables and their corresponding levels for Cd(II) biosorption.

Independent variables	Symbol	Coded levels				
		− α	− 1	0	+ 1	+ α
pH	A	3.97731	5	6.5	8	9.02269
Temperature (°C)	B	21.9546	24	27	30	32.0454
Age of inoculum (h)	C	7.63697	24	48	72	88.363
Volume of medium (mL)	D	15.9104	50	100	150	184.09
Volume of inoculation (8 × 10^6^ cells/mL)	E	2.31821	3	4	5	5.68179
Intial Cd(II) concentration (ppm)	F	331.821	400	668.179	600	668.179
Contact time (min)	G	65.9104	100	150	200	234.09
Dry cell weight (mg/mL)	H	0.659104	1	1.5	2	2.3409

**Table 2:** Regression analysis using central composite design (CCD).

Source	Sum of squares	df	Mean Square	$F-value$	$p-value$	
Model	13,688.41	44	311.10	49.92	< 0.0001	significant
A-pH	3729.33	1	3729.33	598.45	< 0.0001	
B-Temperature	6.50	1	6.50	1.04	0.3081	
C-Age of inoculum	1.69	1	1.69	0.2707	0.6034	
D-Volume of medium	0.5782	1	0.5782	0.0928	0.7609	
E-Volume of inoculation	4.43	1	4.43	0.7102	0.4002	
F-Initial Cd(II) concentration	3.80	1	3.80	0.6102	0.4355	
G-Contact time	0.6694	1	0.6694	0.1074	0.7434	
H-dry cell weight	140.16	1	140.16	22.49	< 0.0001	
AB	12.12	1	12.12	1.94	0.1645	
AC	5.95	1	5.95	0.9544	0.3296	
AD	0.0619	1	0.0619	0.0099	0.9207	
AE	1.35	1	1.35	0.2164	0.6422	
AF	1.563^E−06^	1	1.563^E−06^	2.507E−07	0.9996	
AG	0.0172	1	0.0172	0.0028	0.9581	
AH	9.74	1	9.74	1.56	0.2124	
BC	0.2500	1	0.2500	0.0401	0.8414	
BD	19.01	1	19.01	3.05	0.0820	
BE	0.2704	1	0.2704	0.0434	0.8352	
BF	0.0012	1	0.0012	0.0002	0.9888	
BG	3.78	1	3.78	0.6071	0.4367	
BH	10.11	1	10.11	1.62	0.2040	
CD	0.9120	1	0.9120	0.1464	0.7024	
CE	5.21	1	5.21	0.8360	0.3615	
CF	1.64	1	1.64	0.2639	0.6079	
CG	3.38	1	3.38	0.5418	0.4624	
CH	0.9025	1	0.9025	0.1448	0.7039	
DE	1.08	1	1.08	0.1736	0.6773	
DF	1.11	1	1.11	0.1786	0.6730	
DG	1.46	1	1.46	0.2340	0.6290	
DH	1.22	1	1.22	0.1959	0.6584	
EF	3.48	1	3.48	0.5582	0.4557	
EG	0.1892	1	0.1892	0.0304	0.8618	
EH	8.15	1	8.15	1.31	0.2539	
FG	3.44	1	3.44	0.5522	0.4582	
FH	1.27	1	1.27	0.2031	0.6526	
GH	2.62	1	2.62	0.4211	0.5170	
A^2^	2905.02	1	2905.02	466.17	< 0.0001	
B^2^	66.11	1	66.11	10.61	0.0013	
C^2^	63.58	1	63.58	10.20	0.0016	
D^2^	76.22	1	76.22	12.23	0.0006	
E^2^	15.50	1	15.50	2.49	0.1161	
F^2^	4.94	1	4.94	0.7923	0.3743	
G^2^	3.06	1	3.06	0.4915	0.4839	
H^2^	92.72	1	92.72	14.88	0.0001	
Residual	1476.91	237	6.23			
Lack of fit	1373.52	228	6.02	0.5244	0.9480	not significant
Pure error	103.39	9	11.49			
Cor total	15,165.32	281				

Fit Statistics						
Std. dev	2.50	R^2^	0.9026			
Mean	52.70	Adjusted R^2^	0.8845			
C.V. %	4.74	Predicted R^2^	0.8126			
		Adeq precision	42.4513			

**Figure 1:**
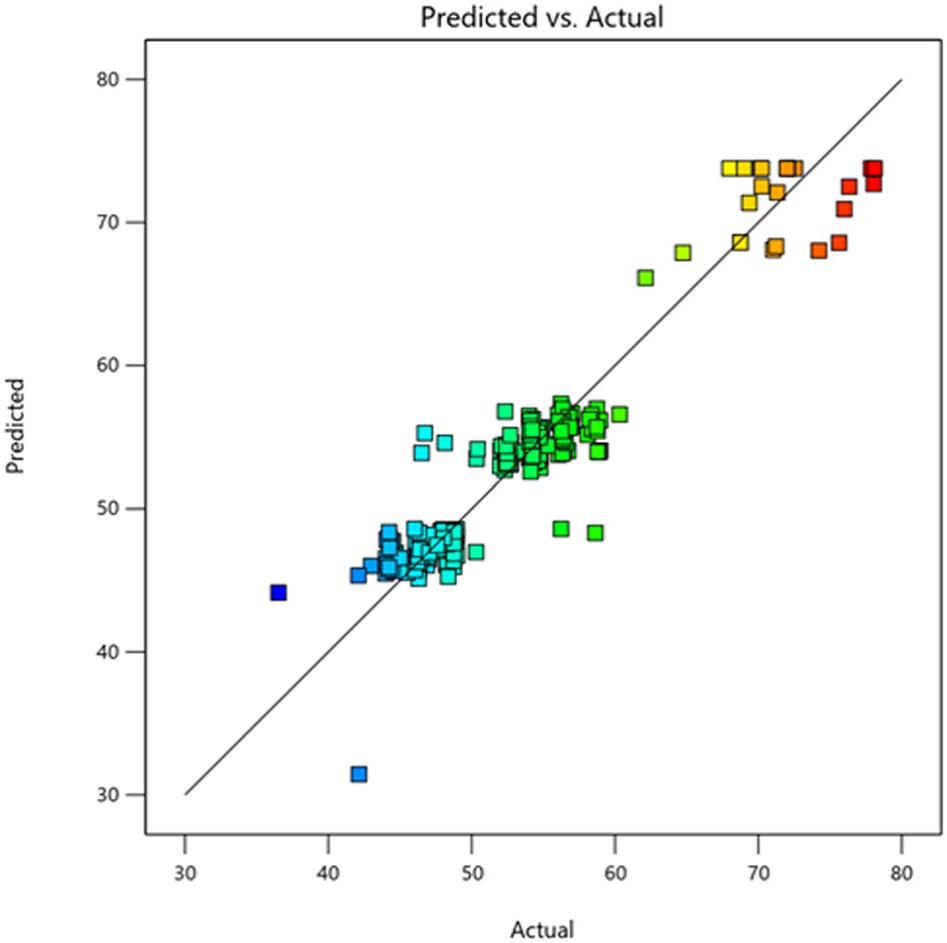
Comparison of Predicted versus Actual values for Cd(II) biosorption by live biomass of *Candida tropicalis* XTA 1874.

The ANOVA analysis resulted in a standard deviation of $2.50$ and a mean of $52.70$. Figure 1 showed that the actual and predicted values were very close to each other and distribution of the data was close to the fitted line. This indicated that the experimental model is suitable in describing the experimental data. According to the analysis the small probability value of the model was to confirm in rejecting the null hypothesis and the data followed a normal distribution. The equation obtained for the response variable has been shown in eq. (14).

14 $$\begin{aligned} Y_{{Cd\left( {II} \right)}} & = + 73.78 - 3.78A - 0.1576B - 0.0803C + 0.04705D + 0.1301E \\ & \quad + 0.1205F - 0.0506G + 0.7319H - 0.2176AB + 0.1524AC - 0.0155AD \\ & \quad + 0.0726AE + 0.0001AF + 0.0082AG - 0.1951AH - 0.0312BC - 0.2725BD \\ & \quad + 0.0325BE - 0.0022BF - 0.1216BG + 0.1988BH - 0.0597CD + 0.1427CE \\ & \quad + 0.0802CF + 0.1148CG - 0.0594CH + 0.0650DE - 0.0659DF - 0.0755DG \\ & \quad - 0.0691DH - 0.1166EF - 0.0272EG - 0.1784EH + 0.1159FG + 0.0703FH \\ & \quad - 0.1012GH - 12.72A^{2} - 1.92B^{2} - 1.88 C^{2} - 2.06 D^{2} - 0.9293 E^{2} - 0.5245 F^{2} \\ & \quad - 0.4131 G^{2} - 2.27 H^{2} \\ \end{aligned}$$

#### Interaction effects of the variables on Cd(II) biosorption efficiency (%) by the strain and selection of optimized conditions

**Figure 2:**
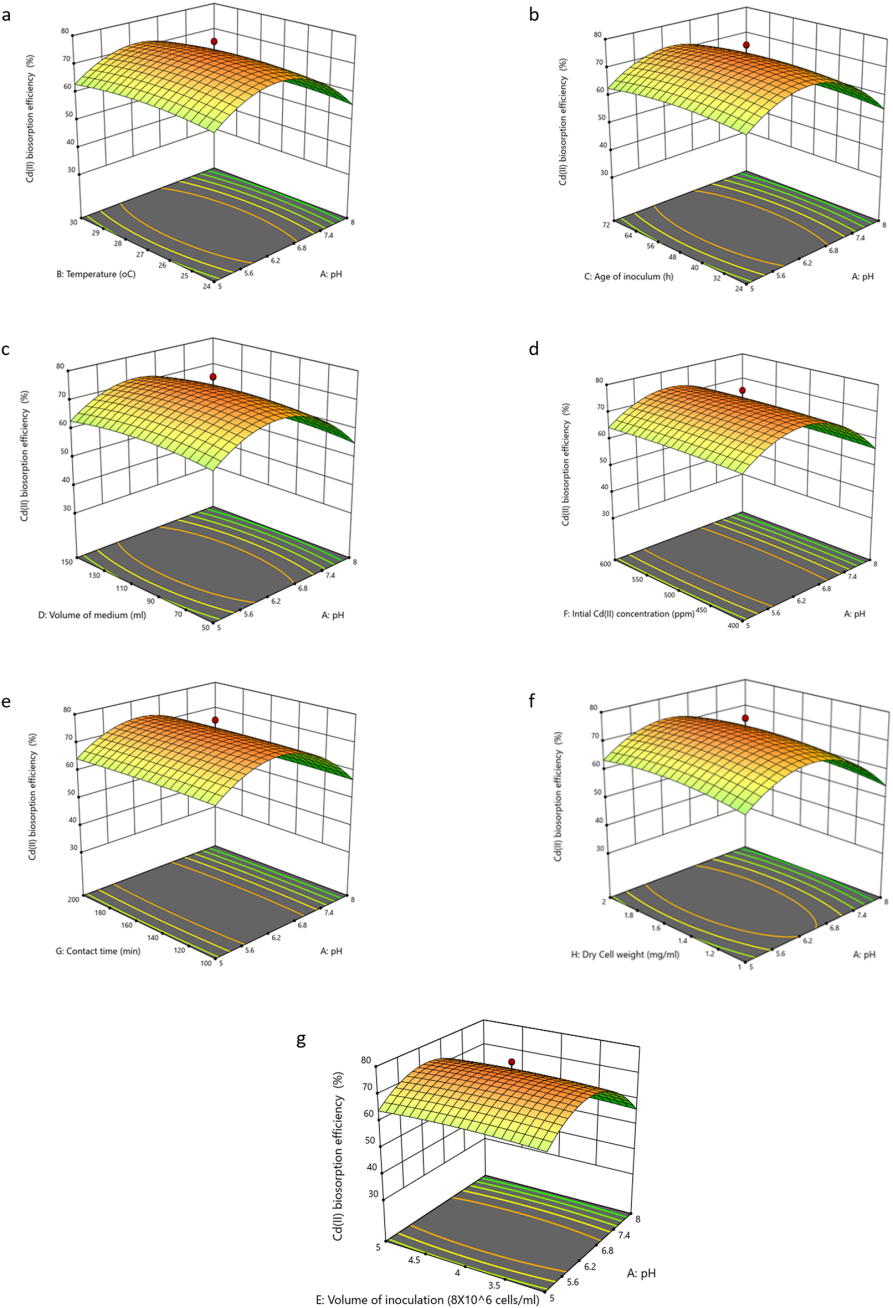
3D Response Surface Plots for the effects of (**a**) pH and Temperature (℃) (**b**) pH and Age of inoculum (h) (**c**) pH and Volume of medium (mL) (**d**) pH and Initial Cd(II) concentration (ppm) (**e**) pH and Contact time (min) (**f**) pH and Dry cell weight (mg/mL) (**g**) pH and Volume of inoculation (8 × 10^6^ cells/mL) on Cd(II) biosorption efficiency(%) by the live biomass of *Candida tropicalis* XTA 1874.

The physico-chemical parameters played significant roles in the growth and removal capacities by the organisms apart from the physical parameters. The contour plots showed that pH and dry cell mass had much significant effects governing the Cd(II) biosorption efficiency $(\%)$ by the strain. The results showed maximum Cd(II) removal $\left( {75.682 \pm 0.002\% } \right)$ was achieved under the optimum physico-chemical conditions: pH $(6.279)$, temperature $\left( {26.941 \;^{ \circ } {\text{C}}} \right)$, age of inoculum $\left( {47.505\; {\text{h}}} \right)$, volume of medium $\left( {100.659\; {\text{mL}}} \right)$, volume of inoculation $\left( {4.096 \;{\text{mL}}} \right)$, initial Cd(II) concentration $\left( {501 \;{\text{ppm}}} \right)$, contact time $\left( {149.853\; {\text{min}}} \right)$ and dry cell weight $\left( {1.585 \;{\text{mg}}/{\text{mL}}} \right)$ (Table 3). From the contour and 3D plots (Fig. 2), it was evident that Cd(II) biosorption efficiency $\left( \% \right)$ significantly increased with increasing pH, temperature of the medium with increasing dry cell mass. The parameter *pH* had profound influence in regulating the ionization properties of the functional groups on the adsorbent surface and solution chemistry of the metal ion^81^. After the optimum *pH*, biosorption capacity decreased a while because not surface adsorption but precipitation of metal hydroxides plays the predominant role^82^. Inoculum volume which influenced dry cell mass also had keen effect on the process but beyond the optimum value biosorption decreased. The glaring cause may be overcrowding of the adsorbate binding site in the adsorbent^72^. Effect of temperature was well deciphered in the due course of discussing the thermodynamics of the process.

**Table 3:** Experimental and predicted values of Cd(II) biosorption (%) at optimized conditions [Mean ± SEM, Sample size (n) = 6].

Optimum conditions	Coded levels	Actual levels
pH	− 0.0736	6.279
Temperature (°C)	− 0.0196	26.941
Age of inoculum (h)	− 0.0206	47.505
Volume of medium (mL)	0.0132	100.659
Volume of inoculation (8 × 10^6^ cells/mL)	0.096	4.096
Intial Cd(II) concentration (ppm)	− 0.6234	501
Contact time (min)	− 0.00294	149.853
Dry cell weight (mg/mL)	0.17	1.585
Response	Predicted values	Experimental values
Cd(II) biosorption (%)	74.129	75.682 ± 0.002

#### Validation of the model

The authors maintained the optimized conditions in order to check the fitting of the model for predicting the response value. Optimized Cd(II) biosorption was validated under optimized experimental conditions. The response value at optimized nutritional conditions was $74.129\%$. On the other hand, experimental value under optimized conditions was $75.682 \pm 0.002\%$ using $500\; {\text{ppm}}$ of initial Cd(II) concentration. Experimental response value was well in agreement with the predicted response value (Table 3).

### Analysis of adsorption potential for Cd(II)

#### Microscopic evidences

**Figure 3:**
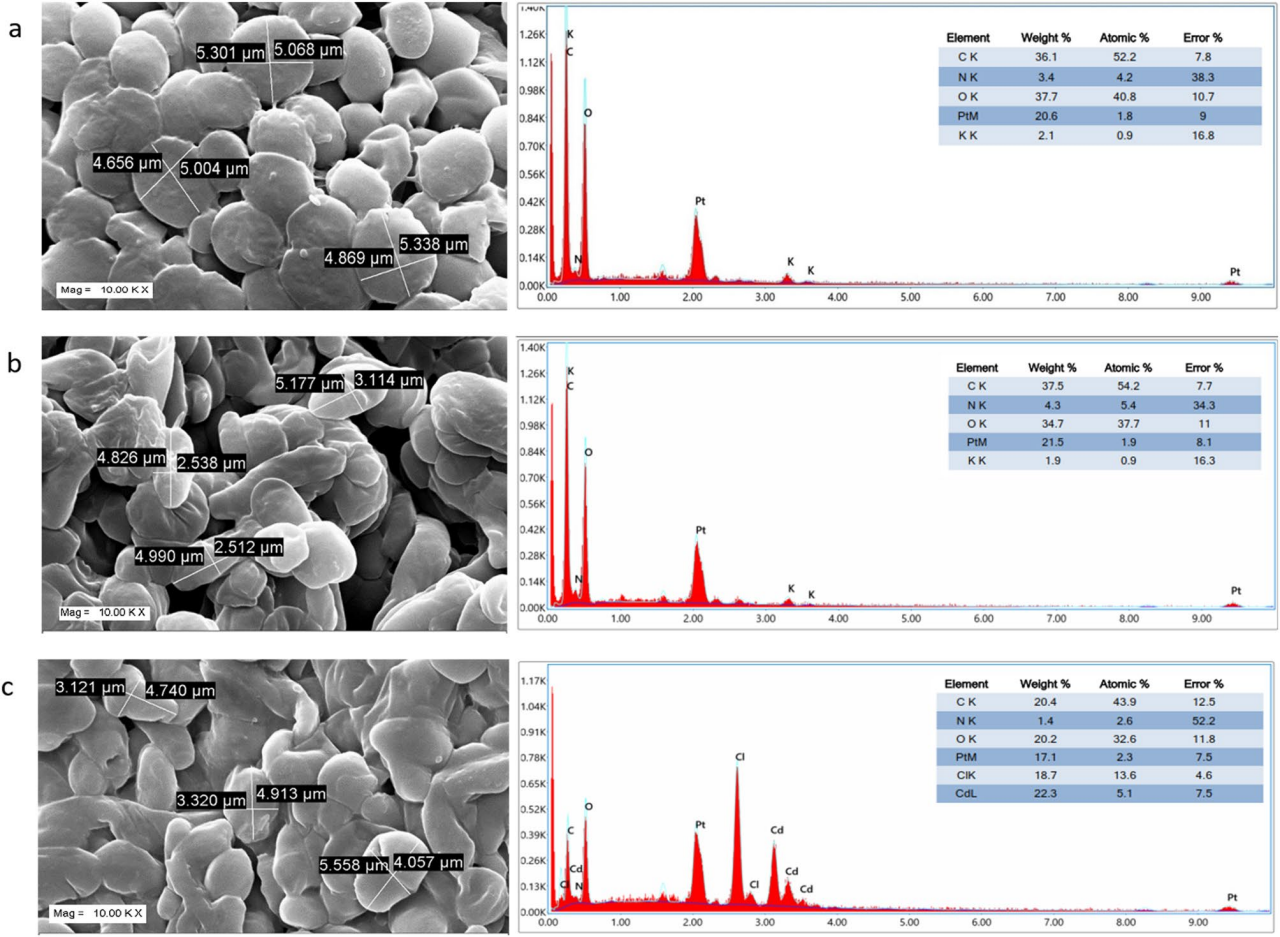
SEM imaging of Cd(II) untreated control isolate (**a**) Cd (II) untreated resistant (**b**) and treated resistant strain (**c**) *Candida tropicalis* XTA 1874.

Scanning electron micrographs were obtained from software controlled digital scanning electron microscope provided in Fig. 3. It is evident from the micrographs that the cells of the untreated isolate (control) retained its native yeast cell morphology retaining its ovoid structure $\left( {4.864 \pm 0.118\; {\mu m} \times 5.214 \pm 0.105\; {\mu m}} \right)$. But in the due course of adaptation in Cd(II) amended media considerable morphological changes have occurred in the cells of the developed resistant strain named *Candida tropicalis* XTA 1874 $\left( {4.919 \pm 0.299\; {\mu m} \times 2.698 \pm 0.291\;{\mu m}} \right)$. The cells have become considerably elongated quite prominent in the SEM image of the cells of the resistant strain. After treatment with Cd(II) considerable changes in cell shape have occurred compared to the resistant untreated strain $\left( {5.07 \pm 0.248 \;{\mu m} \times 3.499 \pm 0.285 \;{\mu m}} \right)$. The cells have adapted much more elongated shape after treatment with Cd(II) compared to the untreated cells of the developed resistant strain. Thus changes in cell shape were associated with increase in cell surface area which may aid in increased Cd(II) adsorption. Similar changes in surface morphology were obtained after treatment with Cd(II) in *Candida tropicalis* XTA 1874 under optimized conditions^51^ and after Se(IV) treatment in the cell surface morphology of a *Candida utilis* strain^74^. EDAX analysis (Fig. 3) provided elemental composition of the Cd(II) treated resistant strain compared with the untreated control. The elemental analysis showed the presence of C, N, O, K, Pt and Cl in the structure. The developed resistant strain, *Candida tropicalis* XTA 1874 which had undergone Cd(II) treatment shown the presence of Cd(II) in the EDAX spectra. The evidence amply demonstrated Cd(II) accumulation on the surface.

**Figure 4:**
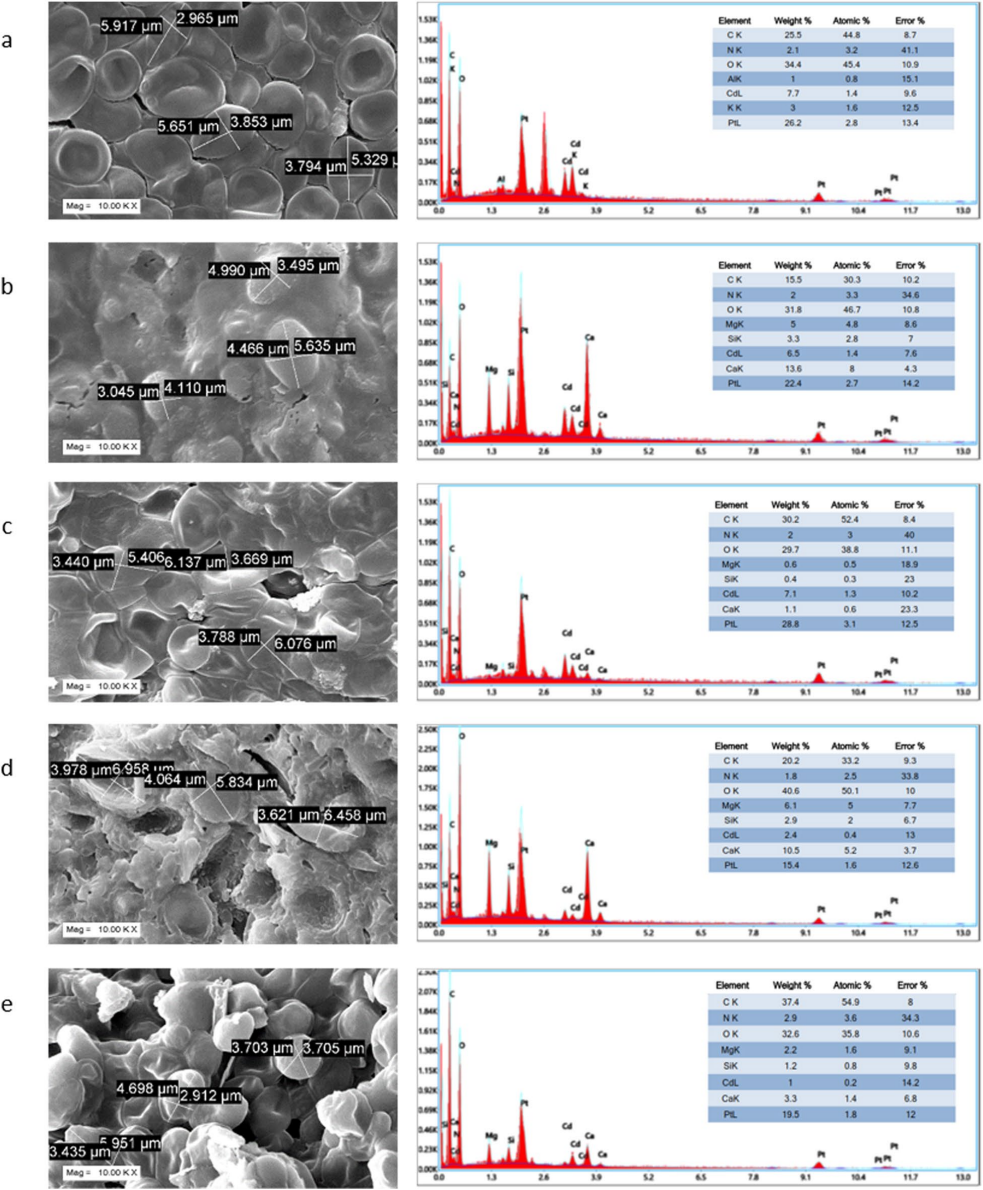
SEM imaging of enzyme untreated (**a**) β-Mannanase treated (**b**) Proteinase K treated (**c**) Both β-Mannanase and Proteinase K treated (**d**) Snailase treated (**e**) Cd(II) resistant strain *Candida tropicalis* XTA 1874.

**Figure 5:**
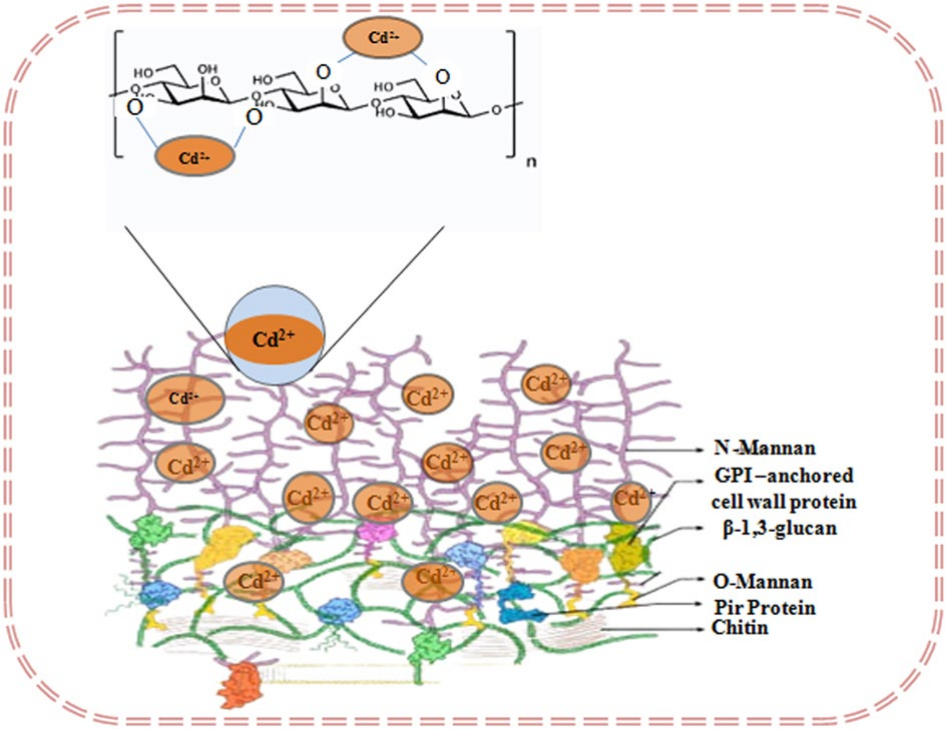
Hypothetical diagram depicting the role of cell wall Mannans in the interaction with Cd(II).

The micrographs of the enzyme untreated cells showed more or less native yeast cell surface morphology $\left( {5.632 \pm 0.169 \;{\mu m} \times 3.537 \pm 0.286 \;{\mu m}} \right)$ (Fig. 4). $\beta -$ Mannanase is an enzyme that degrades the mannan side chains of the cell wall mannoproteins. The cells being treated with the enzyme shows considerable changes in the surface morphology. Most of the cells have attained a more or less pleomorphic structure with disruption in the integrity of the cell wall. Some have still retained a distinct shape $\left( {4.912 \pm 0.441 \;{\mu m} \times 3.668 \pm 0.419\; {\mu m}} \right)$. Treatment with proteolytic enzyme such as Proteinase K didn’t impose any severe impact in cellular morphology like $\beta$-Mannanase. The cells retained its normal shape $\left( {5.873 \pm 0.234 \;{\mu m} \times 3.632 \pm 0.102 \;{\mu m}} \right)$. Dual treatment with both enzymes severely disrupted cellular morphology. The cells have attained a severe pleomorphic state. Some cells still have retained a distinct shape $\left( {6.416 \pm 0.234\; {\mu m} \times 3.632 \pm 0.102\; {\mu m}} \right)$. The cells when treated with an enzyme conjugate containing enzymes that remove the entire cell wall they have attained significant morphological changes too with dimension of $\left( {4.784 \pm 0.649 \;{\mu m} \times 3.35 \pm 0.232\; {\mu m}} \right)$. In the due course of the elemental analysis of the enzymatically treated cells of the strain surface adsorption of Cd(II) varied significantly compared to the untreated one. The $\beta$-mannanase treated strain showed the tremendous depletion of Cd(II) in the elemental analysis compared to the untreated control. The exposed mannan side chains are the primary candidates showing maximum interaction with Cd(II) (Fig. 5). The enzyme degrades these $\beta$-mannan side chains resulting in significant decrease in Cd(II) interaction. The cells when treated with proteinase K didn’t show significant decrease in Cd(II) adsorption from the untreated control depicting less engagement of the functional groups of the peptide bonds of the more internally located proteins. Dual treatment with both enzymes dramatically lowered the Cd(II) adsorption even that of the $\beta$-mannanase treated ones depicting the involvement of both the mannan side chains and cell wall proteins in Cd(II) interaction. Snailase is basically an enzyme mixture comprising of multiple enzymes that can effectively digest the cell envelope components of microorganisms. Removing the entire cell wall with snailase showed minimal most amount of Cd(II) in the elemental composition depicting the principal role of the cell wall involved in Cd(II) interaction^71^.

#### Diffractometric evidence

##### XRD analysis

**Figure 6:**
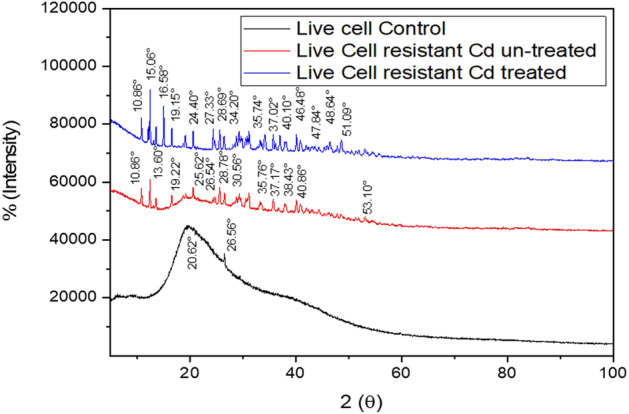
XRD analyses of various functional groups untreated (**a**) control yeast strain, (**b**) Cd(II) untreated resistant yeast strain and (**c**) treated resistant yeast strain (*Candida tropicalis* XTA 1874).

The XRD patterns of the live cells before (control) and Cd (II) treatment (Fig. 6) were recorded on a Rigaku smart-lab SE diffractometer using graphite monochromatic Cu (1.5406 Å)–K_α1_ (1.54439 Å) radiation. The scanning speed was maintained at 1.2 min^−1^ throughout the analysis. The amorphous nature of both the biosorbent (untreated and treated live cells) was determined from the intermediate peaks at 2θ range between 10 and 19°. The disappearance of peaks at the 2θ range of 16°, 20°, 25°, and 30° indicates the penetration of Cd (II) ions through the treated biosorbent surface. The crystalline vibration at the 2θ range from 46 to 51° indicates the presence of Cd (II) ions in treated biosorbent spectra. Besides, the amorphous nature of biosorbent was also seen in the short peaks from 24 to 28° and broad peaks from 35 to 40°.

#### Spectroscopic evidences

##### FT-IR analysis

**Figure 7:**
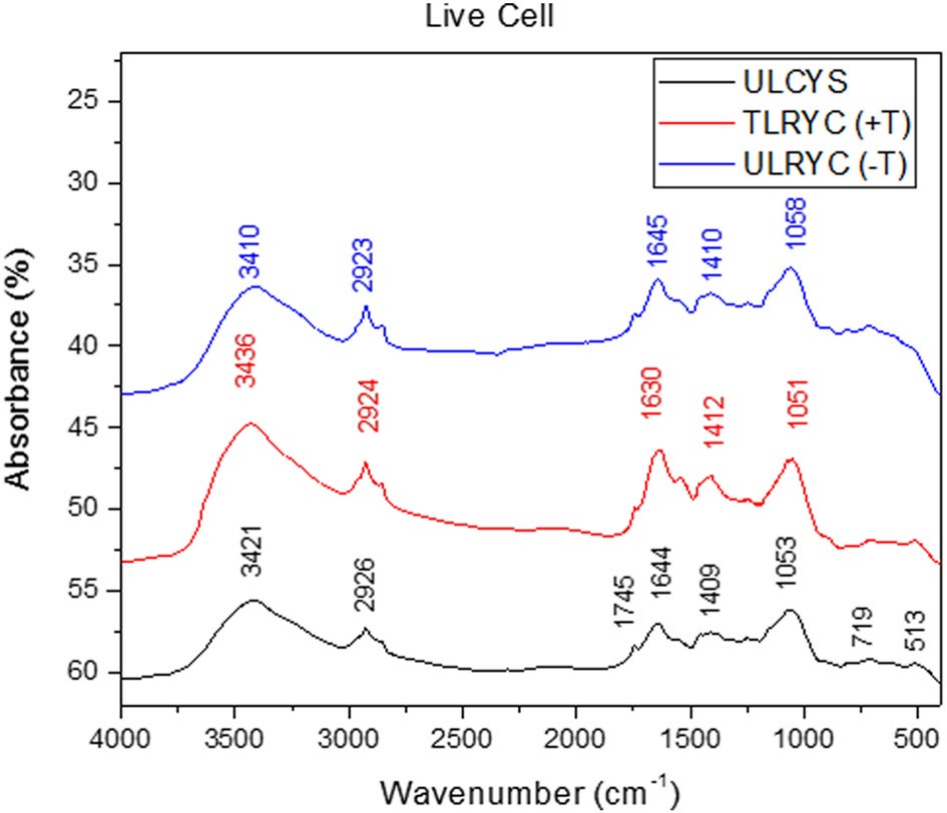
FT-IR spectrum of various functional groups (**a**) untreated control yeast strain (ULCYS), (**b**) Cd(II) treated resistant yeast strain [TLRYC (+ T)] and (**c**) untreated resistant yeast strain (*Candida tropicalis* XTA 1874) [ULRYC (− T)].

The FT-IR spectra (Fig. 7) show the functional group variation within the untreated live control yeast strain (ULCYS), untreated live resistant yeast cell [ULRYC (− T)] and treated live resistant yeast cell [TLRYC (+ T)] before and after Cd^2+^ adsorption. The bands between $3500\;{\text{and}}\; 3350 \;{\text{cm}}^{ - 1}$ were due to—OH and –NH stretching vibrations of the protein in the cells^83^. In comparison to ULCYS, both the TLRYC (+ T) and ULRYC (− T) spectra had sharper bands after the adsorption of Cd^2+^. The shoulder bands between $2950\;{\text{and}}\; 2920 {\text{cm}}^{ - 1}$ were due to the stretching vibrations of CH_2_^83^. The mild band at $1745 \;{\text{cm}}^{ - 1}$ was due to C=O stretching vibration of R−COOH in ULCYS^83^. The moderate bands between $1650\;{\text{and}}\; 1620 \;{\text{cm}}^{ - 1}$ were ascribable to C=O stretching vibration and free amide group of protein^83^. The reaction of the amide group with Cd^2+^ made the band at $1630 \;{\text{cm}}^{ - 1}$ [TLRYC (+ T)] more prominent. The bands at ${1412} \;{\text{cm}}^{ - 1}$ only appeared in the Cd^2+^ absorbed TLRYC (+ T) spectra. The sharp bands between $1060\;{\text{and}}\; 1050\; {\text{cm}}^{ - 1}$ were due to the C–O stretching vibration of carboxylic acid and C–N stretching of amide group. Both the TLRYC (+ T) and ULRYC (− T) spectra had sharper bands after the absorption of Cd^2+^. Overall, a discerning feature appeared in the sharper bands for Cd(II) adsorption with strain cell surface.

##### Raman spectral analysis

**Figure 8:**
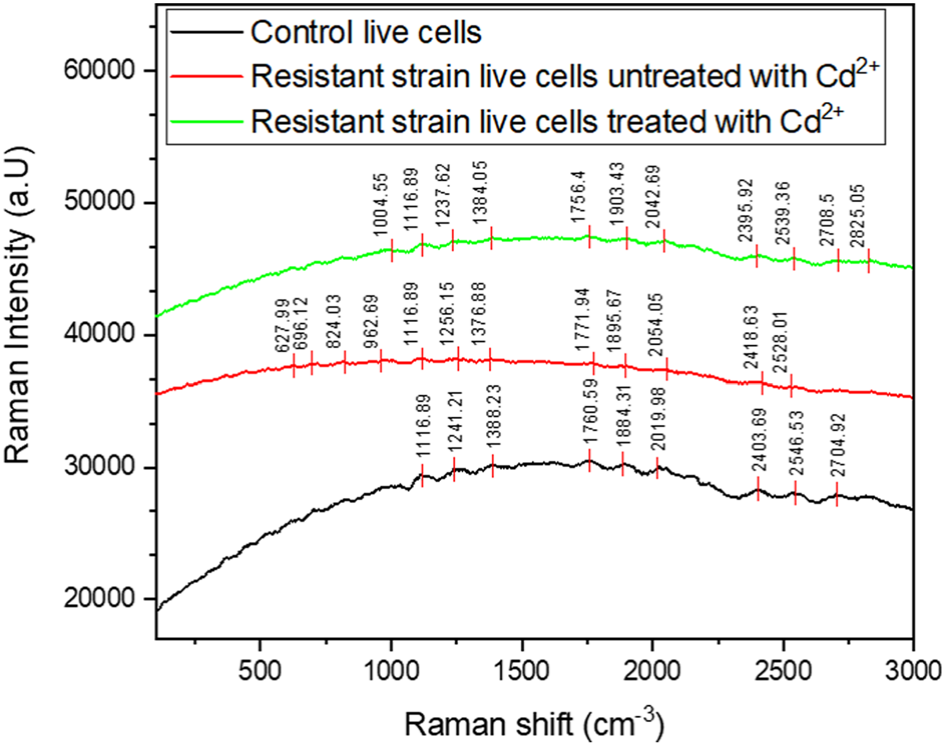
Raman spectrum of various functional groups (**a**) untreated control yeast strain, (**b**) Cd(II) untreated resistant yeast strain and (**c**) treated resistant yeast strain (*Candida tropicalis* XTA 1874).

Figure 8 shows the superficial interactions between the live cell and Cd^2+^ ions. The peaks located between $1115 - 1117 \;{\text{cm}}^{ - 1}$, $1230 - 1260\; {\text{cm}}^{ - 1}$, $1375 - 1390 \; {\text{cm}}^{ - 1}$, $1750 - 1772 \; {\text{cm}}^{ - 1}$, $2018 - 2055 \; {\text{cm}}^{ - 1}$ and $2395 - 2547 \; {\text{cm}}^{ - 1}$ indicated C–C, C–H, C–N, C=O, and S–N bond vibrations^84–87^. In comparison to the control and resistant strain, there was a minor decrease in the intensity of the peaks after Cd^2+^ adsorption. This indicated that the C–C, C–H, C–N, C=O, and S–N bond vibrations did have a very minor impact on the Cd^2+^ adsorption process. However, the increase in the peak intensity between $2704\;{\text{and}}\; 2826\;{\text{ cm}}^{ - 1}$ indicated the involvement of C–H vibration in the Cd^2+^ process^86^. The intensity of the peaks was more prominent in the live cell-resistant strain treated with Cd^2+^. The peak at $2825.05 \;{\text{cm}}^{ - 1}$ indicates the involvement of methyl or formyl groups in the Cd^2+^ adsorption process^86^. The peaks between $627\;{\text{and}}\; 962\;{\text{ cm}}^{ - 1}$ indicate τ and ω ring deformation of C–H vibration vanished after the adsorption of Cd^2+^ ions in the live cell resistant strain^85^. The peak at $1004.55 \;{\text{cm}}^{ - 1}$ indicates the involvement of amino-acid side chains of resistant strain in adsorbing the Cd^2+^ ions^87^.

##### XPS analysis

**Figure 9:**
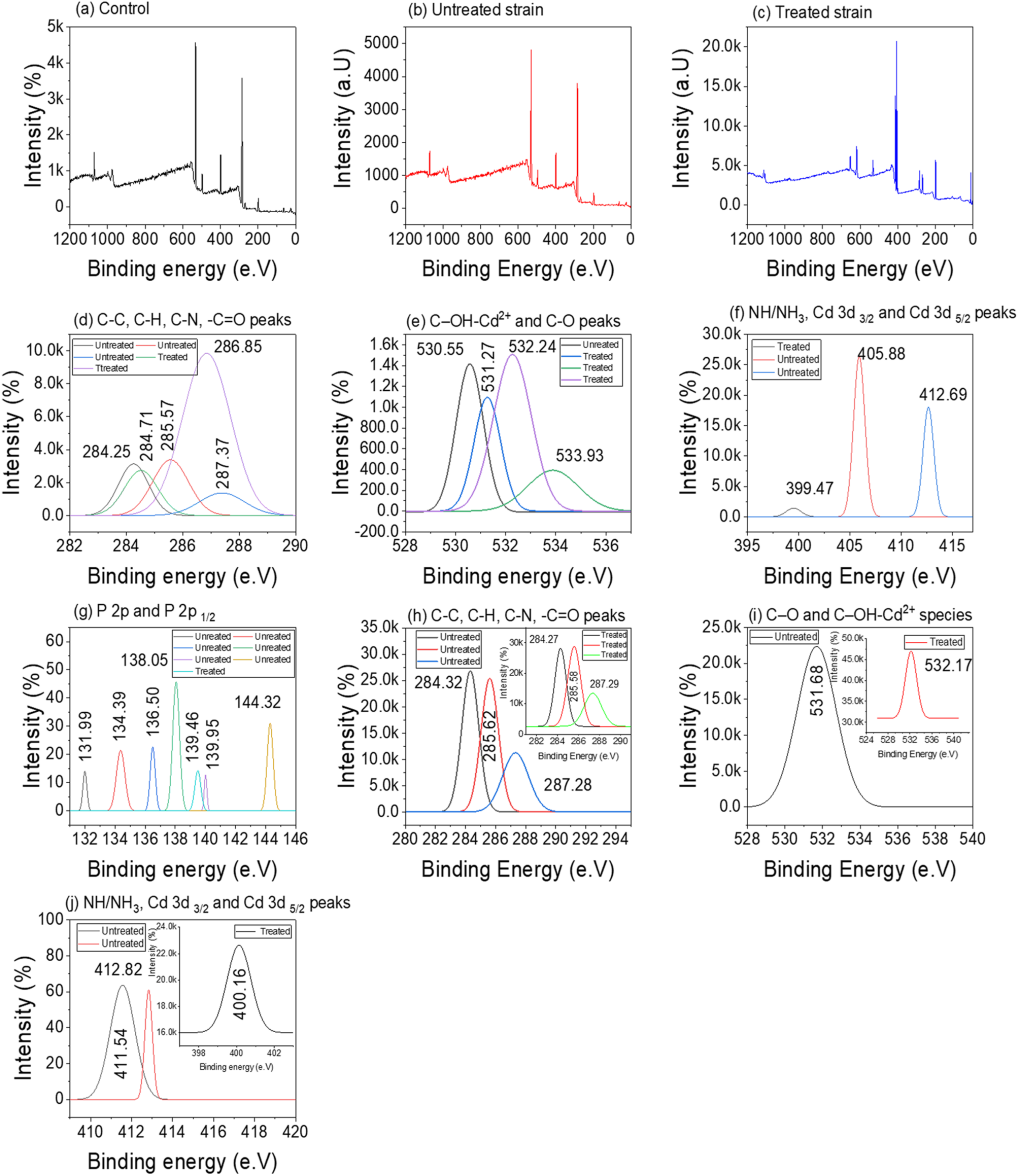
XPS analyses of various functional groups untreated control yeast strain, Cd(II) untreated resistant yeast strain and treated resistant yeast strain (*Candida tropicalis* XTA 1874).

Fig. 9(a–j) shows the elemental composition change of the resistant strain *Candida tropicalis* XTA1874 before and after Cd^2+^ with binding energy and atomic content. The change in the survey and high-resolution Cd 3*d* spectrum (Fig. 9b) was evident after the adsorption of Cd^2+^ on the surface of the resistant strain. The high-resolution C 1*s* spectrum of both untreated and treated [Fig. 9(d,h)] peaks at 284.25 eV, 284.27 eV, 284.32 eV, 284.53 eV, and 284.71 eV binding energy correspond to C–C and C–H species^87,88^. After adsorption, the atomic content of C–C species [Fig. 9(d,h)] decreased from 46.67 to 14.64% and C–H from 53.28 to 22.85%. Similarly, the atomic content of the C–N species decreased from 37.40% (285.57 eV) and 58.58% (285.59 eV) to 6.36% (285.62 eV). However, the atomic content of –C=O species [287.37 eV (11.83%), 286.85 eV(18.57%), and 14.32% (287.28 eV)] moreover remained the same after Cd^2+^ adsorption. Figure 9(e,i) show a high-resolution O 1*s* spectrum. After adsorption, the atomic content of C–O decreased from 44.98% (531.27 eV) to 21.22% at 531.68 eV. The disappearance of the 530.55 eV (10.82%) peak after adsorption might be due to the formation of the C–OH–Cd^2+^ species. Whereas, the peaks at 532.24 eV (71.54%), 532.17 eV (52.14%) and 533.93 eV (7.24%) correspond to carbonyl oxygen atoms in esters and anhydrides^87,88^. The deconvolution of the O 1*s* spectrum at 532 eV and 533 eV peaks arose due to the oxygen atoms in hydroxyl groups and the non-carbonyl oxygen atoms in the ester groups^87,88^. Before bio-sorption, the presence of NH or NH_2_ species was determined by the peak at 399.47 eV (6.52%) binding energy [Fig. 9f]. After adsorption, the deconvolution of the N 1*s* spectrum shows a peak at 400.16 eV [Fig. 9j] with atomic content of 2.31% corresponding to the donation of lone pair of N electrons to Cd^2+^^89^. A similar observation was also noticed in Fig. 9(f,j) before and after the adsorption of Cd^2+^. The high-resolution spectrum at Cd 3*d*_3/2_ and _5/2_ arose due to the formation of the ester-Cd^2+^ complex ions^87^. The atomic content of the peak of Cd 3*d*_5/2_ at 405.88 eV was 3.01% after adsorption^87^. However, the atomic content of the peaks of Cd 3*d*_3/2_ at 412.69 and 412.82 reduce to 6.47 and 5.99% after adsorption. The Cd 3*d*_3/2_ peak at 411.54 eV had atomic content of 21.58% before bio-sorption. The weaker P 2*p*_1/2_ peaks at 131.99 eV, 134.39 eV, 138.05 eV, 139.46 eV, 139.95 eV, and 144.32 eV correspond to P–O–C or P–O species before bio-sorption^90^. The occurrence of the P 2*p* peaks at 136.50 eV and 139.46 eV corresponds to the formation of phosphorous-Cd^2+^ ions after adsorption^90^. The biogeochemistry of heavy metals can easily be understood from the binding mechanisms of Cd^2+^ ion by the resistant strain *Candida tropicalis* XTA1874.

### Adsorption equilibrium, thermodynamic and kinetic analyses

**Figure 10:**
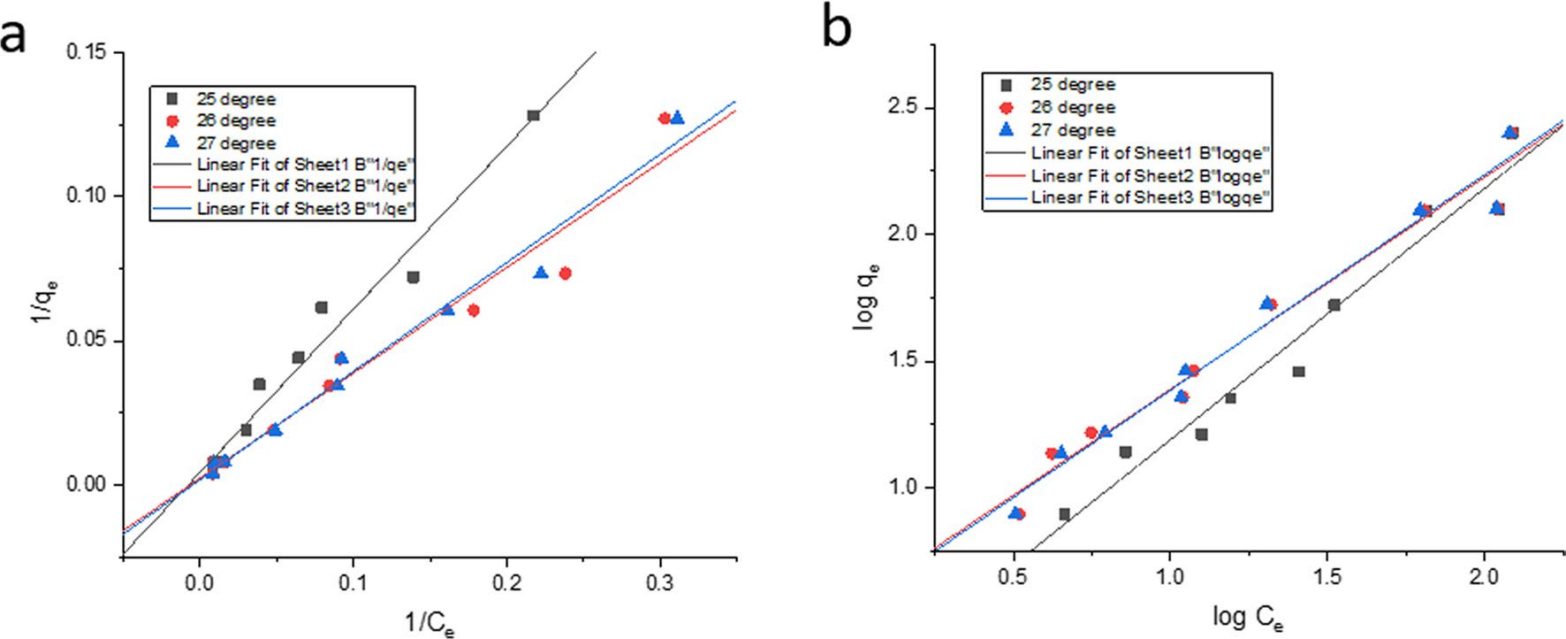
Adsorption equilibrium isotherm linear plots for Langmuir (**a**) and Freundlich (**b**) Model for Cd(II) Biosorption by the strain *Candida tropicalis* XTA 1874.

Adsorption isotherms define the equilibrium relations between the adsorbate concentration on the adsorbent phase and its concentration in the bulk solution. From the isotherms we can evaluate the maximum adsorption capacity. These data give us information on the adsorbent capacity or its amount required to remove a unit mass of pollutant under the system conditions. Langmuir and Freundlich isotherms are the most commonly used adsorption isotherm models describing solid–liquid adsorption systems. The Langmuir and Freundlich isotherm constants have been calculated after linear fitting are shown in (Table 4). It is evident there that both Langmuir and Freundlich models describe well the experimental data. Analysis of the mathematical description of Cd(II) biosorption equilibrium in the resistant strain depicts that it is best described by using the Langmuir model (Table 4, Fig. 10). It is evident from the linear plots, ${R}^{2}$ value of the Langmuir model at $27\;^{ \circ } {\text{C}}$ is greater and best fits $\left( {R^{2} = 0.978} \right)$ the adsorption data obtained by live cells of *Candida tropicalis* XTA 1874. The value of $R_{l}$ $\left( {0.055 \pm 0.000 - 0.0018 \pm 0.000} \right)$ depicts that the favorability of the Cd(II) adsorption process by the strain. Since the values of the $R_{l}$ is between $0$ and $1$ (0 < $R_{l}$<1) which gives a reliable indication of the adsorption process by the strain follows the Langmuir model. At $27\;^{ \circ } {\text{C}}$, $26\;^{ \circ } {\text{C}}$ and $25\;^{ \circ } {\text{C}}$ significant differences in the values of the correlation coefficients $\left( {R^{2} } \right)$ for both models (Langmuir and Freundlich) were found, that is $(0.978 > 0.977 > 0.968)$ and $(0.973 > 0.972 > 0.962)$ respectively. An overall mean equilibrium biosorption capacities have been obtained were $66.327 \pm 0.001\%$, $74.221 \pm 0.000\%$ and $74.579 \pm 0.004\%$ at temperatures $25\;^{ \circ } {\text{C}}$, $26\;^{ \circ } {\text{C}}$ and $27\;^{ \circ } {\text{C}}$ respectively.

**Table 4:** Values of the adsorption equilibrium isotherm model and thermodynamic parameters for Cd(II) in *Candida tropicalis* XTA 1874 [Mean ± SEM, Sample Size (n) = 6].

Langmuir	$q_{{{\text{max}}}} ({\text{mgg}}^{ - 1} )$	$K_{l}$ $\left( {{\text{L}}\;{\text{mg}}^{ - 1} } \right)$	$R^{2}$	$R_{l}$	Mean Removal (%)	$\Delta G^{0}$ $\left( {{\text{kJ}}\;{\text{mol}}^{ - 1} } \right)$	$\Delta H^{0}$ $\left( {{\text{kJ mol}}^{ - 1} } \right)$	$\Delta S^{0}$ $\left( {{\text{KJ K}}^{ - 1} {\text{mol}}^{ - 1} )} \right)$
25 °C	233.579 ± 2.711	1.016 ± 0.233	0.968	0.002 ± 0.001–0.062 ± 0.000	66.331 ± 0.003	− 0.039 ± 0.000	47.574 ± 0.002	0.159 ± 0.000
26 °C	420.111 ± 3.15	1.113 ± 0.243	0.977	0.0019 ± 0.000–0.055 ± 0.000	74.220 ± 0.000	− 0.265 ± 0.008		
27 °C	544.113 ± 6.071	1.155 ± 0.273	0.978	0.0018 ± 0.000–0.055 ± 0.000	74.584 ± 0.001	− 0.358 ± 0.000		
Freundlich	$K_{f} ({\text{Lmg}}^{ - 1} )$	$n$	${R}^{2}$					
25 °C	1.576 ± 0.000	1.151 ± 0.001	0.962					
26 °C	3.557 ± 0.002	1.145 ± 0.007	0.972					
27 °C	3.226 ± 0.044	1.145 ± 0.006	0.973

The maximum adsorption capacity (${q}_{max}$) using the Langmuir model at $25\;^{ \circ } {\text{C}}$, $26\;^{ \circ } {\text{C}}$ and $27\;^{ \circ } {\text{C}}$ were $233.579 \pm 2.711$, $420.111 \pm 3.15$ and $544.113 \pm 6.071$, respectively. After increasing the temperature to $28\;^{ \circ } {\text{C}}$, no further increase in the value of $q_{max}$ was observed. From the above observation, optimum temperature for Cd(II) biosorption by the resistant strain has been determined to be $27\;^{ \circ } {\text{C}}$. Adsorption capacity increases with increasing temperature is evident too along with increasing $K_{l}$ value, which has however slightly decreased beyond the optimum temperature $\left( {27\;^{ \circ } {\text{C}}} \right)$. Increasing temperature facilitating Cd(II) biosorption capacity was also obtained by using *Vigna radiata* L. biomass^91^. The biosorption best fitted the Langmuir model among all other isotherm models used in the study too. Increasing adsorption temperature aggravates Cd(II) adsorption was evidenced in studying biosorption by waste mangosteen shell^92^.

In case for the Freundlich model, the $n$ being a dimensionless factor depicting the Cd(II) sorption intensity or the inhomogeneous nature of the adsorbent surface^74,93^. Logarithimic expression of experimental data was performed using the linearized equation of the Freundlich isotherm model (Fig. 10b). The numerical values of all the parameters evaluated at different temperatures along with the correlation coefficient were depicted in Table 4. The parameter $n$, which measures the Cd(II) adsorption intensity by the strain, demonstrated that the values ranging from $1.145\pm 0.006-1.151\pm 0.001$. The obtained values were in the range of (1 < $n$<10), which confirms the efficiency of the biosorption process.

**Figure 11:**
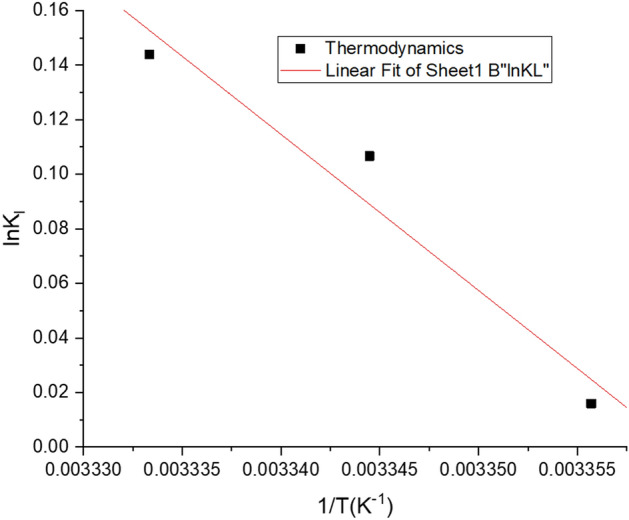
Graphical representation of Cd(II) Biosorption thermodynamics from linear plot of lnK_l_ versus 1/T.

Thermodynamic parameters such as change in enthalpy ($\Delta {H}^{0}$), change in entropy ($\Delta {S}^{0}$) and the Gibbs free energy change ($\Delta {G}^{0}$) for the biosorption process have been calculated from the changes in the values of the Langmuir constant (${K}_{l}$) with temperature ($T$) using the equations:

15 $$\Delta {G}^{0} = -RT ln {K}_{l}$$

16 $$\Delta {G}^{0} = \Delta {H}^{0}- T\Delta {S}^{0}$$

17 $$ln{K}_{l}= -\frac{\Delta {H}^{0}}{R}\left(\frac{1}{T}\right)+\frac{\Delta {S}^{0}}{R}$$

where $R$ is the universal gas constant $\left( {8.314 \times 10^{ - 3} \; {\text{kJ mol}}^{ - 1} \; {\text{K}}^{ - 1} } \right)$ and the Langmuir constant is $K_{l}$. The enthalpy change owing to adsorption of Cd(II) over the temperature range has been determined form the linear plots of $K_{l}$ versus $\frac{1}{T}$ using least square analysis. As is evident from Table 5, Fig. 11, the changes in the values of $\Delta G^{0}$ are small and gradually decrease with increasing temperatures. The negative values of the Gibbs free energy change amply demonstrate the spontaneity of the process. The positive values of $\Delta H^{0}$ indicate the endothermic nature of the process. It implied that some energy input is required for the adsorption process from outside. The positive value of the entropy change ($\Delta S^{0}$) indicated the increase in the randomness at the solute/solvent interface during biosorption of Cd(II) ions on the cell surface of the developed resistant strain^94^.

**Table 5:** Kinetic model parameters of Cd(II) adsorption in *Candida tropicalis* XTA 1874 [Mean ± SEM, Sample Size (n) = 6].

Pseudo first order					Pseudo second order		
Metal ion (ppm)	$q_{{{\text{e}}_{{\text{exp }}} }} \left( {{\text{mgg}}^{ - 1} } \right)$	$k_{1}$	$R^{2}$	$q_{{{\text{e}}_{{\text{cal }}} }} \left( {{\text{mgg}}^{ - 1} } \right)$	$k_{2}$	$R^{2}$	$q_{{e_{cal } }} \left( {{\text{mgg}}^{ - 1} } \right)$
100	52.828 ± 0.000	− 0.0001 ± 5.44E^−07^	0.982	7.733 ± 0.001	0.0005 ± 1.32E^−05^	0.934	51.048 ± 0.00
250	124.672 ± 0.001	− 4.503 ± 6.33E^−07^	0.799	5.471 ± 5.64E^−05^	0.021 ± 0.001	1	122.406 ± 0.005
300	126.439 ± 0.001	− 5.3E^−05^ ± 8.64E^−07^	0.816	4.078 ± 5. 41E^−05^	0.024 ± 0.000	1	125.173 ± 0.016
500	252.395 ± 0.000	0.028 ± 0.000	0.961	2.438 ± 0.000	1.402 ± 0. 047	1	256.242 ± 1.173

Biosorption kinetics indicates the rate at which adsorption of Cd(II) occurs over the surface of the developed resistant strain. The rate constants were calculated by using pseudo first order and pseudo second order kinetic models^87,92,94^. The biosorption kinetic study was performed at $pH 6.3$ and temperature $27 \;^{ \circ } {\text{C}}$. Kinetic studies of Cd(II) biosorption by the strain was carried out using the concentrations from $100\;{\text{to}}\;500 \;{\text{ppm}}$ of Cd(II). Adsorption kinetics constitutes two phases: a stage of rapid removal of the toxicant, followed by a slower phase before reaching the equilibrium. Fast and maximum Cd(II) removal was observed in the initial $80 \;{\text{min}}$ of contact time. The biosorption efficacy increased with increasing contact time and ultimately reached equilibrium after $150\;{\text{ min}}$ for all the concentrations [$100 - 500 \;{\text{ppm}}$ Cd(II)] used in this study. The reason behind this is due to the availability of the binding site which gradually declined with the progress of biosorption.

**Figure 12:**
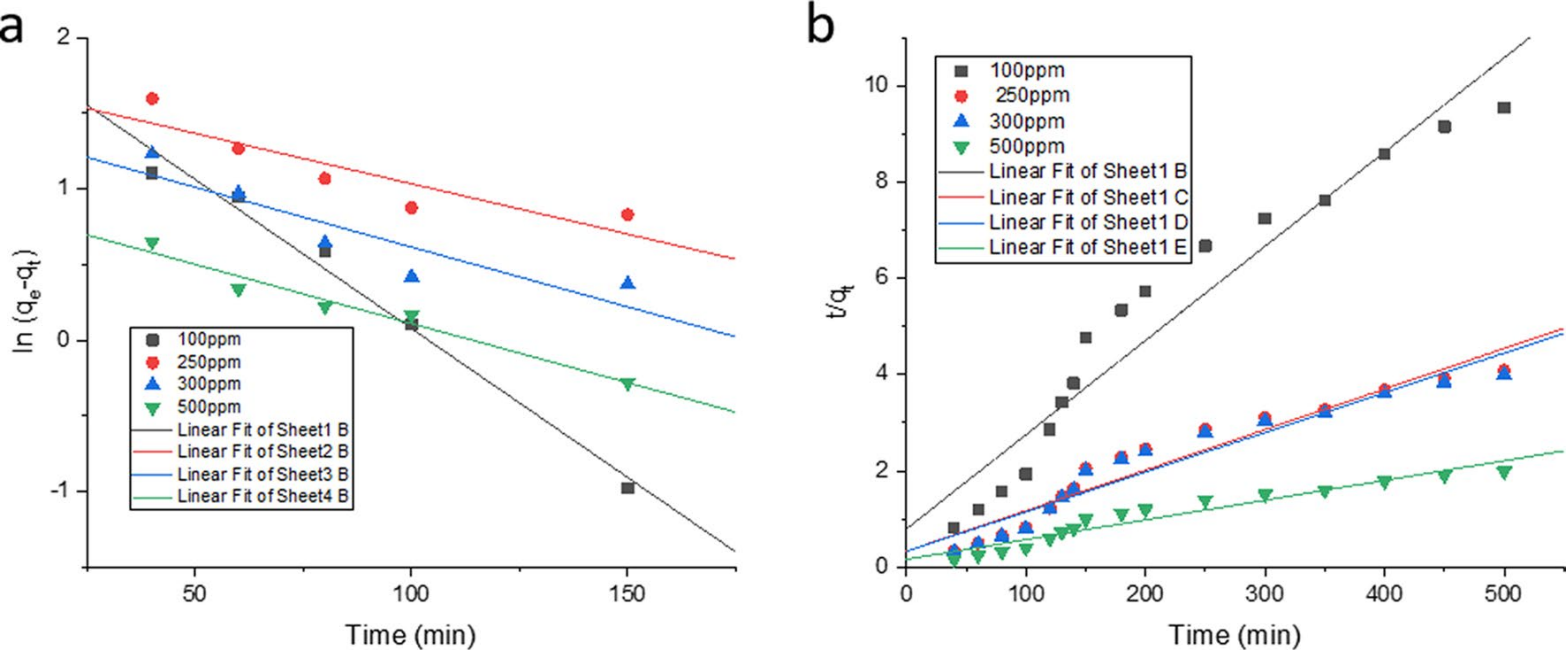
Linear plots for pseudo first (**a**) and second order (**b**) kinetic models For Cd(II) Biosorption by the strain *Candida tropicalis* XTA 1874 at 27 °C

The $q_{{e_{cal} }}$ values calculated from the pseudo first order kinetic model differed considerably from the experimental values as evidenced from Table 5 and Fig. 12. In the pseudo second order model the calculated $q_{{e_{cal} }}$ values are very close to the experimental $q_{{e_{exp} }}$. This indicates that the Cd(II) adsorption data best fitted the pseudo second order ($R^{2} = 1$) for *Candida tropicalis* XTA 1874. Since, from this finding it can be assumed that the adsorption rate is a function of squared number of unoccupied sites. Because of the best fit of the pseudo second order kinetic model in defining the adsorption process we can assume that adsorption mechanism mutually depended on both the Cd(II) solution and the type of the biosorbent, and the rate limiting step occurs due to the chemisorption^78,95^. But we can’t affirm to the decision about the mechanistic insights of the process until we go for activation energy ($E_{a}$) calculation^96,97^. The value of the rate constant ($k_{2}$) was obtained from the plot of $\frac{t}{{q_{t} }}$ versus $t$ (Fig. 12b).

**Figure 13:**
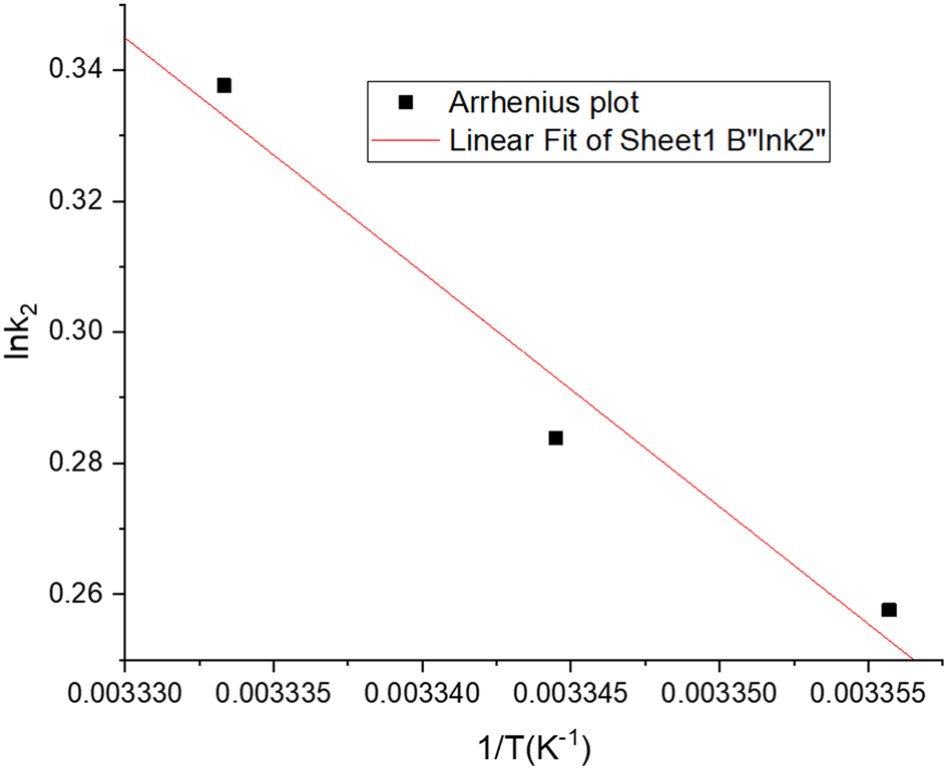
Determination of activation energy by Arrhenius plot.

Kinetic parameters at the same temperature range $\left( {25 - 27\;^{ \circ } {\text{C}}} \right)$ have been plotted again by means of using pseudo first and second order kinetic models (Table 6). Liner plot of $ln {k}_{2}$ versus $\frac{1}{T}$ (Fig. 13) has been used to determine the activation energy using the Arrhenius Eq.:

18 $$\ln \;K_{2} = \ln K_{0} - \frac{{E_{a} }}{RT}$$

where $K_{2}$ is the pseudo-second order rate constant (${\text{g}} \;{\text{mg}}^{ - 1} \;{\text{min}}^{ - 1}$), $k_{0}$ is the independent temperature factor (${\text{g}}\; {\text{mg}}^{ - 1} \;{\text{min}}^{ - 1}$), $R$ is the gas constant (${\text{J}}\;{\text{mol}}^{ - 1} {\text{K}}^{ - 1}$), and $T$ is the solution temperature $\left( K \right)$. A straight line obtained from the linear plot of $ln k_{2}$ versus $\frac{1}{T}$ and activation energy ($E_{a}$) has been calculated from slope of the plot. The activation energy for Cd(II) by the developed resistant strain has been given in Table 6. The magnitude of activation energy explains the nature of intensity and the type of the adsorption process which can be principally classified as physical or chemical adsorption (chemisorption). In chemical adsorption, the activation energy is typically higher than $4.2 \;{\text{kJ mol}}^{ - 1}$, whereas in physical adsorption, the opposite is true^98^. The magnitude of the activation energy ($29.756 \pm 0.002\;{\text{kJ}}\;{\text{mol}}^{ - 1}$) obtained in case of Cd(II) biosorption by the strain *Candida tropicalis* XTA1874 supports the chemical nature of the adsorption process.

**Table 6:** Kinetic model parameters of Cd(II) adsorption in *Candida tropicalis* XTA 1874 at 25 °C, 26 °C, 27 °C using 500 ppm Cd(II) [Mean ± SEM, Sample Size (n) = 6].

			Pseudo first order			Pseudo second order						
Temperature (°C)	Metal ion (ppm)	$q_{{e_{{{\text{exp}}}} }}$ ${\text{mgg}}^{ - 1}$	$k_{1}$	$R^{2}$	$q_{{e_{cal} }} mgg^{ - 1}$	$k_{2}$	$R^{2}$	$q_{{e_{cal} }} mgg^{ - 1}$	$E_{a} \;{\text{kJmol}}^{ - 1}$	$\Delta G^{\# } \;{\text{kJmol}}^{ - 1}$	$\Delta H^{\# } \;{\text{kJmol}}^{ - 1}$	$\Delta S^{\# } \;{\text{kJk}}^{ - 1} \;{\text{mol}}^{ - 11}$
25	500	251.879 ± 0.0002	− 2.3 E^−05^ ± 1.99E^−06^	0.878	6.449 ± 0.012	1.294 ± 0.293	1	249.434 ± 0.293	29.756 ± 0.003	44.131 ± 0.005	32.242 ± 0.003	− 0.0397 ± 0.0004
26			− 5.8 E^−05^ ± 2.64 E^−06^	0.726	4.635 ± 0.124	1.328 ± 0.311	1	251.453 ± 0.311		44.164 ± 0.002		
27			0.028 ± 0.0001	0.961	2.438 ± 0.0004	1.402 ± 0.407	1	256.242 ± 0.406		44.209 ± 0.004

**Figure 14:**
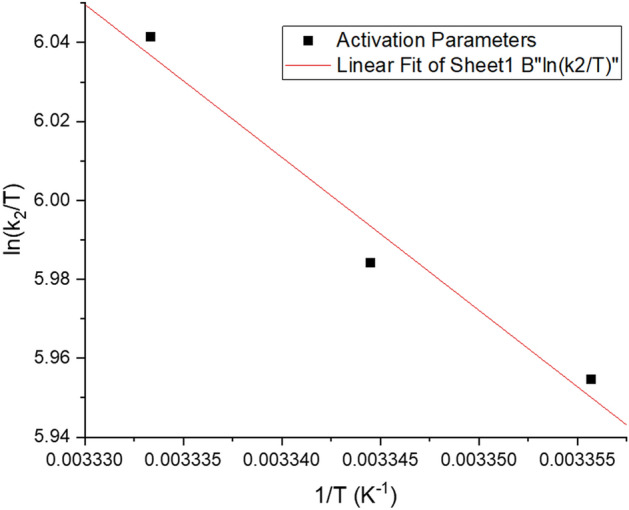
Determination of activation parameters for adsorption of Cd(II) on the resistant strain *Candida tropicalis* XTA 1874.

With the Eyring equation, activation parameters like enthalpy, entropy, and free energy can be computed^99^.

19 $$ln\left(\frac{{k}_{2}}{T}\right)= \left[ln\left(\frac{{k}_{B}}{h}\right)+\frac{\Delta {S}^{\#}}{R}\right]-\frac{\Delta {H}^{\#}}{RT}$$

where ${k}_{B}$ is the Boltzmann constant $\left( {1.3807 \times 10^{ - 23} { }\;{\text{J K}}^{ - 1} } \right)$ is Planck’s constant $\left( {6.6261 \times 10^{ - 34} \; {\text{J s}}} \right)$, and ${k}_{2}$ is the pseudo second-order rate constant. Figure 14 displays the plot of $ln\left(\frac{{k}_{2}}{T}\right)$ against $\frac{1}{T}$. One way to compute Gibbs energy of activation is to

20 $$\Delta {G}^{\#} = \Delta {H}^{\#} - T\Delta {S}^{\#}$$

From Table 6 it is evident that the shift in activation of the Gibbs energy ($\Delta {G}^{\#}$) for the adsorption of Cd(II) ions on *Candida tropicalis* XTA 1874 was determined to be $+ 44.205 \;{\text{kJ }}\;{\text{mol}}^{ - 1}$ at $300 \;{\text{K}} \left( {27\;^{ \circ } {\text{C}}} \right)$. It suggests that energy is needed for adsorption processes^98,100^. When compared to the outgoing ion, the divalent metal ions’ mobility within the adsorbent is more constrained, as indicated by the negative values of $\Delta {S}^{\#}$^98^. The development of an activated complex as a result of Cd(II) sorption is demonstrated by the negative $\Delta {S}^{\#}$ values, suggesting that Cd(II) adsorption onto the adsorbent is a related mechanism. The fact that the value for ($\Delta {G}^{\#}$) found to be positive demonstrating the presence of an energy barrier for the Cd(II)^98^. It also suggests that energy must be added in order for reactant molecules to have sufficient kinetic energy to cross the energy barrier and initiate a chemical reaction^98^.

### Desorption studies

#### Analysis of the desorption efficiency and reusability of the biomass

**Figure 15:**
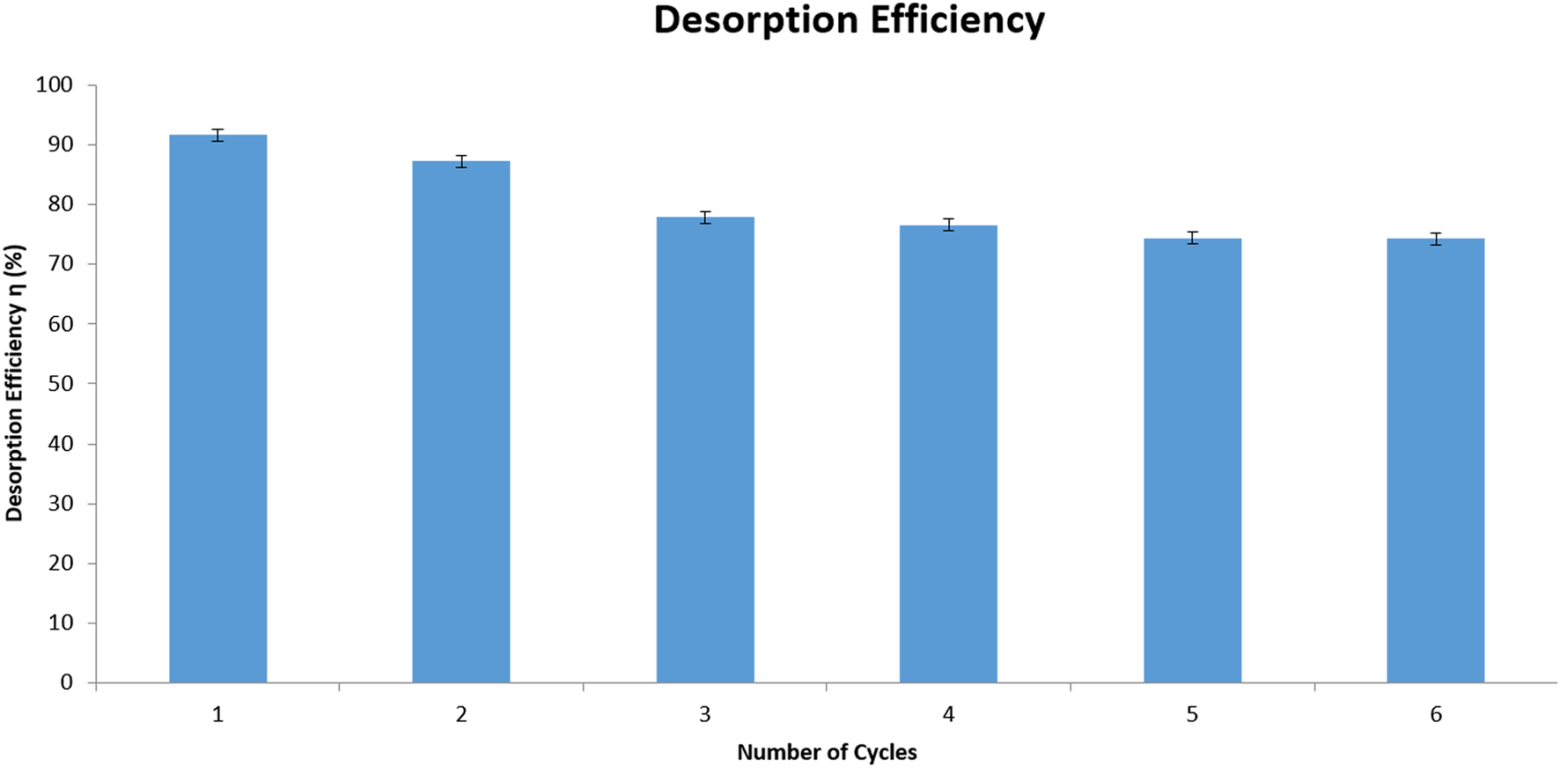
Graphical representation of the Cd(II) desorption efficiences with the number of cycles using the developed resistant strain *Candida tropicalis* XTA 1874.

Desorption efficiency ($\eta$%) and reusability of the biomass plays a pivotal role in making the water treatment as a cost effective process^82,101^. It is evident from (Table 7, Fig. 15) that biomass from the developed resistant strain showed quite efficient desorption capacity $(87.527\pm 0.023\%)$ at the first round of the desorption experiment. The biosorbent was reused with slight decrease in the adsorptive removal and desorption efficiency ($\eta$%). Desorption analysis was carried out for five cycles after which no significant change in desorption efficiency was observed. In each reusage cycle of the biomass the surface (not removed) and intracellularly accumulated amount $\left( {{\text{mgg}}^{ - 1} } \right)$ has been shown which was determined by EDTA chelation and acid digestion respectively^51^ (Table 7). In the due course of kinetic analysis equilibrium was reached at $150\; {\text{min}}$ of contact time with the eluent and a little retention of Cd(II) ($q_{t} = 0.012 \;{\text{mgg}}^{ - 1}$). The terms $q_{i}$ and $q_{t}$ signifies the initial amount $\left( {{\text{mgg}}^{ - 1} } \right)$ of surface accumulated retained Cd(II) in the biomass at time $(t)$ after contact with the eluent solution respectively.

**Table 7:** Estimation of desorption capacity (η %) and the regeneration capacity of the biomass [Mean ± SEM, Sample Size (n) = 6].

Number of Cycles	$C_{r}$	$C_{i}$	$C_{e}$	$V$		Adsorbed (ppm)	Absorbed (ppm)	Surface accumulation (mg/g)	Intracellular accumulation (mg/g)	$V_{r}$	Removal (%)	$\eta$%
1	318.279 ± 0.000	500	121.541 ± 0.000	0.1		378.411 ± 0.007	0.04 ± 0.0005	252.274 ± 0.005	0.027 ± 0.000	0.1042	96.095 ± 0.073	87.527 ± 0.023
2	315.48 ± 0.000	500	124.28 ± 0.000	0.1		375.68 ± 0.000	0.04 ± 8.17 E^-12^	250.453 ± 0.000	0.026 ± 5.44E^−12^ 0.02 ± 4.82E^-17^	0.1034	85.577 ± 0.012	86.885 ± 0.098
3	312.219 ± 0.000	500	128.281 ± 0.000	0.1		371.691 ± 0.000	0.03 ± 7.2 E^-17^	247.794 ± 0.000	0.013 ± 0.000	0.103	80.008 ± 0.044	86.52 ± 0.0001
4	298.279 ± 0.000	500	132.361 ± 0.001	0.1		367.621 ± 0.001	0.02 ± 0.000	245.081 ± 0.000	0.007 ± 0.000	0.102	79.727 ± 0.16	82.768 ± 0.007
5	282.279 ± 0.000	500	132.48 ± 0.001	0.1		367.51 ± 0.000	0.01 ± 0.000	245.01 ± 0.000	0.005 ± 3.6E^-07^	0.102	77.514 ± 0.002	78.345 ± 0.0001
6	281.949 ± 0.000	500	132.481 ± 0.000	0.1		367.511 ± 0.000	0.009 ± 5.39 E^−07^	245.007 ± 0.000		0.102	77.514 ± 0.002	78.265 ± 0.011

**Figure 16:**
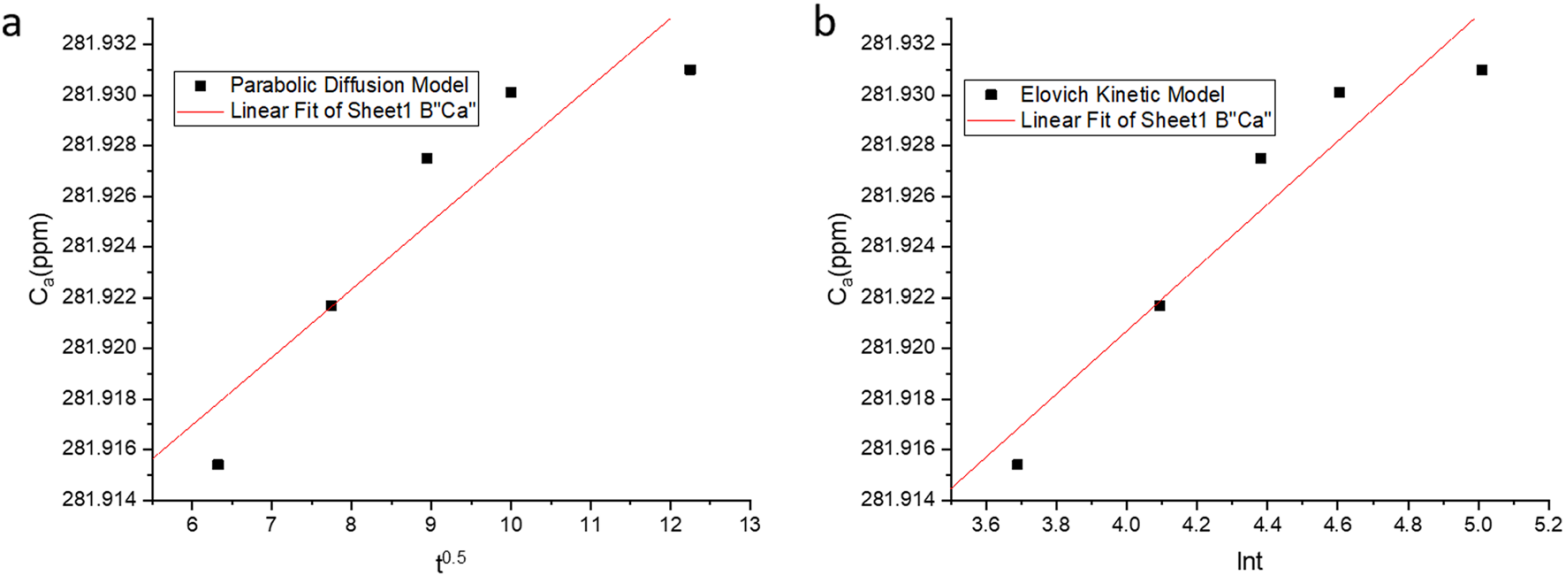
Evaluation of Cd(II) desorption kinetics by parabolic diffusion (**a**) and Elovich model (**b**) by Cd(II) resistant strain *Candida tropicalis* XTA 1874.

**Figure 17:**
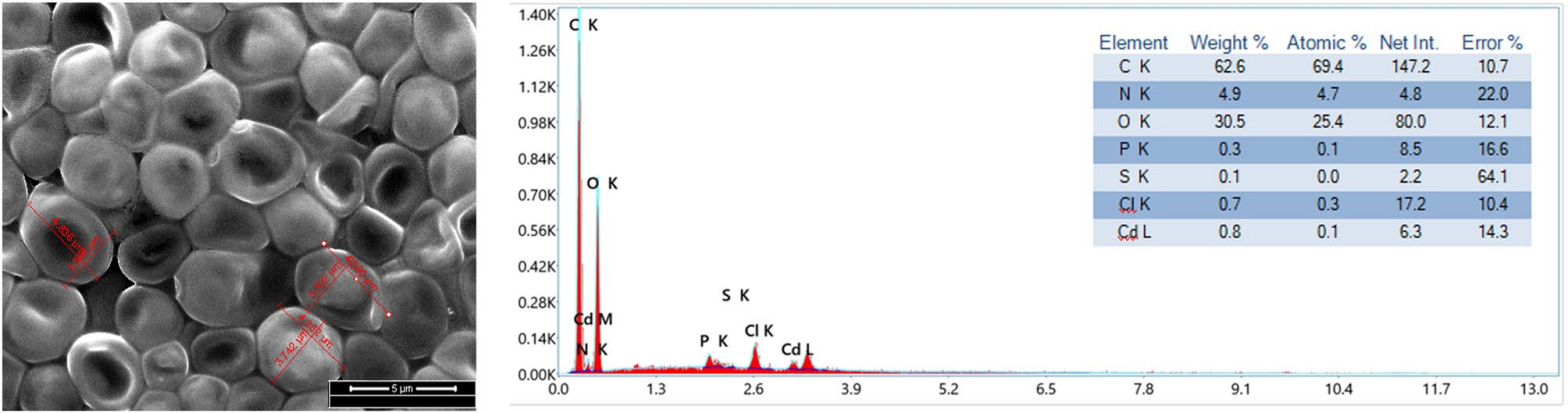
FE-SEM and EDAX analyses of developed Cd(II) resistant strain *Candida tropicalis* XTA 1874 biomass after desorption.

Desorption kinetic analyses were carried out using liner plotting of parabolic diffusion model and Elovich-type model (Table 8, Fig. 16)^101^. FE SEM image with EDAX analysis of the cells $\left( {3.535 \pm 0.127\;{\mu m} \times 4.592 \pm 0.139 \;{\mu m}} \right)$ after desorption have been shown in (Fig. 17). EDAX analysis showed a little retention of Cd(II) $\left( {0.8\;{\text{wt}}\% } \right)$ even after desorption of Cd(II) from the biomass.

**Table 8:** Estimated desorption kinetics parameters [Mean ± SEM, sample size (n) = 6].

Model	Equation	Parameters	${C}_{{a}_{0exp}}$	${C}_{{a}_{0cal}}$	$\alpha$	$\beta$	${R}^{2}$	$SE$
Parabolic Diffusion Model	$$\begin{aligned} \frac{1}{{C_{a} }} & = \frac{1}{{C_{a0} }} \\ & \quad - k_{{a_{2} }} t \\ \end{aligned}$$	C_a_, Cd(II) released at Time t C_a0_, Cd(II) concentration in solution when all ions released	281.95	281.895 ± 0.004	–	–	0.847	0.068
Elovich type model	$$\begin{aligned} C_{a} & = \frac{1}{\beta }ln\alpha \beta \\ & \quad + \frac{1}{\beta }lnt \\ \end{aligned}$$	α, initial Cd(II) ion desorption rate (mgL^−1^ min^−1^) β, desorption rate constant (mgg^−1^)	281.95	281.925 ± 0.018	2.599E+^122^	80.192	0.913	0.059

To analyze the best fitting of the models, the coefficient of determination ${(R}^{2})$ and standard error of estimate $(SE)$ were calculated by the following formula

21 $$SE = \sqrt {\frac{{\sum \left( {C_{i} - C_{i}{\prime} } \right)^{2} }}{N - 2}}$$

[${C}_{i}$ and ${C}_{i}{\prime}$ define measured and calculated Cd(II) in solution, $N$ is the sample size $(6)$].

In the Elovich Model it was assumed that $\alpha \beta t>>1$^102,103^.

Based on the values of ${R}^{2}$ and $SE$ (Table 8), it can be demonstrated that desorption kinetics followed the Elovich Kinetic Model where the calculated and experimental values of ${C}_{{a}_{0}}$ were very close. The derived parameter data complied with the Elovich model assumption $\alpha \beta t>>1$^101^. Cd(II) release from soil has been tested by various organic acids where it has been found that parabolic diffusion best fitted Cd(II) desorption kinetics^104^.

#### FT-IR analysis

**Figure 18:**
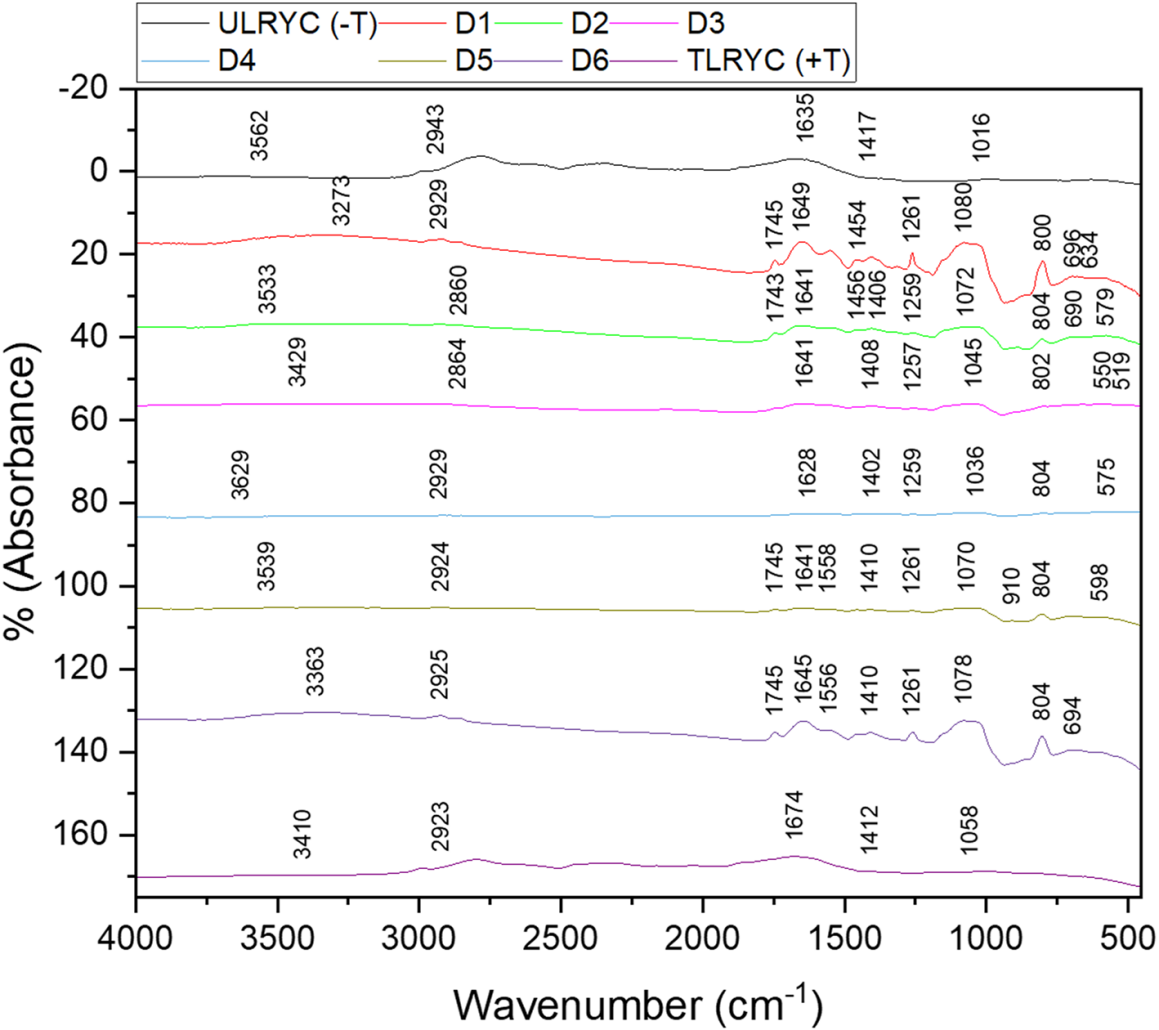
FT-IR Analysis of developed Cd(II) resistant strain *Candida tropicalis* XTA 1874 biomass after desorption.

The infrared spectrum of live yeast cells reveals distinctive vibrational bands and their interactions with Cd^2+^ ions (Fig. 18). The prominent band observed within the wavenumber range of $3500 - 3350 \;{\text{cm}}^{ - 1}$ is attributed to the combined stretching vibrations of N–H and O–H bonds within the live yeast cell structure. The minor peak shifts within this region suggest that Cd^2+^ ions do not exhibit significant mobility towards either free or immobilized cells during the desorption process. Within the region of $2950 - 2900\; {\text{cm}}^{ - 1}$, the observed peaks are associated with the stretching vibrations of –CH bonds. In the $1660 - 1630 \;{\text{cm}}^{ - 1}$ range, C=O stretching vibrations are observed, characterizing untreated live yeast cells. Additionally, peaks within the range of $1265 - 1250 \;{\text{cm}}^{ - 1}$ denote the presence of Cd^2+^ ions within both free and immobilized live yeast cells. The shift in peaks from $1085\,{\text{to}}\; 1010 \;{\text{cm}}^{ - 1}$ signifies the presence of Cd^2+^ ions in both free and Ca-alginate immobilized live yeast cells. A shift from $810\;{\text{to}}\; 690 \;{\text{cm}}^{ - 1}$ in the peak is attributed to the influence of Cd^2+^ ions in the free and immobilized live yeast cells. Lastly, the participation of phosphate groups in the adsorption of Cd^2+^ ions is evident through distinctive peaks in the $590 - 510\;{\text{ cm}}^{ - 1}$ wavenumber range^83^.

## Conclusion

The study shows that after undergoing gradual adaptation to Cd(II) stress, the emerged resistant strain showed considerable changes in surface morphology and effectively binds Cd(II) well from aqueous solution. The data obtained well fitted the Langmuir isotherm model and the adsorption process follows the pseudo second order kinetics. The Cd(II) biosorption process is dependent on the hydrogen ion concentration and maximum sorption took place at $pH$ $6.279 \left( {\sim 6.3} \right)$. Temperature $26.941\;^{ \circ } {\text{C}}$ $\left( {\sim 27\;^{ \circ } {\text{C}}} \right)$, age of inoculum $47.505 \left( {\sim 48\;{\text{h}}} \right)$, volume of inoculum $\left( {8 \times 10^{6} \;{\text{cells}}/{\text{mL}}} \right)$ $4.096 \;{\text{mL }}\left( {4.1 \;{\text{mL}}} \right)$, volume of medium $100.659 \left( {\sim 101\;{\text{mL}}} \right)$, initial Cd(II) concentration $\left( {501\;{\text{ppm}}} \right)$ and dry cell mass $\left( {1.585\;{\text{mg}}/{\text{mL}}} \right)$ also have significant effects on the biosorption process as was obtained by optimization using response surface methodology. Microscopic, Spectroscopic and Diffractometric evidences have shown the incorporation of Cd(II) on the surface as well as intracellular accumulation by the developed resistant strain, *Candida tropicalis* XTA 1874. The strain has shown $87.527 \pm 0.023\%$ desorption capacity at first cycle and acted as an efficient desorbent of Cd(II) upto five cycles.

The obtained results indicate an overall remarkable bioremoval capacity $\left( {75.682 \pm 0.002\% } \right)$ of the strain with respect to Cd(II) under optimum conditions using $500\;{\text{ppm}}$ of initial Cd(II) concentration. Thus in terms of quantitative analysis, biosorption capacity for the newly developed resistant strain has been increased significantly (*p* < 0.0001) from $4.36 \;{\text{ppm}}$ (non-resistant strain) to $378.41 \;{\text{ppm}}$(resistant strain)^65^. In summary, it can be concluded that the strain can be used as an effective biosorbent for Cd(II) removal from aqueous solutions.

### Supplementary Information

Supplementary Information.

## Data Availability

All data generated or analyzed during this study are included in this published article [Supplementary information files].
