# Supplementary material for: Unlocking Cd(II) biosorption potential of Candida tropicalis XTA 1874 for sustainable wastewater treatment
Source: Sci Rep. 2024 Jul 8;14:15690. doi: 10.1038/s41598-024-66336-y (PMC11231346; doi:10.1038/s41598-024-66336-y)
Supplement: Supplementary file 1 — Supplementary Information. [file 41598_2024_66336_MOESM1_ESM.docx]

**Supplementary Table 1. Regression analysis using central composite design (CCD)**

| **Source** | **Sum of Squares** | **df** | **Mean Square** | **F-value** | ***p-value*** |  |
| --- | --- | --- | --- | --- | --- | --- |
| **Model** | 13688.41 | 44 | 311.10 | 49.92 | < 0.0001 | significant |
| A-pH | 3729.33 | 1 | 3729.33 | 598.45 | < 0.0001 |  |
| B-Temperature | 6.50 | 1 | 6.50 | 1.04 | 0.3081 |  |
| C-Age of inoculum | 1.69 | 1 | 1.69 | 0.2707 | 0.6034 |  |
| D-Volume of medium | 0.5782 | 1 | 0.5782 | 0.0928 | 0.7609 |  |
| E-Volume of inoculation | 4.43 | 1 | 4.43 | 0.7102 | 0.4002 |  |
| F-Initial Cd(II) concentration | 3.80 | 1 | 3.80 | 0.6102 | 0.4355 |  |
| G-Contact time | 0.6694 | 1 | 0.6694 | 0.1074 | 0.7434 |  |
| H-Dry Cell weight | 140.16 | 1 | 140.16 | 22.49 | < 0.0001 |  |
| AB | 12.12 | 1 | 12.12 | 1.94 | 0.1645 |  |
| AC | 5.95 | 1 | 5.95 | 0.9544 | 0.3296 |  |
| AD | 0.0619 | 1 | 0.0619 | 0.0099 | 0.9207 |  |
| AE | 1.35 | 1 | 1.35 | 0.2164 | 0.6422 |  |
| AF | 1.563^E-06^ | 1 | 1.563^E-06^ | 2.507E-07 | 0.9996 |  |
| AG | 0.0172 | 1 | 0.0172 | 0.0028 | 0.9581 |  |
| AH | 9.74 | 1 | 9.74 | 1.56 | 0.2124 |  |
| BC | 0.2500 | 1 | 0.2500 | 0.0401 | 0.8414 |  |
| BD | 19.01 | 1 | 19.01 | 3.05 | 0.0820 |  |
| BE | 0.2704 | 1 | 0.2704 | 0.0434 | 0.8352 |  |
| BF | 0.0012 | 1 | 0.0012 | 0.0002 | 0.9888 |  |
| BG | 3.78 | 1 | 3.78 | 0.6071 | 0.4367 |  |
| BH | 10.11 | 1 | 10.11 | 1.62 | 0.2040 |  |
| CD | 0.9120 | 1 | 0.9120 | 0.1464 | 0.7024 |  |
| CE | 5.21 | 1 | 5.21 | 0.8360 | 0.3615 |  |
| CF | 1.64 | 1 | 1.64 | 0.2639 | 0.6079 |  |
| CG | 3.38 | 1 | 3.38 | 0.5418 | 0.4624 |  |
| CH | 0.9025 | 1 | 0.9025 | 0.1448 | 0.7039 |  |
| DE | 1.08 | 1 | 1.08 | 0.1736 | 0.6773 |  |
| DF | 1.11 | 1 | 1.11 | 0.1786 | 0.6730 |  |
| DG | 1.46 | 1 | 1.46 | 0.2340 | 0.6290 |  |
| DH | 1.22 | 1 | 1.22 | 0.1959 | 0.6584 |  |
| EF | 3.48 | 1 | 3.48 | 0.5582 | 0.4557 |  |
| EG | 0.1892 | 1 | 0.1892 | 0.0304 | 0.8618 |  |
| EH | 8.15 | 1 | 8.15 | 1.31 | 0.2539 |  |
| FG | 3.44 | 1 | 3.44 | 0.5522 | 0.4582 |  |
| FH | 1.27 | 1 | 1.27 | 0.2031 | 0.6526 |  |
| GH | 2.62 | 1 | 2.62 | 0.4211 | 0.5170 |  |
| A² | 2905.02 | 1 | 2905.02 | 466.17 | < 0.0001 |  |
| B² | 66.11 | 1 | 66.11 | 10.61 | 0.0013 |  |
| C² | 63.58 | 1 | 63.58 | 10.20 | 0.0016 |  |
| D² | 76.22 | 1 | 76.22 | 12.23 | 0.0006 |  |
| E² | 15.50 | 1 | 15.50 | 2.49 | 0.1161 |  |
| F² | 4.94 | 1 | 4.94 | 0.7923 | 0.3743 |  |
| G² | 3.06 | 1 | 3.06 | 0.4915 | 0.4839 |  |
| H² | 92.72 | 1 | 92.72 | 14.88 | 0.0001 |  |
| **Residual** | 1476.91 | 237 | 6.23 |  |  |  |
| Lack of Fit | 1373.52 | 228 | 6.02 | 0.5244 | 0.9480 | not significant |
| Pure Error | 103.39 | 9 | 11.49 |  |  |  |
| **Cor Total** | 15165.32 | 281 |  |  |  |  |

Factor coding is **Coded**.
Sum of squares is **Type III - Partial**

The **Model F-value** of 49.92 implies the model is significant. There is only a 0.01% chance that an F-value this large could occur due to noise.

**P-values** less than 0.0500 indicate model terms are significant. In this case A, H, A², B², C², D², H² are significant model terms. Values greater than 0.1000 indicate the model terms are not significant. If there are many insignificant model terms (not counting those required to support hierarchy), model reduction may improve your model.

The **Lack of Fit F-value** of 0.52 implies the Lack of Fit is not significant relative to the pure error. There is a 94.80% chance that a Lack of Fit F-value this large could occur due to noise. Non-significant lack of fit is good -- we want the model to fit.

**Fit Statistics**

| **Std. Dev.** | 2.50 |  | **R²** | 0.9026 |
| --- | --- | --- | --- | --- |
| **Mean** | 52.70 |  | **Adjusted R²** | 0.8845 |
| **C.V. %** | 4.74 |  | **Predicted R²** | 0.8126 |
|  |  |  | **Adeq Precision** | 42.4513 |

The **Predicted R²** of 0.8126 is in reasonable agreement with the **Adjusted R²** of 0.8845; i.e. the difference is less than 0.2.

**Adeq Precision** measures the signal to noise ratio. A ratio greater than 4 is desirable. Your ratio of 42.451 indicates an adequate signal. This model can be used to navigate the design space.

**Supplementary Table 2. Experimental design based on Central Composite Design (CCD)**

| **Independent Variables** | | | |  |  |  |  |  | **Response** | |  |  |
| --- | --- | --- | --- | --- | --- | --- | --- | --- | --- | --- | --- | --- |
| Run | A:pH | \| B:Temperature \| \| --- \| | C:Age of inoculum | D:Volume of medium | E:Volume of inoculation | \| F:Intial Cd(II) concentration \| \| --- \| | G:Contact time | H:Dry Cell weight | \| Actual value \| \| --- \| | Predicted value | Residual |  |
|  |  | ℃ | h | mL | 8×10^6^ cells/mL | ppm | min | mg/mL |  |  |  |  |
| 1 | 8 | 30 | 24 | 150 | 3 | 400 | 200 | 1 | 48.36 | 45.26 | 3.1 |  |
| 2 | 5 | 30 | 72 | 150 | 5 | 400 | 200 | 2 | 56.39 | 55 | 1.39 |  |
| 3 | 8 | 30 | 24 | 50 | 5 | 600 | 200 | 1 | 46.21 | 46.29 | -0.0837 |  |
| 4 | 5 | 24 | 72 | 150 | 3 | 600 | 100 | 1 | 52.41 | 53.67 | -1.26 |  |
| 5 | 5 | 24 | 72 | 50 | 5 | 600 | 100 | 1 | 50.34 | 53.48 | -3.14 |  |
| 6 | 5 | 30 | 24 | 50 | 3 | 600 | 200 | 1 | 52.66 | 54.17 | -1.51 |  |
| 7 | 5 | 30 | 24 | 150 | 3 | 600 | 200 | 2 | 56.38 | 56.12 | 0.2609 |  |
| 8 | 8 | 30 | 72 | 150 | 5 | 600 | 200 | 1 | 46.25 | 46.67 | -0.4227 |  |
| 9 | 8 | 30 | 72 | 150 | 3 | 400 | 200 | 1 | 46.3 | 45.13 | 1.17 |  |
| 10 | 8 | 24 | 72 | 50 | 3 | 600 | 200 | 2 | 48.25 | 48.56 | -0.3124 |  |
| 11 | 8 | 30 | 72 | 150 | 5 | 400 | 100 | 1 | 46.21 | 46.88 | -0.6722 |  |
| 12 | 8 | 24 | 24 | 50 | 3 | 400 | 200 | 2 | 48.67 | 47.43 | 1.24 |  |
| 13 | 8 | 24 | 24 | 50 | 5 | 600 | 200 | 1 | 48.97 | 47.02 | 1.95 |  |
| 14 | 6.5 | 27 | 48 | 100 | 4 | 500 | 150 | 1.5 | 72.56 | 73.78 | -1.22 |  |
| 15 | 8 | 24 | 24 | 50 | 3 | 400 | 200 | 1 | 46.37 | 46.48 | -0.1107 |  |
| 16 | 5 | 30 | 24 | 150 | 5 | 400 | 100 | 2 | 57.01 | 56.65 | 0.3643 |  |
| 17 | 5 | 30 | 72 | 50 | 3 | 400 | 100 | 2 | 56.78 | 55.77 | 1.01 |  |
| 18 | 5 | 24 | 24 | 50 | 3 | 600 | 100 | 2 | 56.12 | 56.03 | 0.0924 |  |
| 19 | 8 | 24 | 72 | 50 | 3 | 400 | 200 | 2 | 47.98 | 47.42 | 0.5611 |  |
| 20 | 5 | 30 | 24 | 50 | 3 | 400 | 100 | 2 | 58.74 | 56.98 | 1.76 |  |
| 21 | 5 | 30 | 24 | 50 | 5 | 600 | 100 | 2 | 56.03 | 56.56 | -0.5253 |  |
| 22 | 5 | 30 | 72 | 50 | 5 | 400 | 200 | 2 | 56.21 | 55.57 | 0.6393 |  |
| 23 | 5 | 24 | 72 | 50 | 5 | 600 | 100 | 2 | 56.32 | 54.94 | 1.38 |  |
| 24 | 5 | 30 | 72 | 50 | 5 | 600 | 200 | 2 | 58.21 | 56.24 | 1.97 |  |
| 25 | 8 | 24 | 24 | 150 | 3 | 400 | 100 | 2 | 47.84 | 48.47 | -0.6298 |  |
| 26 | 8 | 24 | 72 | 50 | 5 | 600 | 100 | 1 | 48.75 | 47.22 | 1.53 |  |
| 27 | 5 | 24 | 72 | 50 | 3 | 400 | 100 | 1 | 52.31 | 52.7 | -0.3867 |  |
| 28 | 8 | 30 | 72 | 50 | 5 | 400 | 200 | 1 | 46.34 | 46.9 | -0.5617 |  |
| 29 | 8 | 24 | 72 | 150 | 5 | 600 | 100 | 2 | 48.32 | 48.4 | -0.0791 |  |
| 30 | 5 | 30 | 24 | 50 | 3 | 600 | 200 | 2 | 56.34 | 56.97 | -0.6306 |  |
| 31 | 8 | 30 | 72 | 50 | 3 | 400 | 100 | 1 | 46.21 | 45.66 | 0.5473 |  |
| 32 | 6.5 | 27 | 48 | 100 | 4 | 500 | 234.09 | 1.5 | 70.24 | 72.53 | -2.29 |  |
| 33 | 5 | 24 | 72 | 150 | 5 | 400 | 100 | 2 | 56.47 | 55.55 | 0.9174 |  |
| 34 | 6.5 | 27 | 7.637 | 100 | 4 | 500 | 150 | 1.5 | 68.75 | 68.59 | 0.1569 |  |
| 35 | 8 | 24 | 72 | 50 | 5 | 600 | 200 | 1 | 48.96 | 48.14 | 0.8232 |  |
| 36 | 5 | 30 | 24 | 150 | 3 | 400 | 100 | 1 | 56.74 | 54.04 | 2.7 |  |
| 37 | 8 | 24 | 24 | 150 | 3 | 400 | 200 | 1 | 46.87 | 47.2 | -0.3271 |  |
| 38 | 8 | 30 | 72 | 50 | 3 | 600 | 100 | 2 | 48.75 | 48.24 | 0.5071 |  |
| 39 | 5 | 24 | 24 | 150 | 5 | 600 | 200 | 2 | 56.87 | 55.65 | 1.22 |  |
| 40 | 8 | 24 | 24 | 50 | 5 | 600 | 100 | 1 | 46.98 | 46.56 | 0.423 |  |
| 41 | 8 | 30 | 72 | 50 | 5 | 400 | 100 | 2 | 46.85 | 48.13 | -1.28 |  |
| 42 | 8 | 24 | 24 | 150 | 3 | 400 | 100 | 1 | 48.04 | 47.39 | 0.6457 |  |
| 43 | 5 | 30 | 72 | 150 | 3 | 600 | 100 | 2 | 56.87 | 55.66 | 1.21 |  |
| 44 | 5 | 24 | 72 | 50 | 5 | 400 | 200 | 2 | 54.21 | 54.74 | -0.5346 |  |
| 45 | 8 | 24 | 24 | 50 | 3 | 600 | 100 | 1 | 46.97 | 46.45 | 0.5161 |  |
| 46 | 5 | 30 | 24 | 50 | 5 | 400 | 100 | 1 | 52.36 | 54.46 | -2.1 |  |
| 47 | 8 | 30 | 72 | 150 | 3 | 600 | 200 | 2 | 47.69 | 47.23 | 0.463 |  |
| 48 | 5 | 24 | 72 | 50 | 3 | 600 | 100 | 1 | 52.64 | 53.09 | -0.4548 |  |
| 49 | 5 | 24 | 72 | 50 | 5 | 600 | 200 | 2 | 54.02 | 55.42 | -1.4 |  |
| 50 | 5 | 24 | 72 | 50 | 5 | 400 | 100 | 2 | 56.34 | 54.73 | 1.61 |  |
| 51 | 6.5 | 27 | 48 | 100 | 4 | 500 | 150 | 2.341 | 75.63 | 68.58 | 7.05 |  |
| 52 | 8 | 24 | 24 | 150 | 5 | 600 | 200 | 1 | 46.09 | 47.73 | -1.64 |  |
| 53 | 6.5 | 27 | 48 | 100 | 4 | 500 | 150 | 1.5 | 77.87 | 73.78 | 4.09 |  |
| 54 | 8 | 30 | 24 | 50 | 5 | 600 | 100 | 1 | 48.27 | 46.32 | 1.95 |  |
| 55 | 5 | 30 | 72 | 150 | 5 | 400 | 200 | 1 | 52.36 | 53.71 | -1.35 |  |
| 56 | 8 | 24 | 72 | 150 | 5 | 600 | 100 | 1 | 44.25 | 47.99 | -3.74 |  |
| 57 | 5 | 24 | 24 | 150 | 3 | 600 | 100 | 2 | 60.32 | 56.57 | 3.75 |  |
| 58 | 5 | 30 | 72 | 150 | 5 | 400 | 100 | 1 | 52.31 | 54.08 | -1.77 |  |
| 59 | 6.5 | 27 | 48 | 15.9104 | 4 | 500 | 150 | 1.5 | 64.74 | 67.87 | -3.13 |  |
| 60 | 5 | 30 | 24 | 150 | 5 | 600 | 200 | 2 | 54.21 | 55.5 | -1.29 |  |
| 61 | 5 | 30 | 72 | 50 | 3 | 400 | 200 | 2 | 54.07 | 55.41 | -1.34 |  |
| 62 | 5 | 24 | 72 | 150 | 5 | 600 | 200 | 2 | 58.74 | 55.68 | 3.06 |  |
| 63 | 8 | 30 | 72 | 50 | 5 | 400 | 100 | 1 | 48.63 | 46.93 | 1.7 |  |
| 64 | 8 | 24 | 72 | 150 | 5 | 400 | 200 | 1 | 48.03 | 48.48 | -0.4485 |  |
| 65 | 5 | 24 | 24 | 50 | 5 | 400 | 100 | 2 | 54.37 | 55.23 | -0.8642 |  |
| 66 | 8 | 30 | 72 | 150 | 3 | 400 | 100 | 2 | 50.31 | 46.99 | 3.32 |  |
| 67 | 8 | 30 | 24 | 50 | 5 | 400 | 200 | 1 | 46.35 | 46.23 | 0.1229 |  |
| 68 | 8 | 24 | 72 | 150 | 5 | 600 | 200 | 2 | 46.02 | 48.61 | -2.59 |  |
| 69 | 5 | 24 | 24 | 50 | 3 | 600 | 100 | 1 | 52.36 | 53.61 | -1.25 |  |
| 70 | 5 | 30 | 24 | 150 | 5 | 400 | 200 | 1 | 46.51 | 53.88 | -7.37 |  |
| 71 | 8 | 30 | 24 | 150 | 3 | 600 | 100 | 1 | 46.32 | 45.75 | 0.5683 |  |
| 72 | 8 | 24 | 24 | 50 | 5 | 400 | 200 | 1 | 46.55 | 46.94 | -0.3913 |  |
| 73 | 8 | 30 | 72 | 50 | 3 | 600 | 200 | 2 | 44.24 | 48.38 | -4.14 |  |
| 74 | 8 | 24 | 24 | 50 | 5 | 400 | 200 | 2 | 46.38 | 47.17 | -0.7943 |  |
| 75 | 5 | 30 | 72 | 50 | 5 | 600 | 100 | 1 | 52.36 | 53.99 | -1.63 |  |
| 76 | 8 | 30 | 24 | 150 | 5 | 400 | 200 | 1 | 44.07 | 46.11 | -2.04 |  |
| 77 | 5 | 30 | 24 | 50 | 5 | 600 | 200 | 2 | 56.07 | 56.09 | -0.0208 |  |
| 78 | 8 | 30 | 72 | 50 | 5 | 600 | 100 | 1 | 46.55 | 46.86 | -0.3061 |  |
| 79 | 5 | 30 | 24 | 150 | 5 | 400 | 200 | 2 | 58.78 | 55.42 | 3.36 |  |
| 80 | 8 | 30 | 72 | 50 | 3 | 400 | 200 | 1 | 44.23 | 45.74 | -1.51 |  |
| 81 | 6.5 | 27 | 48 | 100 | 4 | 500 | 150 | 1.5 | 69.02 | 73.78 | -4.76 |  |
| 82 | 5 | 30 | 72 | 150 | 3 | 600 | 200 | 1 | 52.47 | 53.17 | -0.6982 |  |
| 83 | 5 | 24 | 72 | 150 | 3 | 600 | 200 | 1 | 52.07 | 54.37 | -2.3 |  |
| 84 | 5 | 30 | 72 | 150 | 5 | 600 | 200 | 2 | 56.25 | 55.41 | 0.8414 |  |
| 85 | 8 | 30 | 72 | 150 | 3 | 400 | 200 | 2 | 48.74 | 46.36 | 2.38 |  |
| 86 | 8 | 24 | 72 | 50 | 3 | 600 | 100 | 2 | 48.24 | 47.94 | 0.3004 |  |
| 87 | 8 | 30 | 24 | 50 | 5 | 400 | 100 | 2 | 48.93 | 48.15 | 0.7795 |  |
| 88 | 8 | 24 | 72 | 150 | 3 | 600 | 200 | 2 | 48.01 | 48.5 | -0.49 |  |
| 89 | 5 | 24 | 24 | 50 | 5 | 400 | 100 | 1 | 52.31 | 53.82 | -1.51 |  |
| 90 | 5 | 30 | 24 | 150 | 3 | 600 | 100 | 1 | 52.64 | 53.84 | -1.2 |  |
| 91 | 6.5 | 27 | 48 | 100 | 5.6818 | 500 | 150 | 1.5 | 69.37 | 71.37 | -2 |  |
| 92 | 8 | 24 | 24 | 50 | 3 | 400 | 100 | 2 | 48.21 | 47.73 | 0.4821 |  |
| 93 | 6.5 | 27 | 48 | 100 | 4 | 500 | 150 | 1.5 | 70.23 | 73.78 | -3.55 |  |
| 94 | 5 | 24 | 24 | 50 | 3 | 400 | 100 | 2 | 54.89 | 55.67 | -0.7789 |  |
| 95 | 8 | 30 | 72 | 150 | 5 | 600 | 100 | 2 | 48.21 | 47.74 | 0.4676 |  |
| 96 | 5 | 30 | 24 | 50 | 3 | 400 | 200 | 2 | 54.07 | 56.16 | -2.09 |  |
| 97 | 8 | 30 | 24 | 50 | 5 | 400 | 200 | 2 | 48 | 47.26 | 0.7449 |  |
| 98 | 5 | 24 | 72 | 50 | 5 | 600 | 200 | 1 | 52.41 | 54.36 | -1.95 |  |
| 99 | 8 | 24 | 24 | 150 | 5 | 400 | 100 | 2 | 48.96 | 48.59 | 0.3746 |  |
| 100 | 8 | 24 | 72 | 50 | 5 | 600 | 200 | 2 | 48.78 | 48.41 | 0.3664 |  |
| 101 | 8 | 24 | 24 | 150 | 5 | 400 | 100 | 1 | 46.41 | 48.22 | -1.81 |  |
| 102 | 8 | 30 | 72 | 150 | 3 | 600 | 200 | 1 | 46.05 | 45.72 | 0.3322 |  |
| 103 | 5 | 24 | 72 | 50 | 3 | 400 | 100 | 2 | 56.57 | 54.59 | 1.98 |  |
| 104 | 6.5 | 32.05 | 48 | 100 | 4 | 500 | 150 | 1.5 | 71.03 | 68.09 | 2.94 |  |
| 105 | 5 | 30 | 24 | 50 | 5 | 600 | 200 | 1 | 54.27 | 54 | 0.2687 |  |
| 106 | 5 | 24 | 24 | 50 | 3 | 400 | 100 | 1 | 54.74 | 53.54 | 1.2 |  |
| 107 | 5 | 30 | 24 | 150 | 3 | 600 | 200 | 1 | 54.21 | 53.59 | 0.618 |  |
| 108 | 5 | 30 | 72 | 50 | 3 | 600 | 200 | 1 | 53.69 | 53.98 | -0.2921 |  |
| 109 | 5 | 30 | 72 | 150 | 3 | 600 | 100 | 1 | 52 | 52.96 | -0.9614 |  |
| 110 | 8 | 24 | 72 | 50 | 3 | 400 | 100 | 1 | 44.32 | 46.15 | -1.83 |  |
| 111 | 8 | 24 | 72 | 50 | 3 | 600 | 200 | 1 | 48.01 | 47.57 | 0.4382 |  |
| 112 | 6.5 | 27 | 88.363 | 100 | 4 | 500 | 150 | 1.5 | 71.23 | 68.32 | 2.91 |  |
| 113 | 5 | 30 | 24 | 50 | 5 | 400 | 200 | 1 | 58.9 | 53.93 | 4.97 |  |
| 114 | 5 | 24 | 72 | 150 | 5 | 400 | 200 | 2 | 46.74 | 55.27 | -8.53 |  |
| 115 | 8 | 24 | 72 | 150 | 3 | 400 | 100 | 1 | 44.71 | 46.93 | -2.22 |  |
| 116 | 8 | 24 | 72 | 150 | 5 | 400 | 100 | 1 | 58.63 | 48.33 | 10.3 |  |
| 117 | 5 | 30 | 72 | 150 | 5 | 600 | 200 | 1 | 56.34 | 53.83 | 2.51 |  |
| 118 | 8 | 30 | 72 | 50 | 3 | 400 | 200 | 2 | 48.12 | 47.24 | 0.8753 |  |
| 119 | 5 | 24 | 24 | 150 | 5 | 400 | 100 | 1 | 58.09 | 55.16 | 2.93 |  |
| 120 | 8 | 24 | 24 | 150 | 5 | 600 | 100 | 2 | 46.36 | 48.21 | -1.85 |  |
| 121 | 6.5 | 27 | 48 | 100 | 4 | 500 | 65.9104 | 1.5 | 78.05 | 72.7 | 5.35 |  |
| 122 | 8 | 24 | 72 | 150 | 5 | 400 | 100 | 2 | 48.75 | 48.45 | 0.3006 |  |
| 123 | 8 | 24 | 24 | 50 | 3 | 600 | 100 | 2 | 48.75 | 48.09 | 0.6631 |  |
| 124 | 8 | 24 | 24 | 50 | 3 | 600 | 200 | 1 | 46.97 | 47.02 | -0.0523 |  |
| 125 | 5 | 24 | 24 | 150 | 3 | 400 | 200 | 2 | 56.34 | 55.84 | 0.502 |  |
| 126 | 5 | 24 | 72 | 150 | 5 | 600 | 100 | 2 | 54.12 | 55.5 | -1.38 |  |
| 127 | 3.98 | 27 | 48 | 100 | 4 | 500 | 150 | 1.5 | 36.52 | 44.15 | -7.63 |  |
| 128 | 8 | 24 | 24 | 50 | 5 | 400 | 100 | 1 | 46.07 | 46.95 | -0.8754 |  |
| 129 | 8 | 30 | 24 | 150 | 3 | 400 | 200 | 2 | 48.99 | 46.73 | 2.26 |  |
| 130 | 5 | 24 | 72 | 150 | 3 | 400 | 100 | 2 | 56.34 | 55.16 | 1.18 |  |
| 131 | 5 | 24 | 72 | 150 | 3 | 400 | 200 | 2 | 54.74 | 54.98 | -0.241 |  |
| 132 | 8 | 24 | 72 | 50 | 5 | 400 | 200 | 2 | 48.07 | 47.74 | 0.3336 |  |
| 133 | 8 | 30 | 72 | 150 | 3 | 600 | 100 | 1 | 44.01 | 45.48 | -1.47 |  |
| 134 | 8 | 30 | 24 | 50 | 3 | 600 | 100 | 1 | 48.21 | 46.09 | 2.12 |  |
| 135 | 5 | 24 | 72 | 50 | 5 | 400 | 200 | 1 | 58.79 | 53.97 | 4.82 |  |
| 136 | 8 | 24 | 72 | 150 | 3 | 400 | 100 | 2 | 44.36 | 47.76 | -3.4 |  |
| 137 | 8 | 30 | 24 | 50 | 3 | 400 | 100 | 1 | 42.98 | 46.02 | -3.04 |  |
| 138 | 8 | 24 | 24 | 50 | 5 | 600 | 200 | 2 | 48.24 | 47.53 | 0.7091 |  |
| 139 | 5 | 24 | 24 | 150 | 3 | 600 | 200 | 1 | 56.34 | 54.66 | 1.68 |  |
| 140 | 8 | 30 | 24 | 50 | 5 | 400 | 100 | 1 | 46.32 | 46.72 | -0.3975 |  |
| 141 | 6.5 | 27 | 48 | 100 | 4 | 500 | 150 | 0.659 | 62.14 | 66.12 | -3.98 |  |
| 142 | 8 | 24 | 72 | 50 | 3 | 400 | 200 | 1 | 46.96 | 46.71 | 0.2503 |  |
| 143 | 8 | 24 | 24 | 150 | 3 | 600 | 100 | 1 | 47.28 | 47.21 | 0.0716 |  |
| 144 | 5 | 24 | 72 | 50 | 5 | 400 | 100 | 1 | 54 | 53.55 | 0.4537 |  |
| 145 | 5 | 30 | 72 | 150 | 3 | 400 | 200 | 1 | 54.12 | 52.58 | 1.54 |  |
| 146 | 5 | 30 | 24 | 150 | 3 | 400 | 200 | 2 | 56.37 | 55.57 | 0.8009 |  |
| 147 | 8 | 30 | 24 | 50 | 3 | 600 | 200 | 1 | 46.37 | 46.17 | 0.2007 |  |
| 148 | 5 | 24 | 24 | 50 | 5 | 600 | 100 | 2 | 52.69 | 55.13 | -2.44 |  |
| 149 | 8 | 24 | 24 | 50 | 5 | 600 | 100 | 2 | 48.15 | 47.48 | 0.6737 |  |
| 150 | 5 | 24 | 72 | 50 | 3 | 600 | 200 | 2 | 56.34 | 55.86 | 0.4794 |  |
| 151 | 5 | 24 | 72 | 50 | 3 | 400 | 200 | 1 | 54.39 | 53.23 | 1.16 |  |
| 152 | 6.5 | 27 | 48 | 100 | 4 | 500 | 150 | 1.5 | 78.12 | 73.78 | 4.34 |  |
| 153 | 8 | 24 | 72 | 150 | 3 | 600 | 200 | 1 | 46.34 | 47.79 | -1.45 |  |
| 154 | 8 | 24 | 24 | 150 | 5 | 400 | 200 | 2 | 48.04 | 47.87 | 0.1656 |  |
| 155 | 5 | 30 | 72 | 50 | 5 | 400 | 100 | 2 | 56.32 | 56.04 | 0.2805 |  |
| 156 | 5 | 30 | 72 | 50 | 5 | 400 | 200 | 1 | 56.47 | 54 | 2.47 |  |
| 157 | 6.5 | 27 | 48 | 100 | 4 | 500 | 150 | 1.5 | 70.12 | 73.78 | -3.66 |  |
| 158 | 5 | 30 | 72 | 150 | 3 | 600 | 200 | 2 | 56.37 | 55.46 | 0.9122 |  |
| 159 | 8 | 30 | 72 | 50 | 5 | 600 | 200 | 2 | 46.32 | 48.36 | -2.04 |  |
| 160 | 8 | 30 | 24 | 150 | 5 | 600 | 200 | 1 | 44.23 | 45.92 | -1.69 |  |
| 161 | 5 | 24 | 24 | 50 | 5 | 400 | 200 | 2 | 56.34 | 54.79 | 1.55 |  |
| 162 | 5 | 24 | 24 | 150 | 3 | 400 | 200 | 1 | 56.32 | 54.39 | 1.93 |  |
| 163 | 5 | 24 | 24 | 50 | 3 | 600 | 200 | 2 | 58.96 | 56.16 | 2.8 |  |
| 164 | 8 | 24 | 72 | 150 | 3 | 400 | 200 | 1 | 46.74 | 47.19 | -0.4473 |  |
| 165 | 5 | 24 | 72 | 150 | 5 | 600 | 100 | 1 | 54.32 | 54.32 | 0.0039 |  |
| 166 | 5 | 30 | 24 | 50 | 5 | 400 | 200 | 2 | 54.07 | 55.74 | -1.67 |  |
| 167 | 5 | 24 | 24 | 150 | 5 | 600 | 200 | 1 | 54.01 | 54.63 | -0.6187 |  |
| 168 | 8 | 24 | 72 | 150 | 3 | 400 | 200 | 2 | 48.01 | 47.62 | 0.3897 |  |
| 169 | 5 | 24 | 24 | 50 | 3 | 400 | 200 | 1 | 54.78 | 53.61 | 1.17 |  |
| 170 | 5 | 24 | 24 | 150 | 5 | 400 | 100 | 2 | 56.87 | 56.3 | 0.5717 |  |
| 171 | 5 | 24 | 24 | 150 | 5 | 400 | 200 | 2 | 58.41 | 55.55 | 2.86 |  |
| 172 | 5 | 24 | 24 | 50 | 5 | 600 | 200 | 2 | 54.12 | 55.15 | -1.03 |  |
| 173 | 5 | 24 | 72 | 150 | 3 | 600 | 200 | 2 | 56.34 | 55.86 | 0.4796 |  |
| 174 | 8 | 30 | 24 | 50 | 5 | 600 | 200 | 2 | 48.76 | 47.6 | 1.16 |  |
| 175 | 5 | 30 | 72 | 50 | 3 | 600 | 100 | 1 | 54.12 | 53.47 | 0.6466 |  |
| 176 | 6.5 | 27 | 48 | 100 | 2.3182 | 500 | 150 | 1.5 | 76 | 70.93 | 5.07 |  |
| 177 | 8 | 30 | 24 | 150 | 5 | 600 | 100 | 1 | 46.32 | 46.24 | 0.0752 |  |
| 178 | 5 | 30 | 24 | 50 | 3 | 600 | 100 | 2 | 56.24 | 57.33 | -1.09 |  |
| 179 | 8 | 30 | 72 | 50 | 5 | 400 | 200 | 2 | 48.76 | 47.69 | 1.07 |  |
| 180 | 5 | 30 | 72 | 150 | 5 | 400 | 100 | 2 | 56.97 | 55.77 | 1.2 |  |
| 181 | 8 | 30 | 24 | 150 | 5 | 600 | 100 | 2 | 47.87 | 47.68 | 0.1872 |  |
| 182 | 5 | 24 | 72 | 50 | 3 | 600 | 100 | 2 | 56.37 | 55.27 | 1.1 |  |
| 183 | 5 | 30 | 24 | 50 | 5 | 600 | 100 | 1 | 58.97 | 54.06 | 4.91 |  |
| 184 | 5 | 24 | 72 | 150 | 5 | 400 | 100 | 1 | 54.94 | 54.65 | 0.292 |  |
| 185 | 6.5 | 27 | 48 | 100 | 4 | 500 | 150 | 1.5 | 72.04 | 73.78 | -1.74 |  |
| 186 | 5 | 24 | 72 | 150 | 5 | 600 | 200 | 1 | 54.21 | 54.9 | -0.6904 |  |
| 187 | 5 | 24 | 72 | 150 | 3 | 400 | 200 | 1 | 56.07 | 53.77 | 2.3 |  |
| 188 | 5 | 30 | 24 | 50 | 3 | 400 | 100 | 1 | 52.03 | 54.05 | -2.02 |  |
| 189 | 8 | 30 | 24 | 150 | 5 | 400 | 200 | 2 | 48.75 | 46.87 | 1.88 |  |
| 190 | 6.5 | 27 | 48 | 184.09 | 4 | 500 | 150 | 1.5 | 74.23 | 68.03 | 6.2 |  |
| 191 | 8 | 30 | 24 | 50 | 3 | 400 | 200 | 1 | 44.57 | 45.64 | -1.07 |  |
| 192 | 8 | 30 | 24 | 150 | 5 | 600 | 200 | 2 | 47.04 | 46.95 | 0.0907 |  |
| 193 | 5 | 24 | 24 | 150 | 3 | 600 | 200 | 2 | 56.78 | 56.4 | 0.3832 |  |
| 194 | 5 | 24 | 24 | 50 | 3 | 600 | 200 | 1 | 50.42 | 54.15 | -3.73 |  |
| 195 | 5 | 30 | 72 | 50 | 3 | 400 | 200 | 1 | 52.36 | 53.13 | -0.769 |  |
| 196 | 5 | 24 | 24 | 50 | 5 | 600 | 200 | 1 | 52.47 | 53.85 | -1.38 |  |
| 197 | 5 | 30 | 72 | 50 | 3 | 400 | 100 | 1 | 52.74 | 53.08 | -0.3441 |  |
| 198 | 8 | 24 | 72 | 50 | 5 | 600 | 100 | 2 | 46.35 | 47.9 | -1.55 |  |
| 199 | 9.02 | 27 | 48 | 100 | 4 | 500 | 150 | 1.5 | 42.14 | 31.45 | 10.69 |  |
| 200 | 8 | 30 | 24 | 50 | 3 | 400 | 200 | 2 | 48.06 | 47.38 | 0.6817 |  |
| 201 | 8 | 30 | 72 | 50 | 3 | 600 | 200 | 1 | 46.46 | 46.59 | -0.1339 |  |
| 202 | 8 | 24 | 72 | 150 | 5 | 400 | 200 | 2 | 48 | 48.2 | -0.1977 |  |
| 203 | 5 | 30 | 72 | 50 | 3 | 600 | 200 | 2 | 58.36 | 56.55 | 1.81 |  |
| 204 | 5 | 24 | 24 | 50 | 3 | 400 | 200 | 2 | 54.63 | 55.34 | -0.7057 |  |
| 205 | 8 | 24 | 24 | 150 | 5 | 600 | 100 | 1 | 46.37 | 47.57 | -1.2 |  |
| 206 | 5 | 30 | 24 | 150 | 5 | 400 | 100 | 1 | 54.21 | 54.71 | -0.4986 |  |
| 207 | 5 | 24 | 72 | 150 | 3 | 400 | 100 | 1 | 52.36 | 53.54 | -1.18 |  |
| 208 | 5 | 30 | 72 | 50 | 5 | 600 | 200 | 1 | 55.34 | 54.39 | 0.9532 |  |
| 209 | 8 | 30 | 24 | 150 | 3 | 600 | 200 | 2 | 44.27 | 47.28 | -3.01 |  |
| 210 | 5 | 24 | 24 | 50 | 5 | 400 | 200 | 1 | 54.74 | 53.78 | 0.9611 |  |
| 211 | 6.5 | 27 | 48 | 100 | 4 | 668.179 | 150 | 1.5 | 76.34 | 72.5 | 3.84 |  |
| 212 | 5 | 30 | 72 | 50 | 5 | 400 | 100 | 1 | 54.62 | 54.06 | 0.5563 |  |
| 213 | 8 | 24 | 24 | 50 | 3 | 400 | 100 | 1 | 46.38 | 46.38 | 0.0039 |  |
| 214 | 8 | 30 | 24 | 50 | 3 | 400 | 100 | 2 | 47.78 | 48.16 | -0.3849 |  |
| 215 | 8 | 30 | 72 | 150 | 5 | 600 | 200 | 2 | 47.58 | 47.47 | 0.1118 |  |
| 216 | 8 | 30 | 72 | 50 | 3 | 400 | 100 | 2 | 46.37 | 47.57 | -1.2 |  |
| 217 | 6.5 | 21.95 | 48 | 100 | 4 | 500 | 150 | 1.5 | 68.74 | 68.62 | 0.1218 |  |
| 218 | 5 | 24 | 24 | 150 | 5 | 600 | 100 | 1 | 56.14 | 54.5 | 1.64 |  |
| 219 | 6.5 | 27 | 48 | 100 | 4 | 500 | 150 | 1.5 | 72.01 | 73.78 | -1.77 |  |
| 220 | 8 | 30 | 24 | 150 | 3 | 600 | 200 | 1 | 45.5 | 45.53 | -0.0319 |  |
| 221 | 8 | 30 | 72 | 50 | 5 | 600 | 200 | 1 | 46.38 | 47.29 | -0.9089 |  |
| 222 | 5 | 30 | 24 | 150 | 3 | 400 | 200 | 1 | 54.63 | 53.32 | 1.31 |  |
| 223 | 5 | 30 | 24 | 150 | 3 | 400 | 100 | 2 | 56.78 | 56.69 | 0.0896 |  |
| 224 | 8 | 30 | 72 | 50 | 3 | 600 | 100 | 1 | 46.87 | 46.05 | 0.8176 |  |
| 225 | 5 | 30 | 24 | 50 | 5 | 400 | 100 | 2 | 56.24 | 56.67 | -0.4315 |  |
| 226 | 8 | 30 | 24 | 150 | 3 | 400 | 100 | 2 | 44.28 | 47.82 | -3.54 |  |
| 227 | 5 | 30 | 72 | 150 | 5 | 600 | 100 | 1 | 54.74 | 53.73 | 1.01 |  |
| 228 | 8 | 24 | 24 | 150 | 5 | 400 | 200 | 1 | 46.35 | 47.92 | -1.57 |  |
| 229 | 8 | 24 | 24 | 150 | 3 | 400 | 200 | 2 | 44.07 | 47.87 | -3.8 |  |
| 230 | 8 | 30 | 24 | 50 | 3 | 600 | 200 | 2 | 47.25 | 48.19 | -0.9424 |  |
| 231 | 8 | 30 | 24 | 150 | 3 | 600 | 100 | 2 | 46.35 | 47.9 | -1.55 |  |
| 232 | 5 | 24 | 24 | 150 | 3 | 600 | 100 | 1 | 54.69 | 54.43 | 0.259 |  |
| 233 | 5 | 30 | 72 | 150 | 3 | 400 | 100 | 1 | 54.78 | 52.84 | 1.94 |  |
| 234 | 8 | 24 | 72 | 50 | 3 | 600 | 100 | 1 | 46.34 | 46.54 | -0.2041 |  |
| 235 | 8 | 30 | 24 | 150 | 5 | 400 | 100 | 1 | 46.34 | 46.91 | -0.5657 |  |
| 236 | 8 | 24 | 24 | 50 | 3 | 600 | 200 | 2 | 48.03 | 48.25 | -0.2203 |  |
| 237 | 8 | 30 | 24 | 50 | 3 | 600 | 100 | 2 | 48.21 | 48.52 | -0.3052 |  |
| 238 | 5 | 24 | 24 | 150 | 5 | 400 | 200 | 1 | 54.69 | 54.82 | -0.1274 |  |
| 239 | 5 | 30 | 72 | 150 | 3 | 400 | 100 | 2 | 54.63 | 55.25 | -0.6191 |  |
| 240 | 8 | 24 | 72 | 150 | 3 | 600 | 100 | 2 | 47.87 | 48.18 | -0.3091 |  |
| 241 | 5 | 24 | 72 | 150 | 3 | 600 | 100 | 2 | 54.74 | 55.57 | -0.8323 |  |
| 242 | 5 | 24 | 24 | 150 | 5 | 600 | 100 | 2 | 56.14 | 55.93 | 0.2129 |  |
| 243 | 5 | 24 | 72 | 50 | 3 | 400 | 200 | 2 | 54.31 | 54.72 | -0.4075 |  |
| 244 | 5 | 24 | 24 | 150 | 3 | 400 | 100 | 2 | 54.01 | 56.47 | -2.46 |  |
| 245 | 5 | 24 | 72 | 50 | 3 | 600 | 200 | 1 | 56.32 | 54.09 | 2.23 |  |
| 246 | 8 | 30 | 24 | 150 | 5 | 400 | 100 | 2 | 48.74 | 48.06 | 0.6775 |  |
| 247 | 5 | 30 | 72 | 150 | 5 | 600 | 100 | 2 | 56.34 | 55.72 | 0.6244 |  |
| 248 | 5 | 30 | 72 | 150 | 3 | 400 | 200 | 2 | 48.12 | 54.59 | -6.47 |  |
| 249 | 6.5 | 27 | 48 | 100 | 4 | 331.821 | 150 | 1.5 | 71.32 | 72.1 | -0.7753 |  |
| 250 | 5 | 30 | 24 | 150 | 5 | 600 | 200 | 1 | 54.24 | 53.69 | 0.5539 |  |
| 251 | 8 | 24 | 72 | 150 | 5 | 600 | 200 | 1 | 56.24 | 48.61 | 7.63 |  |
| 252 | 6.5 | 27 | 48 | 100 | 4 | 500 | 150 | 1.5 | 68 | 73.78 | -5.78 |  |
| 253 | 8 | 24 | 72 | 50 | 3 | 400 | 100 | 2 | 48.04 | 47.26 | 0.7801 |  |
| 254 | 8 | 30 | 24 | 50 | 5 | 600 | 100 | 2 | 46.35 | 48.03 | -1.68 |  |
| 255 | 5 | 30 | 24 | 50 | 3 | 600 | 100 | 1 | 56.24 | 54.12 | 2.12 |  |
| 256 | 5 | 30 | 72 | 50 | 3 | 600 | 100 | 2 | 56.77 | 56.44 | 0.3257 |  |
| 257 | 8 | 24 | 24 | 150 | 3 | 600 | 100 | 2 | 48.01 | 48.57 | -0.5552 |  |
| 258 | 8 | 24 | 72 | 150 | 3 | 600 | 100 | 1 | 46.87 | 47.06 | -0.1898 |  |
| 259 | 5 | 24 | 24 | 50 | 5 | 600 | 100 | 1 | 54.45 | 53.43 | 1.02 |  |
| 260 | 5 | 24 | 72 | 150 | 5 | 400 | 200 | 1 | 54.76 | 54.77 | -0.0086 |  |
| 261 | 5 | 30 | 72 | 50 | 5 | 600 | 100 | 2 | 54.27 | 56.24 | -1.97 |  |
| 262 | 8 | 24 | 24 | 150 | 3 | 600 | 200 | 1 | 46.98 | 47.47 | -0.4949 |  |
| 263 | 8 | 30 | 24 | 150 | 3 | 400 | 100 | 1 | 48.75 | 45.95 | 2.8 |  |
| 264 | 5 | 30 | 24 | 150 | 5 | 600 | 100 | 2 | 56.37 | 56.27 | 0.1043 |  |
| 265 | 5 | 30 | 24 | 50 | 3 | 400 | 200 | 1 | 54.78 | 53.63 | 1.15 |  |
| 266 | 8 | 30 | 72 | 50 | 5 | 600 | 100 | 2 | 48.21 | 48.33 | -0.1229 |  |
| 267 | 8 | 24 | 24 | 150 | 3 | 600 | 200 | 2 | 48.34 | 48.43 | -0.0867 |  |
| 268 | 8 | 30 | 72 | 150 | 5 | 400 | 200 | 2 | 47.57 | 47.06 | 0.5065 |  |
| 269 | 8 | 24 | 72 | 50 | 5 | 400 | 100 | 2 | 46.37 | 47.69 | -1.32 |  |
| 270 | 8 | 24 | 72 | 50 | 5 | 400 | 100 | 1 | 46.37 | 47.29 | -0.9156 |  |
| 271 | 8 | 30 | 72 | 150 | 5 | 400 | 100 | 2 | 44.12 | 47.8 | -3.68 |  |
| 272 | 8 | 30 | 72 | 150 | 3 | 600 | 100 | 2 | 48.74 | 47.39 | 1.35 |  |
| 273 | 5 | 30 | 24 | 150 | 3 | 600 | 100 | 2 | 52.34 | 56.78 | -4.44 |  |
| 274 | 8 | 24 | 24 | 150 | 5 | 600 | 200 | 2 | 48.04 | 47.97 | 0.0728 |  |
| 275 | 5 | 30 | 24 | 150 | 5 | 600 | 100 | 1 | 54.32 | 54.05 | 0.2726 |  |
| 276 | 8 | 30 | 72 | 150 | 3 | 400 | 100 | 1 | 42.1 | 45.35 | -3.25 |  |
| 277 | 8 | 30 | 72 | 150 | 5 | 600 | 100 | 1 | 44.02 | 46.54 | -2.52 |  |
| 278 | 8 | 24 | 72 | 50 | 5 | 400 | 200 | 1 | 44.52 | 47.74 | -3.22 |  |
| 279 | 8 | 30 | 72 | 150 | 5 | 400 | 200 | 1 | 45.02 | 46.55 | -1.53 |  |
| 280 | 8 | 24 | 24 | 50 | 5 | 400 | 100 | 2 | 48.21 | 47.58 | 0.6266 |  |
| 281 | 5 | 24 | 24 | 150 | 3 | 400 | 100 | 1 | 52.74 | 54.62 | -1.88 |  |
| 282 | 6.5 | 27 | 48 | 100 | 4 | 500 | 150 | 1.5 | 72 | 73.78 | -1.78 |  |

**Supplementary Table 3. Analysis of equilibrium Cd (II) biosorption capacity of *Candida tropicalis* XTA 1874 by Langmuir Isotherm at 25℃ [Mean (n)=6, n±SEM]**

| **Experiment No.** | $\boldsymbol{C}_{\boldsymbol{0}}\boldsymbol{(ppm)}$ | $\boldsymbol{C}_{\boldsymbol{e}}\boldsymbol{(ppm)}$ | $\frac{\boldsymbol{1}}{\boldsymbol{C}_{\boldsymbol{e}}}$ | $\boldsymbol{log}\boldsymbol{C}_{\boldsymbol{e}}$ | **Adsorbed**  **Cd(II), ppm** | **Absorbed**  **Cd(II),ppm** | **Intracellular Cd(II) , mg/g** | $\boldsymbol{q}_{\boldsymbol{e}}\boldsymbol{(mg}\boldsymbol{g}^{\boldsymbol{-1}}\boldsymbol{)}$ | $\frac{\boldsymbol{1}}{\boldsymbol{q}_{\boldsymbol{e}}}$ | $\boldsymbol{log}\boldsymbol{C}_{\boldsymbol{e}}$ |  | **Mean Equilibrium Biosorption capacity (%)** | **Pooled Mean Equilibrium Biosorption capacity (%)** |
| --- | --- | --- | --- | --- | --- | --- | --- | --- | --- | --- | --- | --- | --- |
| 1 | 15 | 4.6±0.000 | 0.2174 | 0.841 | 10.4±0.001 | 0.00016±0.000 | 0.001±0.001 | 7.801±0.001 | 0.128 | 0.841 |  | 69.334±0.000 |  |
| 2 | 25 | 7.203±0.002 | 0.138 | 1.074 | 17.8±0.000 | 0.0006±0.001 | 0.006±0.001 | 13.865±0.000 | 0.072 | 1.074 |  | 71.2±0.000 |  |
| 3 | 30 | 12.6±0.001 | 0.079 | 1.064 | 17.402±17.402 | 0.0001±0.000 | 0.011±0.002 | 16.263±0.001 | 0.061 | 1.064 |  | 58±0.0001 | 66.327±0.001 |
| 4 | 45 | 15.55±0.000 | 0.064 | 1.293 | 29.45±7.72E^-05^ | 0.0001±0.000 | 0.022±0.005 | 22.601±0.001 | 0.044 | 1.293 |  | 65.442±0.003 |  |
| 5 | 55 | 25.65±0.000 | 0.038 | 1.292 | 29.35±0.000 | 0.0002±0.000 | 0.012±0.002 | 28.664±0.002 | 0.035 | 1.292 |  | 53.366±0.001 |  |
| 6 | 100 | 33.33±0.000 | 0.030 | 1.648 | 66.669±2.17E^-05^ | 0.001±7.36E^-19^ | 0.012±0.002 | 52.597±0.001 | 0.019 | 1.648 |  | 66.658±0.011 |  |
| 7 | 250 | 65.05±0.000 | 0.015 | 2.091 | 184.949±3.04E^-05^ | 0.001±9.55 E^-05^ | 0.025±0.005 | 123.754±0.001 | 0.008 | 2.091 |  | 73.98±0.000 |  |
| 8 | 300 | 111.02±0.000 | 0.009 | 2.1 | 188.978±0.000 | 0.001±2.01 E^-06^ | 0.027±0.006 | 126.217±0.001 | 0.008 | 2.1 |  | 62.994±0.001 |  |
| 9 | 500 | 122.45±0.000 | 0.008 | 2.4 | 377.548±1.43E^-07^ | 0.001±5.976E^-06^ | 0.021±0.004 | 251.879±0.000 | 0.004 | 2.401 |  | 75.507±0.003 |  |

**Supplementary Table 4. Analysis of equilibrium Cd (II) biosorption capacity of *Candida tropicalis* XTA 1874 by Langmuir Isotherm at 26℃ [Mean (n)=6, n±SEM]**

| **Experiment No.** | $\boldsymbol{C}_{\boldsymbol{0}}\boldsymbol{(ppm)}$ | $\boldsymbol{C}_{\boldsymbol{e}}\boldsymbol{(ppm)}$ | $\frac{\boldsymbol{1}}{\boldsymbol{C}_{\boldsymbol{e}}}$ | $\boldsymbol{log}\boldsymbol{C}_{\boldsymbol{e}}$ | **Adsorbed**  **Cd(II), ppm** | **Absorbed**  **Cd(II),ppm** | **Intracellular Cd(II) , mg/g** | $\boldsymbol{q}_{\boldsymbol{e}}\boldsymbol{(mg}\boldsymbol{g}^{\boldsymbol{-1}}\boldsymbol{)}$ | $\frac{\boldsymbol{1}}{\boldsymbol{q}_{\boldsymbol{e}}}$ | $\boldsymbol{log}\boldsymbol{C}_{\boldsymbol{e}}$ |  | **Mean Equilibrium Biosorption capacity (%)** | **Pooled Mean Equilibrium Biosorption capacity (%)** |
| --- | --- | --- | --- | --- | --- | --- | --- | --- | --- | --- | --- | --- | --- |
| 1 | 15 | 3.3±0.001 | 0.2174 | 0.518 | 11.701±0.001 | 0.0005±0.001 | 0.001±0.001 | 7.869±0.001 | 0.127 | 0.892 |  | 78.004±0.004 |  |
| 2 | 25 | 4.201±0.001 | 0.138 | 0.623 | 20.798±0.001 | 0.002±4.97E^-06^ | 0.006±0.001 | 13.641±0.000 | 0.073 | 1.142 |  | 83.206±0.005 |  |
| 3 | 30 | 5.601±0.001 | 0.079 | 0.748 | 24.395±0.001 | 0.004±4.14E^-05^ | 0.012±0.002 | 16.521±0.001 | 0.061 | 1.211 |  | 81.333±7.39E^-05^ | 74.221±0.000 |
| 4 | 45 | 10.95±0.000 | 0.064 | 1.039 | 33.905±0.004 | 0.149±0.0002 | 0.039±0.012 | 22.775±0.000 | 0.044 | 1.354 |  | 75.333±5.22E^-05^ |  |
| 5 | 55 | 11.84±0.000 | 0.038 | 1.073 | 42.994±3.133E^-15^ | 0.166±9.89E^-05^ | 0.03±0.016 | 28.944±0.002 | 0.035 | 1.457 |  | 78.171±1.4E^-05^ |  |
| 6 | 100 | 20.93±0.000 | 0.030 | 1.321 | 78.895±0.001 | 0.175±8.55 E^-05^ | 0.031±0.017 | 52.828±0.001 | 0.018 | 1.721 |  | 78.91±0.016 |  |
| 7 | 250 | 64.15±0.000 | 0.015 | 1.807 | 185.637±0.005 | 0.218±1.65 E^-15^ | 0.049±0.019 | 124.672±0.001 | 0.008 | 2.093 |  | 74.264±0.012 |  |
| 8 | 300 | 110.45±0.000 | 0.009 | 2.043 | 189.327±0.002 | 0.225±3.09 E^-07^ | 0.053±0.019 | 126.438±0.001 | 0.008 | 2.101 |  | 63.108±0.001 |  |
| 9 | 500 | 121.95±0.001 | 0.008 | 2.086 | 377.818±0.001 | 0.23±8.18 E^-05^ | 0.145±0.011 | 252.395±0.000 | 0.004 | 2.401 |  | 75.564±0.0001 |  |

**Supplementary Table 5. Analysis of equilibrium Cd (II) biosorption capacity of *Candida tropicalis* XTA 1874 by Langmuir Isotherm at 27℃ [Mean (n)=6, n±SEM]**

| **Experiment No.** | $\boldsymbol{C}_{\boldsymbol{0}}\boldsymbol{(ppm)}$ | $\boldsymbol{C}_{\boldsymbol{e}}\boldsymbol{(ppm)}$ | $\frac{\boldsymbol{1}}{\boldsymbol{C}_{\boldsymbol{e}}}$ | $\boldsymbol{log}\boldsymbol{C}_{\boldsymbol{e}}$ | **Adsorbed**  **Cd(II), ppm** | **Absorbed**  **Cd(II),ppm** | **Intracellular Cd(II) , mg/g** | $\boldsymbol{q}_{\boldsymbol{e}}\boldsymbol{(mg}\boldsymbol{g}^{\boldsymbol{-1}}\boldsymbol{)}$ | $\frac{\boldsymbol{1}}{\boldsymbol{q}_{\boldsymbol{e}}}$ | $\boldsymbol{log}\boldsymbol{C}_{\boldsymbol{e}}$ |  | **Mean Equilibrium Biosorption capacity (%)** | **Pooled Mean Equilibrium Biosorption capacity (%)** |
| --- | --- | --- | --- | --- | --- | --- | --- | --- | --- | --- | --- | --- | --- |
| 1 | 15 | 3.214± 0.008 | 0.311 | 0.505 | 11.803±0.00 | 0.0002±0.000 | 0.006±0.001 | 7.869±0.005 | 0.127 | 0.895 |  | 78.667±0.000 |  |
| 2 | 25 | 4.499± 0.000 | 0.222 | 0.623 | 20.79±0.000 | 0.01±1.23E^-04^ | 0.007±0.000 | 13.641±0.236 | 0.073 | 1.142 |  | 83.2±0.000 |  |
| 3 | 30 | 6.197± 0.002 | 0.161 | 0.716 | 24.78±0.000 | 0.02±4.937E^-05^ | 0.013±0.000 | 16.52±0.001 | 0.061 | 1.218 |  | 82.666±0.001 | 74.579±0.004 |
| 4 | 45 | 10.804± 0.003 | 0.093 | 1.034 | 34.16±0.000 | 0.02±0.0004 | 0.025±0.002 | 22.775±0.002 | 0.044 | 1.357 |  | 75.91±0.001 |  |
| 5 | 55 | 11.16± 0.000 | 0.089 | 1.066 | 43.329±0.000 | 0.02±4.462E^-05^ | 0.014±0.001 | 28.944±0.040 | 0.035 | 1.461 |  | 78.782±0.000 |  |
| 6 | 100 | 20.32± 0.000 | 0.049 | 1.318 | 79.15±0.000 | 0.02±3.257E^-06^ | 0.015±0.0002 | 52.828±0.043 | 0.018 | 1.722 |  | 79.15±0.003 |  |
| 7 | 250 | 62.34± 0.001 | 0.016 | 1.799 | 186.92±0.000 | 0.03±1.713 E^-09^ | 0.028±0.002 | 124.672±0.041 | 0.008 | 2.095 |  | 74.768±0.000 |  |
| 8 | 300 | 109.231±0.001 | 0.009 | 2.043 | 189.62±0.000 | 0.03±1.714E^-09^ | 0.031±0.003 | 126.438±0.019 | 0.008 | 2.102 |  | 63.21±0.001 |  |
| 9 | 500 | 120.14±0.000 | 0.008 | 2.085 | 378.42±0.000 | 0.04±0.001 | 0.024±0.001 | 252.395±0.054 | 0.004 | 2.402 |  | 75.682±0.002 |  |

**Supplementary Table 6. Analysis of equilibrium Cd (II) biosorption capacity of *Candida tropicalis* XTA 1874 by Langmuir Isotherm at 28℃ [Mean (n)=6, n±SEM]**

| **Experiment No.** | $\boldsymbol{C}_{\boldsymbol{0}}\boldsymbol{(ppm)}$ | $\boldsymbol{C}_{\boldsymbol{e}}\boldsymbol{(ppm)}$ | $\frac{\boldsymbol{1}}{\boldsymbol{C}_{\boldsymbol{e}}}$ | $\boldsymbol{log}\boldsymbol{C}_{\boldsymbol{e}}$ | **Adsorbed**  **Cd(II), ppm** | **Absorbed**  **Cd(II),ppm** | **Intracellular Cd(II) , mg/g** | $\boldsymbol{q}_{\boldsymbol{e}}\boldsymbol{(mg}\boldsymbol{g}^{\boldsymbol{-1}}\boldsymbol{)}$ | $\frac{\boldsymbol{1}}{\boldsymbol{q}_{\boldsymbol{e}}}$ | $\boldsymbol{log}\boldsymbol{C}_{\boldsymbol{e}}$ |  | **Mean Equilibrium Biosorption capacity (%)** | **Pooled Mean Equilibrium Biosorption capacity (%)** |
| --- | --- | --- | --- | --- | --- | --- | --- | --- | --- | --- | --- | --- | --- |
| 1 | 15 | 3.201± 0.001 | 0.312 | 0.895 | 11.792± 0.000 | 0.0081± 0.000 | 0.005±0.0001 | 7.869±0.005 | 0.127 | 0.505 |  | 78.668±0.001 |  |
| 2 | 25 | 4.5± 0.000 | 0.222 | 1.135 | 20.493± 0.001 | 0.0083±3.2E^-04^ | 0.006±0.000 | 13.641±0.236 | 0.073 | 0.653 |  | 82.006±0.007 |  |
| 3 | 30 | 5.5± 0.0003 | 0.182 | 1.213 | 24.492± 0.000 | 0.008±0.0002 | 0.029±0.011 | 16.521±0.001 | 0.061 | 0.74 |  | 81.672±0.005 | 74.381±0.008 |
| 4 | 45 | 10.939± 0.000 | 0.091 | 1.356 | 34.052± 0.000 | 0.0082±0.0003 | 0.046±0.008 | 22.775±0.002 | 0.044 | 1.039 |  | 75.671±0.000 |  |
| 5 | 55 | 11.829± 0.000 | 0.085 | 1.458 | 43.16± 0.000 | 0.011±0.0002 | 0.046±0.012 | 28.944±0.040 | 0.035 | 1.073 |  | 78.476±0.004 |  |
| 6 | 100 | 20.919± 0.000 | 0.048 | 1.722 | 79.07± 0.001 | 0.01±5.15E^-04^ | 0.056±0.009 | 52.828±0.043 | 0.019 | 1.321 |  | 79.078±0.006 |  |
| 7 | 250 | 64.14± 0.000 | 0.015 | 2.093 | 185.841± 0.000 | 0.02±3.47 E^-04^ | 0.014±0.000 | 124.672±0.041 | 0.008 | 1.807 |  | 74.337±0.001 |  |
| 8 | 300 | 110.43± 0.000 | 0.009 | 2.102 | 189.54± 0.000 | 0.03±3.43E^-04^ | 0.02±0.000 | 126.438±0.019 | 0.008 | 2.043 |  | 63.183±0.003 |  |
| 9 | 500 | 121.93± 0.000 | 0.008 | 2.401 | 378.04± 0.000 | 0.0305±0.0005 | 0.022±0.001 | 252.318±0.041 | 0.004 | 2.086 |  | 75.608±0.000 |  |

**Supplementary Table 7. Values of the adsorption equilibrium isotherm model and thermodynamic parameters for Cd(II) in *Candida tropicalis* XTA 1874 [Mean (n)=6, n±SEM]**

| **Langmuir** | $\boldsymbol{q}_{\boldsymbol{max}}$  **(**$\boldsymbol{mg}\boldsymbol{g}^{\boldsymbol{-}\boldsymbol{1}}$**)** | $\boldsymbol{K}_{\boldsymbol{l}}$  $\left( \boldsymbol{Lm}\boldsymbol{g}^{\boldsymbol{-1}} \right)$ | $\boldsymbol{R}^{\boldsymbol{2}}$ | $\boldsymbol{R}_{\boldsymbol{l}}$ | **Mean Removal (%)** | $\boldsymbol{\Delta}\boldsymbol{G}^{\boldsymbol{0}}$  $\left( \boldsymbol{kJmo}\boldsymbol{l}^{\boldsymbol{-1}} \right)$ | $\boldsymbol{\Delta}\boldsymbol{H}^{\boldsymbol{0}}$  $\left( \boldsymbol{kJ} \boldsymbol{mo}\boldsymbol{l}^{\boldsymbol{-}\boldsymbol{1}} \right)$ |  | $\boldsymbol{\Delta}\boldsymbol{S}^{\boldsymbol{0}}$  $\left( \boldsymbol{J} \boldsymbol{K}^{\boldsymbol{-}\boldsymbol{1}}\boldsymbol{mo}\boldsymbol{l}^{\boldsymbol{-}\boldsymbol{1}} \right)$ |  |
| --- | --- | --- | --- | --- | --- | --- | --- | --- | --- | --- |
| 25℃ | 233.579±2.711 | 1.016±0.233 | 0.968 | 0.002±0.001- 0.062±0.000 | 66.331±0.003 | -0.039±0.000 | 47.574±0.002 |  | 159.848±0.004 |  |
| 26℃ | 420.111±3.15 | 1.113±0.243 | 0.977 | 0.0019±0.000- 0.055±0.000 | 74.220±0.000 | -0.265±0.008 |  |  |  |  |
| 27℃ | 544.113±6.071 | 1.155±0.273 | 0.978 | 0.0018±0.000- 0.055±0.000 | 74.584±0.001 | -0.358±0.000 |  |  |  |  |
| 28℃ | 468.812±8.755 | 1.129±0.264 | 0.972 | 0.0021±0.000- 0.055±0.000 | 74.381±0.008 | -0.344±0.001 |  |  |  |  |
| **Freundlich** | $\boldsymbol{K}_{\boldsymbol{f}}$ ($\boldsymbol{Lm}\boldsymbol{g}^{\boldsymbol{-}\boldsymbol{1}}$**)** | $\boldsymbol{n}$ | $\boldsymbol{R}^{\boldsymbol{2}}$ |  |  |  |  |  |  |  |
| 25℃ | 1.576±0.000 | 1.151±0.001 | 0.962 |  |  |  |  |  |  |  |
| 26℃ | 3.557±0.002 | 1.145±0.007 | 0.972 |  |  |  |  |  |  |  |
| 27℃ | 3.226±0.044 | 1.145±0.006 | 0.973 |  |  |  |  |  |  |  |
| 28℃ | 3.532±0.000 | 1.151±0.000 | 0.973 |  |  |  |  |  |  |  |

**Supplementary Table 8. Kinetic analysis of Cd(II) biosorption using 100ppm Cd(II) by *Candida tropicalis* XTA 1874 at 25℃**

| **Experiment No.** | **Time (min)** | $\boldsymbol{C}_{\boldsymbol{0}}\boldsymbol{(ppm)}$ | $\boldsymbol{C}_{\boldsymbol{e}}\boldsymbol{(ppm)}$ | $\boldsymbol{C}_{\boldsymbol{0}}\boldsymbol{-}\boldsymbol{C}_{\boldsymbol{e}}$ | **Adsorbed**  **Cd(II), ppm** | **Absorbed**  **Cd(II),ppm** | **Intracellular Cd(II) , mg/g** | $\boldsymbol{q}_{\boldsymbol{t}}\boldsymbol{(mg}\boldsymbol{g}^{\boldsymbol{-1}}\boldsymbol{)}$ | $\boldsymbol{t/q}_{\boldsymbol{t}}$ | $\boldsymbol{ln(q}_{\boldsymbol{e}}\boldsymbol{-}\boldsymbol{q}_{\boldsymbol{t}}\boldsymbol{)}$ |
| --- | --- | --- | --- | --- | --- | --- | --- | --- | --- | --- |
| 1 | 0 | 0 | 0 | 0 | 0 | 0 | 0 | 0 | 0 | 0 |
| 2 | 40 | 500 | 131.26 | 368.74 | 368.72 | 0.02 | 0.013333 | 245.8133 | 0.162725 | 1.772554 |
| 3 | 60 | 500 | 130.58 | 369.42 | 369.39 | 0.03 | 0.02 | 246.26 | 0.243645 | 1.693632 |
| 4 | 80 | 500 | 129.64 | 370.36 | 370.32 | 0.04 | 0.026667 | 246.88 | 0.324044 | 1.572608 |
| 5 | 100 | 500 | 128.82 | 371.18 | 371.12 | 0.06 | 0.04 | 247.4133 | 0.404182 | 1.455323 |
| 6 | 150 | 500 | 128.54 | 371.46 | 371.38 | 0.08 | 0.053333 | 247.5867 | 0.605848 | 1.414039 |
| 7 | 180 | 500 | 127.95 | 372.05 | 371.38 | 0.67 | 0.446667 | 247.5867 | 0.727018 | 1.414039 |
| 8 | 200 | 500 | 127.95 | 372.05 | 371.38 | 0.67 | 0.446667 | 247.5867 | 0.807798 | 1.414039 |
| 9 | 250 | 500 | 127.95 | 372.05 | 371.38 | 0.67 | 0.446667 | 247.5867 | 1.009747 | 1.414039 |
| 10 | 280 | 500 | 127.95 | 372.05 | 371.38 | 0.67 | 0.446667 | 247.5867 | 1.130917 | 1.414039 |
| 11 | 300 | 500 | 127.95 | 372.05 | 371.38 | 0.67 | 0.446667 | 247.5867 | 1.211697 | 1.414039 |
| 12 | 350 | 500 | 127.95 | 372.05 | 371.38 | 0.67 | 0.446667 | 247.5867 | 1.413646 | 1.414039 |
| 13 | 380 | 500 | 127.95 | 372.05 | 371.38 | 0.67 | 0.446667 | 247.5867 | 1.534816 | 1.414039 |
| 14 | 400 | 500 | 127.95 | 372.05 | 371.38 | 0.67 | 0.446667 | 247.5867 | 1.615596 | 1.414039 |
| 15 | 450 | 500 | 127.95 | 372.05 | 371.38 | 0.67 | 0.446667 | 247.5867 | 1.817545 | 1.414039 |
| 16 | 480 | 500 | 127.95 | 372.05 | 371.38 | 0.67 | 0.446667 | 247.5867 | 1.938715 | 1.414039 |
| 17 | 500 | 500 | 127.95 | 372.05 | 371.38 | 0.67 | 0.446667 | 247.5867 | 2.019495 | 1.414039 |

**Supplementary Table 9. Kinetic analysis of Cd(II) biosorption using 100ppm Cd(II) by *Candida tropicalis* XTA 1874 at 26℃**

| **Experiment No.** | **Time (min)** | $\boldsymbol{C}_{\boldsymbol{0}}\boldsymbol{(}\boldsymbol{ppm}\boldsymbol{)}$ | $\boldsymbol{C}_{\boldsymbol{e}}\boldsymbol{(}\boldsymbol{ppm}\boldsymbol{)}$ | $\boldsymbol{C}_{\boldsymbol{0}}\boldsymbol{-}\boldsymbol{C}_{\boldsymbol{e}}$ | **Adsorbed**  **Cd(II), ppm** | **Absorbed**  **Cd(II),ppm** | **Intracellular Cd(II) , mg/g** | $\boldsymbol{q}_{\boldsymbol{t}}\boldsymbol{(}\boldsymbol{mg}\boldsymbol{g}^{\boldsymbol{-}\boldsymbol{1}}\boldsymbol{)}$ | ${\boldsymbol{t}\boldsymbol{/}\boldsymbol{q}}_{\boldsymbol{t}}$ | ${\boldsymbol{ln}\boldsymbol{(}\boldsymbol{q}}_{\boldsymbol{e}}\boldsymbol{-}\boldsymbol{q}_{\boldsymbol{t}}\boldsymbol{)}$ |
| --- | --- | --- | --- | --- | --- | --- | --- | --- | --- | --- |
| 1 | 0 | 0 | 0 | 0 | 0 | 0 | 0 | 0 | 0 | 0 |
| 2 | 40 | 500 | 128.26 | 371.74 | 371.72 | 0.02 | 0.013333 | 247.8133 | 0.161412 | 1.402824 |
| 3 | 60 | 500 | 126.48 | 373.52 | 373.49 | 0.03 | 0.02 | 248.9933 | 0.24097 | 1.060102 |
| 4 | 80 | 500 | 125.24 | 374.76 | 374.72 | 0.04 | 0.026667 | 249.8133 | 0.320239 | 0.725937 |
| 5 | 100 | 500 | 124.22 | 375.78 | 375.72 | 0.06 | 0.04 | 250.48 | 0.399233 | 0.336472 |
| 6 | 150 | 500 | 124.18 | 375.82 | 375.7 | 0.12 | 0.08 | 250.4667 | 0.598882 | 0.345951 |
| 7 | 180 | 500 | 124.12 | 375.88 | 375.7 | 0.18 | 0.12 | 250.4667 | 0.718659 | 0.345951 |
| 8 | 200 | 500 | 124.12 | 375.88 | 375.7 | 0.18 | 0.12 | 250.4667 | 0.798509 | 0.345951 |
| 9 | 250 | 500 | 124.12 | 375.88 | 375.7 | 0.18 | 0.12 | 250.4667 | 0.998137 | 0.345951 |
| 10 | 280 | 500 | 124.12 | 375.88 | 375.7 | 0.18 | 0.12 | 250.4667 | 1.117913 | 0.345951 |
| 11 | 300 | 500 | 124.12 | 375.88 | 375.7 | 0.18 | 0.12 | 250.4667 | 1.197764 | 0.345951 |
| 12 | 350 | 500 | 124.12 | 375.88 | 375.7 | 0.18 | 0.12 | 250.4667 | 1.397392 | 0.345951 |
| 13 | 380 | 500 | 124.12 | 375.88 | 375.7 | 0.18 | 0.12 | 250.4667 | 1.517168 | 0.345951 |
| 14 | 400 | 500 | 124.12 | 375.88 | 375.7 | 0.18 | 0.12 | 250.4667 | 1.597019 | 0.345951 |
| 15 | 450 | 500 | 124.12 | 375.88 | 375.7 | 0.18 | 0.12 | 250.4667 | 1.796646 | 0.345951 |
| 16 | 480 | 500 | 124.12 | 375.88 | 375.7 | 0.18 | 0.12 | 250.4667 | 1.916423 | 0.345951 |
| 17 | 500 | 500 | 124.12 | 375.88 | 375.7 | 0.18 | 0.12 | 250.4667 | 1.996274 | 0.345951 |

**Supplementary Table 10. Kinetic analysis of Cd(II) biosorption using 100ppm Cd(II) by *Candida tropicalis* XTA 1874 at 27℃**

| **Experiment No.** | **Time (min)** | $\boldsymbol{C}_{\boldsymbol{0}}\boldsymbol{(ppm)}$ | $\boldsymbol{C}_{\boldsymbol{e}}\boldsymbol{(ppm)}$ | $\boldsymbol{C}_{\boldsymbol{0}}\boldsymbol{-}\boldsymbol{C}_{\boldsymbol{e}}$ | **Adsorbed**  **Cd(II), ppm** | **Absorbed**  **Cd(II),ppm** | **Intracellular Cd(II) , mg/g** | $\boldsymbol{q}_{\boldsymbol{t}}\boldsymbol{(mg}\boldsymbol{g}^{\boldsymbol{-1}}\boldsymbol{)}$ | $\boldsymbol{t/q}_{\boldsymbol{t}}$ | $\boldsymbol{ln(q}_{\boldsymbol{e}}\boldsymbol{-}\boldsymbol{q}_{\boldsymbol{t}}\boldsymbol{)}$ |
| --- | --- | --- | --- | --- | --- | --- | --- | --- | --- | --- |
| 1 | 0 | 0 | 0 | 0 | 0 | 0 | 0 | 0 | 0 | 0 |
| 2 | 40 | 100 | 25.22 | 74.78 | 74.72 | 0.06 | 0.04 | 49.81333 | 0.802998 | 1.103521 |
| 3 | 60 | 100 | 24.46 | 75.54 | 75.39 | 0.15 | 0.1 | 50.26 | 1.193792 | 0.943164 |
| 4 | 80 | 100 | 23.42 | 76.58 | 76.53 | 0.05 | 0.03333333 | 51.02 | 1.568013 | 0.592274 |
| 5 | 100 | 100 | 22.37 | 77.63 | 77.58 | 0.05 | 0.03333333 | 51.72 | 1.933488 | 0.102643 |
| 6 | 150 | 100 | 21.28 | 78.72 | 78.68 | 0.04 | 0.02666667 | 52.45333 | 2.859685 | -0.98146 |
| 7 | 180 | 100 | 21.28 | 78.72 | 78.68 | 0.04 | 0.02666667 | 52.45333 | 3.431622 | -0.98146 |
| 8 | 200 | 100 | 21.28 | 78.72 | 78.68 | 0.04 | 0.02666667 | 52.45333 | 3.812913 | -0.98146 |
| 9 | 250 | 100 | 21.28 | 78.72 | 78.68 | 0.04 | 0.02666667 | 52.45333 | 4.766141 | -0.98146 |
| 10 | 280 | 100 | 21.28 | 78.72 | 78.68 | 0.04 | 0.02666667 | 52.45333 | 5.338078 | -0.98146 |
| 11 | 300 | 100 | 21.28 | 78.72 | 78.68 | 0.04 | 0.02666667 | 52.45333 | 5.71937 | -0.98146 |
| 12 | 350 | 100 | 21.28 | 78.72 | 78.68 | 0.04 | 0.02666667 | 52.45333 | 6.672598 | -0.98146 |
| 13 | 380 | 100 | 21.28 | 78.72 | 78.68 | 0.04 | 0.02666667 | 52.45333 | 7.244535 | -0.98146 |
| 14 | 400 | 100 | 21.28 | 78.72 | 78.68 | 0.04 | 0.02666667 | 52.45333 | 7.625826 | -0.98146 |
| 15 | 450 | 100 | 21.28 | 78.72 | 78.68 | 0.04 | 0.02666667 | 52.45333 | 8.579054 | -0.98146 |
| 16 | 480 | 100 | 21.28 | 78.72 | 78.68 | 0.04 | 0.02666667 | 52.45333 | 9.150991 | -0.98146 |
| 17 | 500 | 100 | 21.28 | 78.72 | 78.68 | 0.04 | 0.02666667 | 52.45333 | 9.532283 | -0.98146 |

**Supplementary Table 11. Kinetic analysis of Cd(II) Desorption using 281.95 ppm Cd(II)**

| **Experiment No.** | **Time (min)** | $\boldsymbol{q}_{\boldsymbol{i}} \mathbf{(mg}\mathbf{g}^{\mathbf{-1}}\mathbf{)}$ | $\boldsymbol{q}_{\boldsymbol{t}}\boldsymbol{(mg}\boldsymbol{g}^{\boldsymbol{-1}}\boldsymbol{)}$ | $\boldsymbol{C}_{\boldsymbol{a}}\boldsymbol{(ppm)}$ | $\frac{\boldsymbol{1}}{\boldsymbol{C}_{\boldsymbol{a}}}$ | $\boldsymbol{lnt}$ | $\boldsymbol{t}^{\boldsymbol{0.5}}$ |
| --- | --- | --- | --- | --- | --- | --- | --- |
| 1 | 0 | 0 | 0 | 0 | 0 | 0 | 0 |
| 2 | 40 | 187.966 | 0.0224 | 281.9154 | 0.003547 | 3.688879 | 6.324555 |
| 3 | 60 | 187.966 | 0.0182 | 281.9217 | 0.003547 | 4.094345 | 7.745967 |
| 4 | 80 | 187.966 | 0.0143 | 281.9276 | 0.003547 | 4.382027 | 8.944272 |
| 5 | 100 | 187.966 | 0.0126 | 281.9301 | 0.003547 | 4.60517 | 10 |
| 6 | 150 | 187.966 | 0.012 | 281.931 | 0.003547 | 5.010635 | 12.24745 |
| 7 | 180 | 187.966 | 0.012 | 281.931 | 0.003547 | 5.192957 | 13.41641 |
| 8 | 200 | 187.966 | 0.012 | 281.931 | 0.003547 | 5.298317 | 14.14214 |
| 9 | 250 | 187.966 | 0.012 | 281.931 | 0.003547 | 5.521461 | 15.81139 |
| 10 | 280 | 187.966 | 0.012 | 281.931 | 0.003547 | 5.63479 | 16.7332 |
| 11 | 300 | 187.966 | 0.012 | 281.931 | 0.003547 | 5.703782 | 17.32051 |
| 12 | 350 | 187.966 | 0.012 | 281.931 | 0.003547 | 5.857933 | 18.70829 |
| 13 | 380 | 187.966 | 0.012 | 281.931 | 0.003547 | 5.940171 | 19.49359 |
| 14 | 400 | 187.966 | 0.012 | 281.931 | 0.003547 | 5.991465 | 20 |
| 15 | 450 | 187.966 | 0.012 | 281.931 | 0.003547 | 6.109248 | 21.2132 |
| 16 | 480 | 187.966 | 0.012 | 281.931 | 0.003547 | 6.173786 | 21.9089 |
| 17 | 500 | 187.966 | 0.012 | 281.931 | 0.003547 | 6.214608 | 22.36068 |


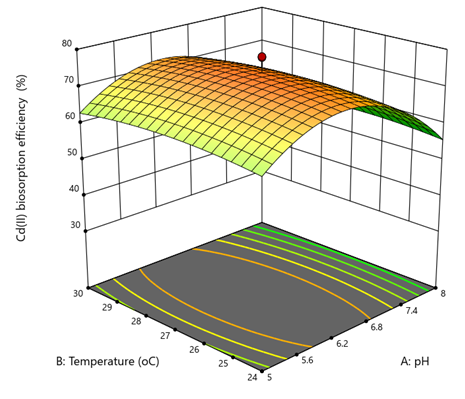


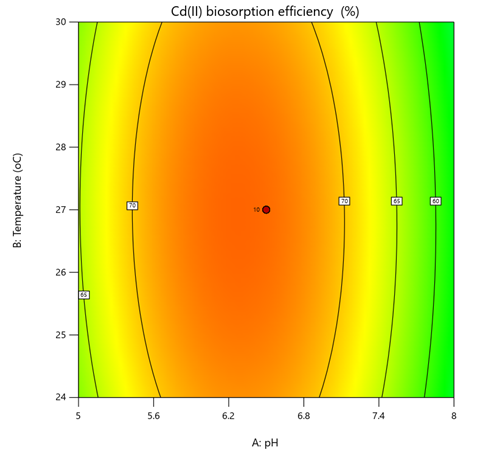


a


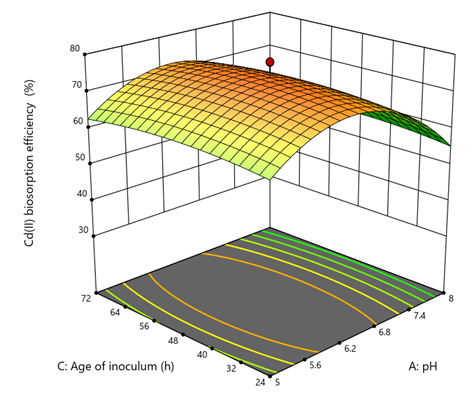

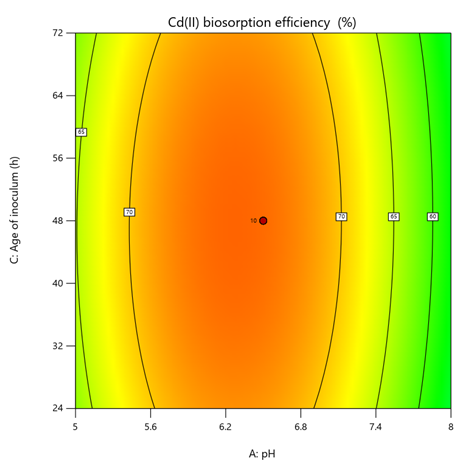


b


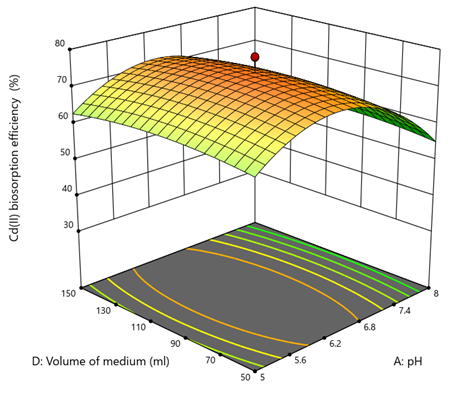

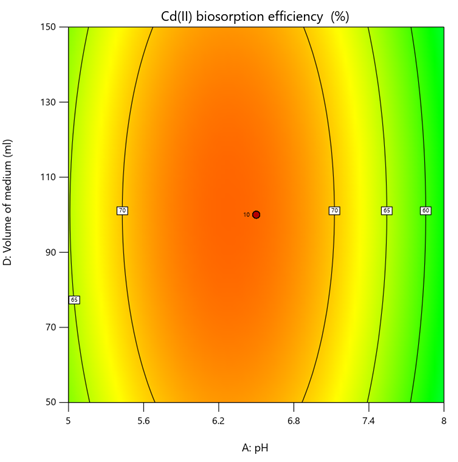


c


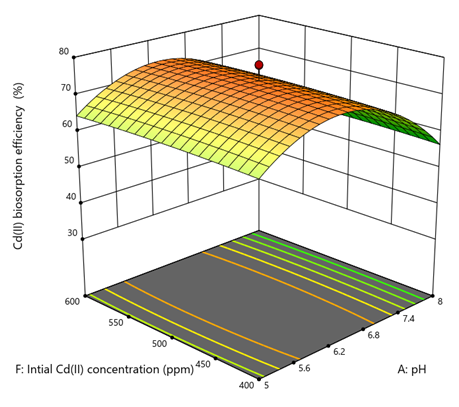

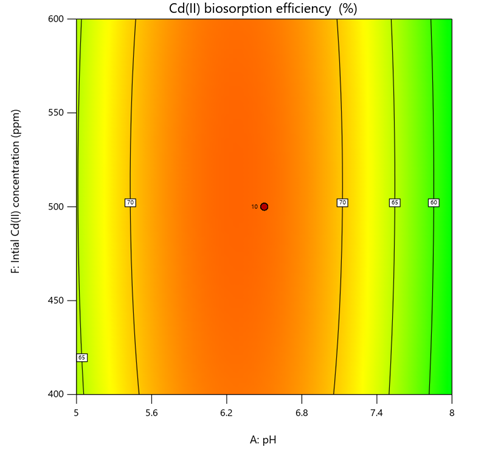


d


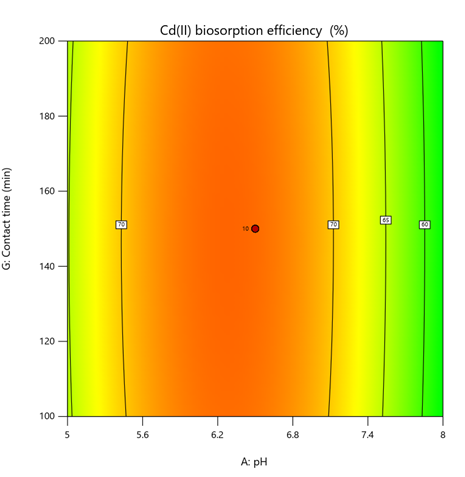

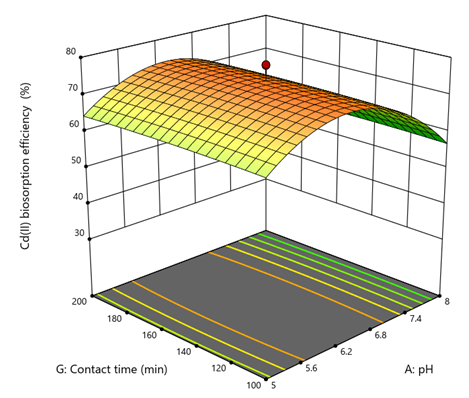


e


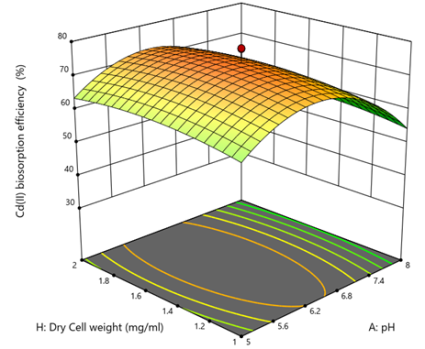

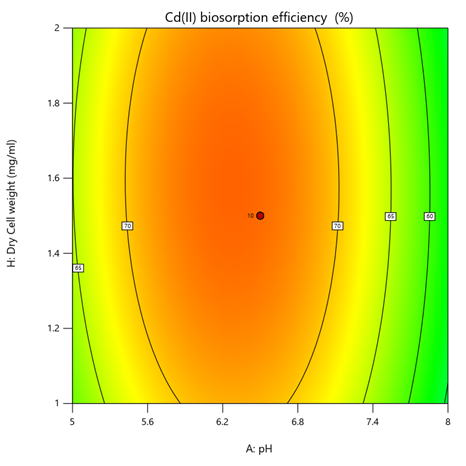


f


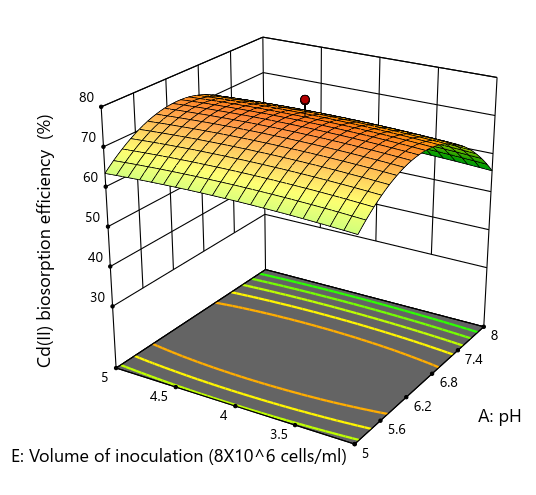

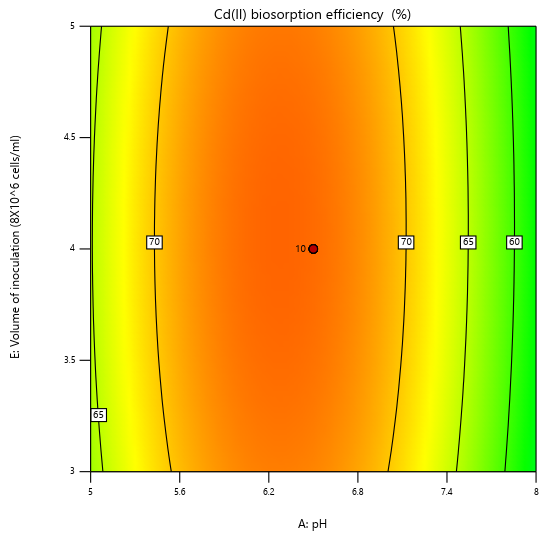


g

**Supplementary Figure 1.** Contour and 3D Response Surface Plots for the effects of (a) pH and Temperature (℃) (b) pH and Age of inoculum (h) (c) pH and Volume of medium (mL) (d) pH and Intial Cd(II) concentration (ppm) (e) pH and Contact time (min) (f) pH and Dry cell weight (mg/mL) (g) pH and Volume of inoculation (8×10^6^ cells/mL) on Cd(II) biosorption efficiency(%) by the live biomass of *Candida tropicalis* XTA1874


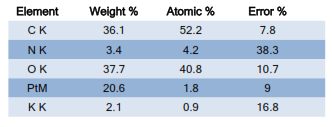

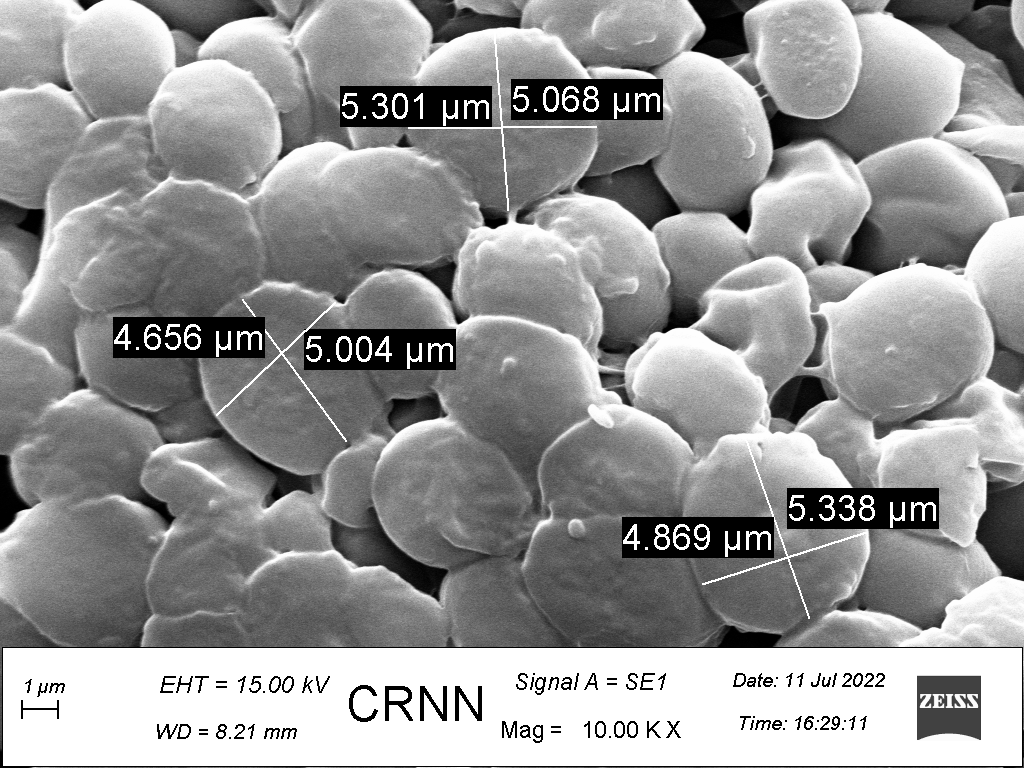


a


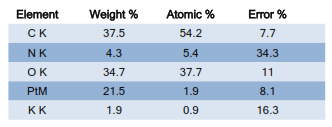

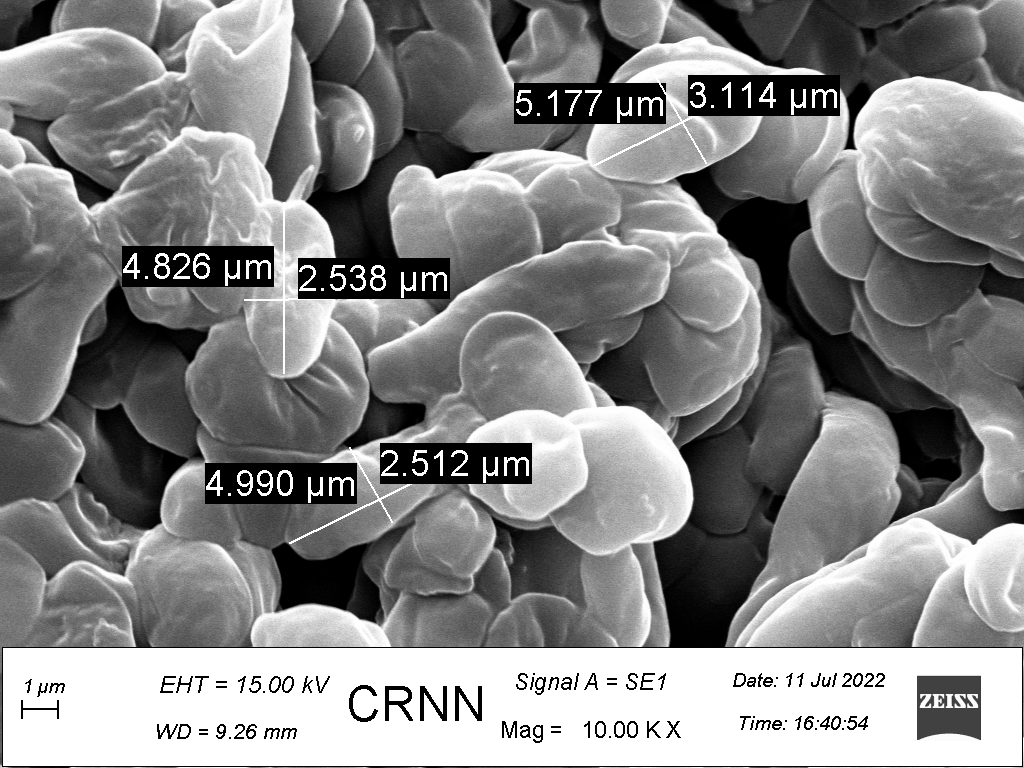


b


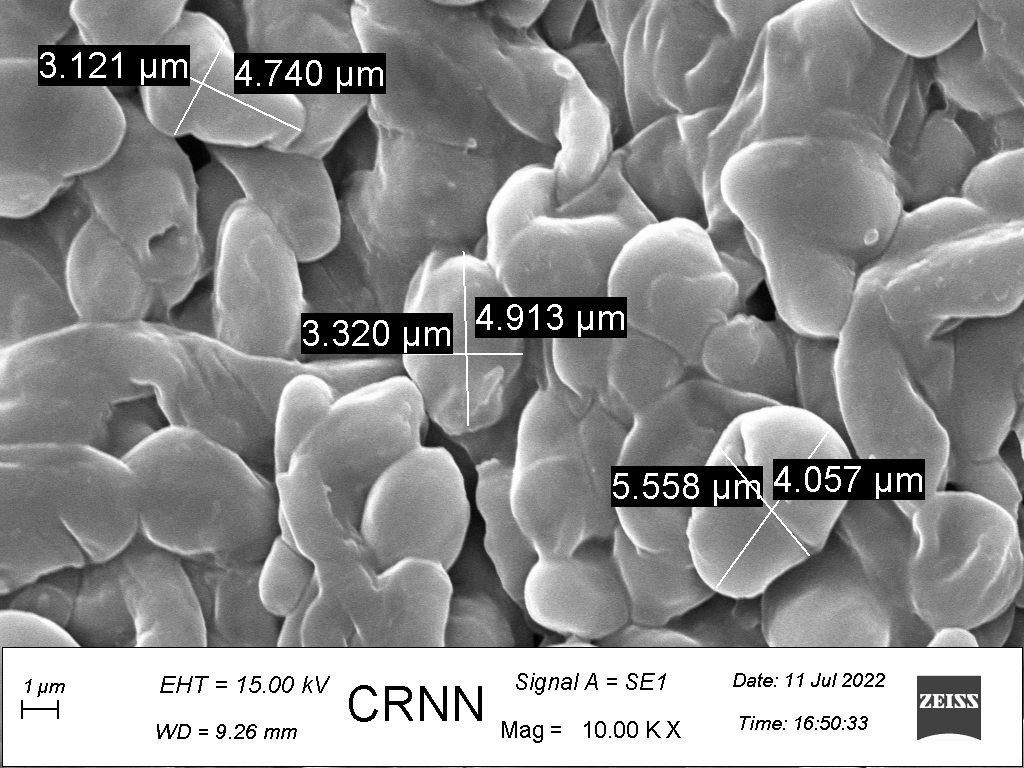

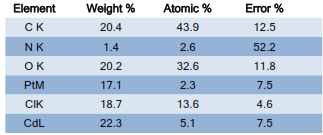


c

**Supplementary Figure 2.** SEM imaging of Cd(II) untreated control isolate (a) Cd (II) untreated resistant (b) and treated resistant strain (c) *Candida tropicalis* XTA 1874


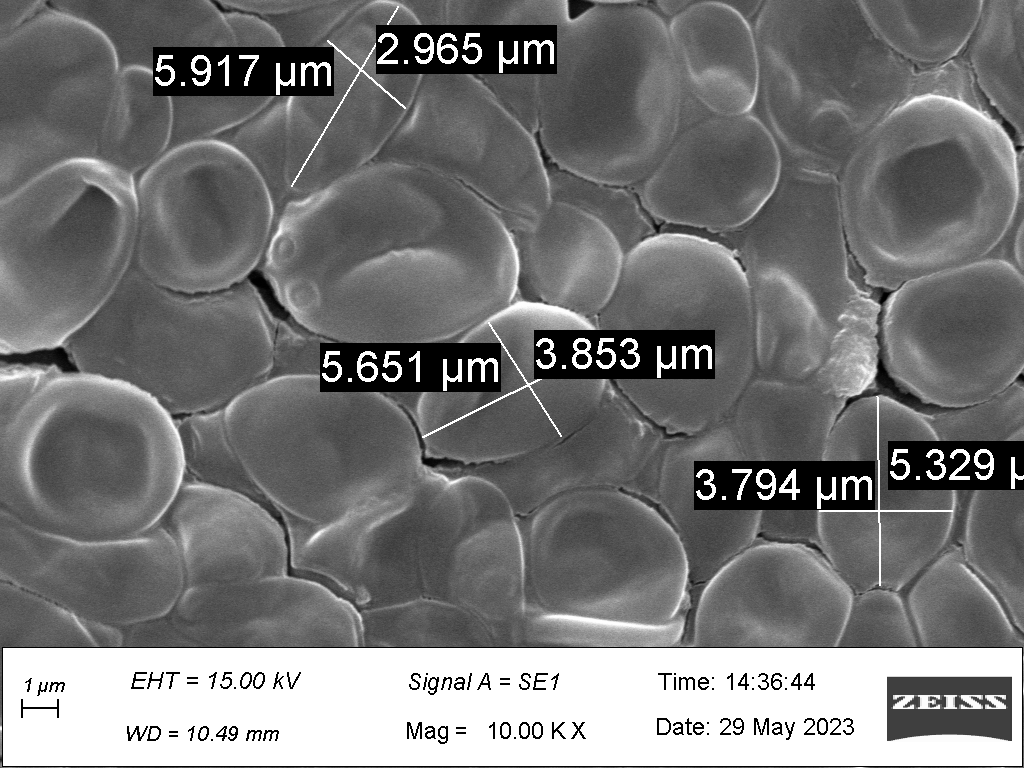

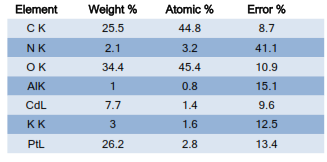

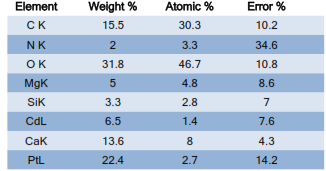

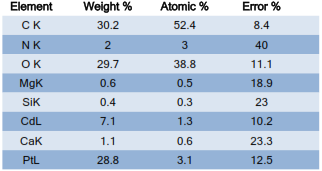

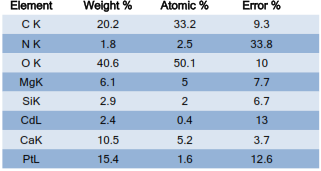

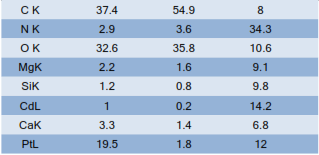

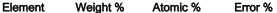


a

b

c

d

e


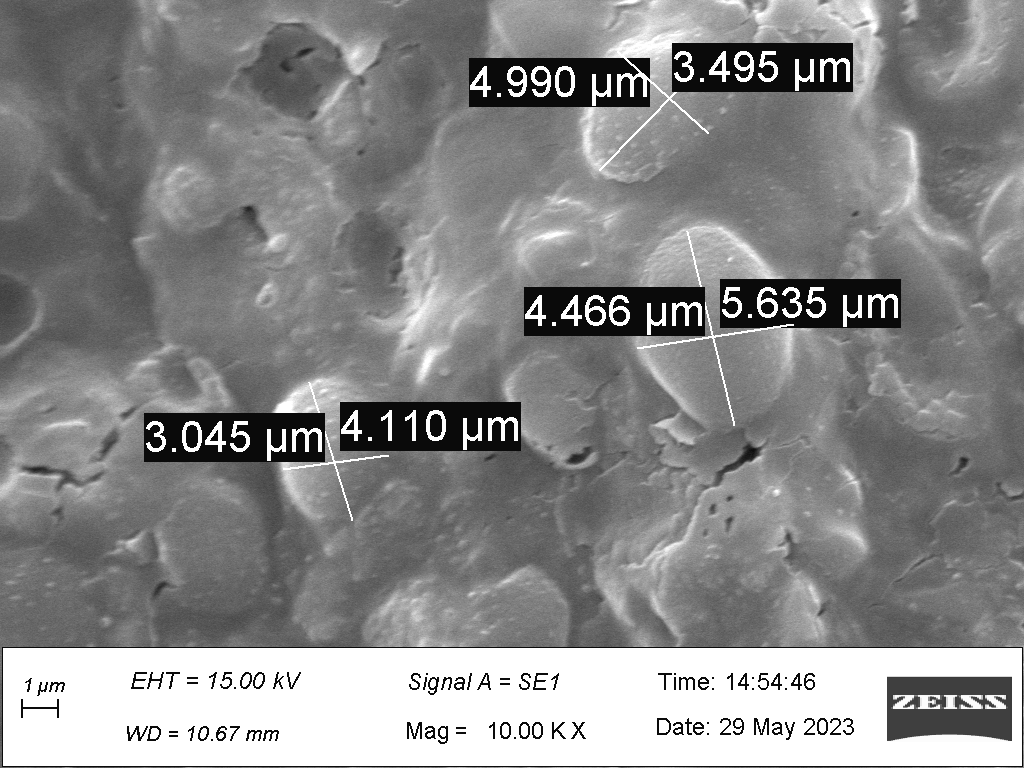

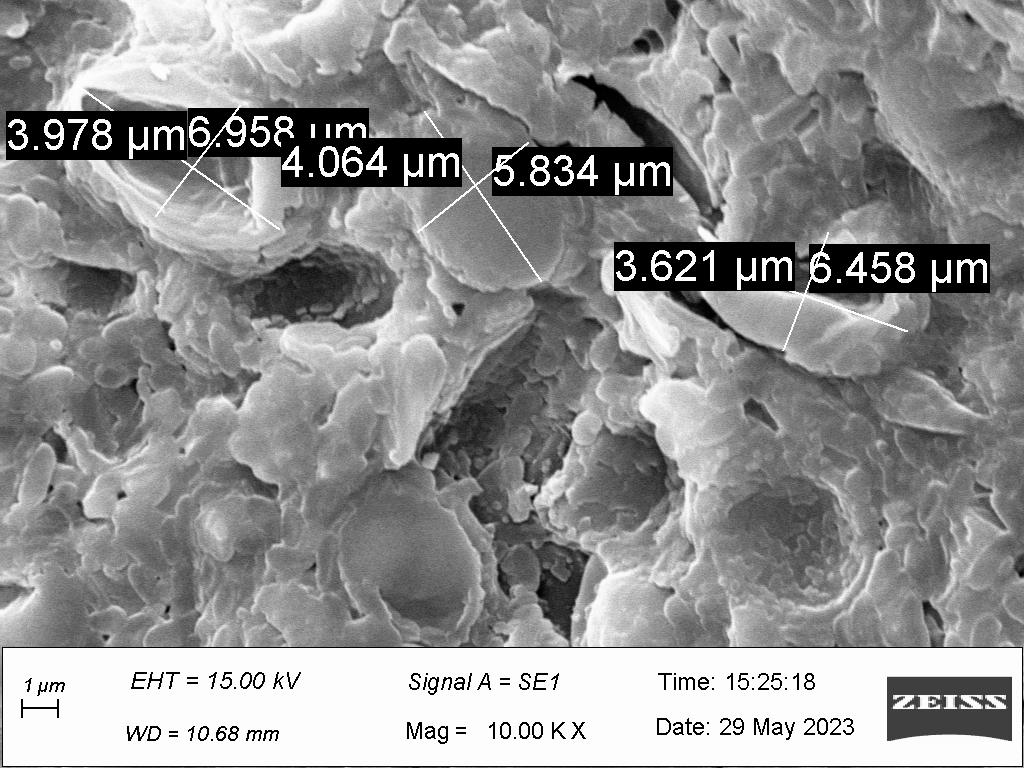

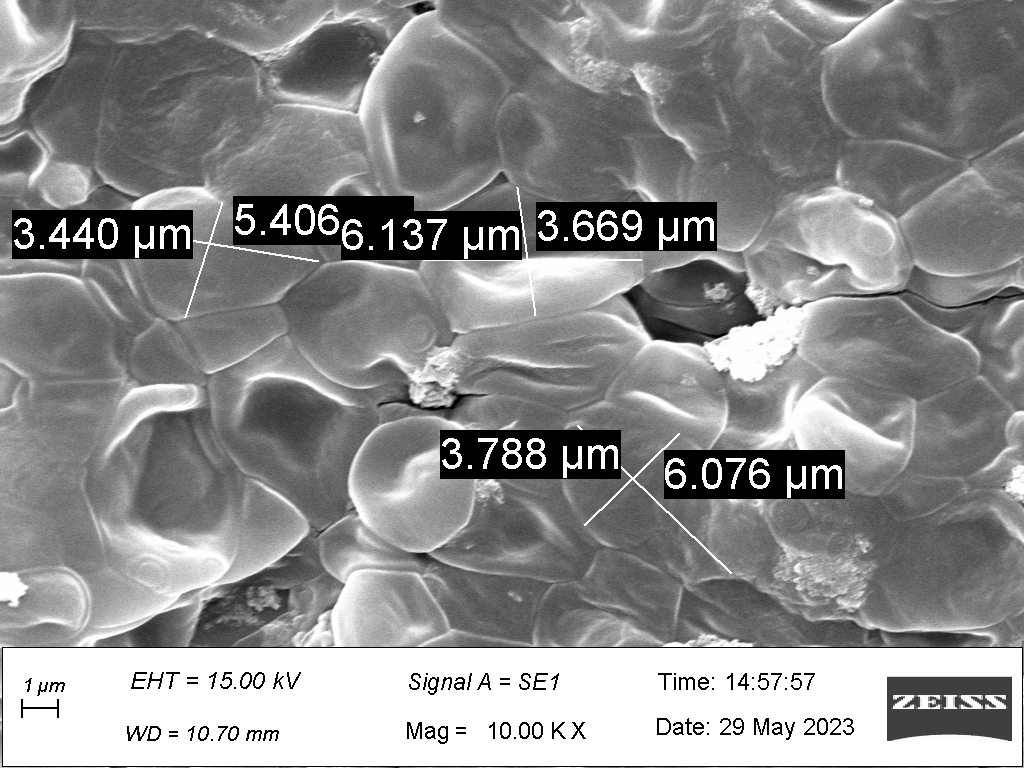

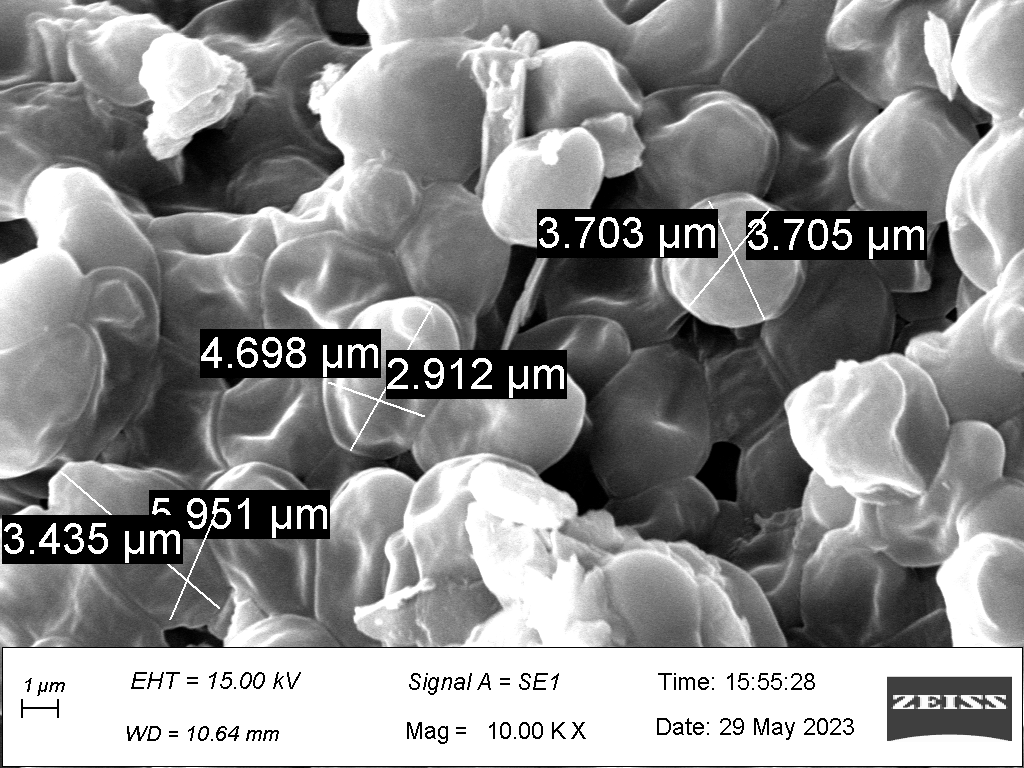


**Supplementary Figure 3.** SEM imaging of enzyme untreated (a) β-Mannanase treated (b) Proteinase K treated (c) Both β-Mannanase and Proteinase K treated (d) Snailase treated (e) Cd(II) resistant strain *Candida tropicalis* XTA 1874


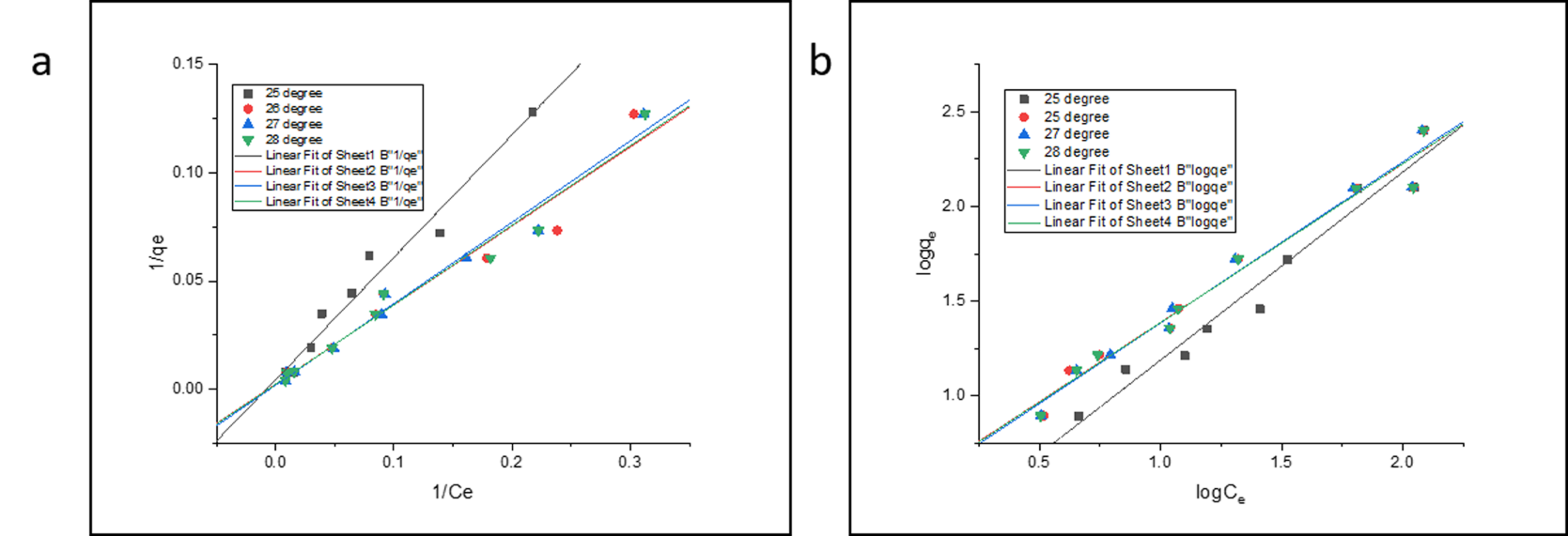


**Supplementary Figure 4.** Adsorption equilibrium isotherm linear plots for Langmuir (a) and Freundlich (b) model for Cd(II) biosorption by the strain *Candida tropicalis* XTA 1874


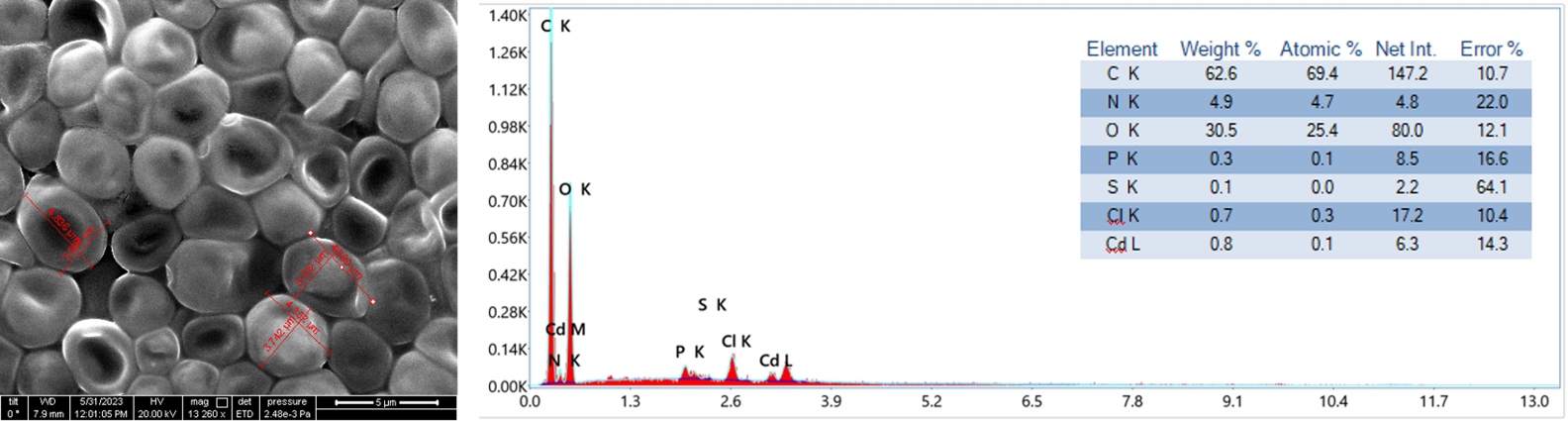


**Supplementary Figure 5.** FE-SEM and EDAX analyses of developed Cd(II) resistant strain *Candida tropicalis* XTA 1874 biomass after Desorption
